# The Effects of Service-Delivery Model and Purchase Price on Hearing-Aid Outcomes in Older Adults: A Randomized Double-Blind Placebo-Controlled Clinical Trial

**DOI:** 10.1044/2017_AJA-16-0111

**Published:** 2017-03

**Authors:** Larry E. Humes, Sara E. Rogers, Tera M. Quigley, Anna K. Main, Dana L. Kinney, Christine Herring

**Affiliations:** a Department of Speech and Hearing Sciences, Indiana University, Bloomington

## Abstract

**Objectives:**

The objectives of this study were to determine efficacy of hearing aids in older adults using audiology best practices, to evaluate the efficacy of an alternative over-the-counter (OTC) intervention, and to examine the influence of purchase price on outcomes for both service-delivery models.

**Design:**

The design of this study was a single-site, prospective, double-blind placebo-controlled randomized trial with three parallel branches: (a) audiology best practices (AB), (b) consumer decides OTC model (CD), and (c) placebo devices (P). Outcome measures were obtained after a typical 6-week trial period with follow-up 4-week AB-based trial for those initially assigned to CD and P groups.

**Setting:**

Older adults from the general community were recruited via newspaper and community flyers to participate at a university research clinic.

**Participants:**

Participants were adults, ages 55–79 years, with mild-to-moderate hearing loss. There were 188 eligible participants: 163 enrolled as a volunteer sample, and 154 completed the intervention.

**Intervention(s):**

All participants received the same high-end digital mini-behind-the-ear hearing aids fitted bilaterally. AB and P groups received best-practice services from audiologists; differing mainly in use of appropriate (AB) or placebo (P) hearing aid settings. CD participants self-selected their own pre-programmed hearing aids via an OTC model.

**Primary and Secondary Outcome Measures:**

Primary outcome measure was a 66-item self-report, Profile of Hearing Aid Benefit (Cox & Gilmore, 1990). Secondary outcome measure was the Connected Speech Test (Cox, Alexander, & Gilmore, 1987) benefit. Additional measures of hearing-aid benefit, satisfaction, and usage were also obtained.

**Results:**

Per-protocol analyses were performed. AB service-delivery model was found to be efficacious for most of the outcome measures, with moderate or large effect sizes (Cohen's *d*). CD service-delivery model was efficacious, with similar effect sizes. However, CD group had a significantly (*p* < .05) lower satisfaction and percentage (CD: 55%; AB: 81%; P: 36%) likely to purchase hearing aids after the trial.

**Conclusions:**

Hearing aids are efficacious in older adults for both AB and CD service-delivery models. CD model of OTC service delivery yielded only slightly poorer outcomes than the AB model. Efficacious OTC models may increase accessibility and affordability of hearing aids for millions of older adults. Purchase price had no effect on outcomes, but a high percentage of those who rejected hearing aids paid the typical price (85%).

**Trial Registration:**

Clinicaltrials.gov: NCT01788432; https://clinicaltrials.gov/ct2/show/NCT01788423

**Supplemental Materials:**

https://doi.org/10.23641/asha.5382499

*This report is dedicated to the memory of Dr. Gordon Blackistone Hughes, MD, clinical trials coordinator for the National Institute on Deafness and Other Communication Disorders (NIDCD) from 2008 to 2015. His guidance and assistance throughout this trial were critical to its success and are greatly appreciated by the authors.*

According to the 2012 National Projections made by the U.S. Census Bureau, about 50 million Americans are over 65 years of age, representing 15% of the U.S. population (Ortman, Velkoff, & Hogan, 2014). Approximately 35% of those over age 65 in the United States, or about 17.5 million older Americans, have a significant hearing loss that is sufficient to make them hearing-aid candidates (Cruickshanks, Zhan, & Zhong, 2010; Schoenborn & Marano, 1988). Yet, only about 20% of those older Americans who could benefit from hearing aids actually seek them out, and of those who do seek them out, only about 50%–70% are satisfied with them and use their hearing aids regularly (Kochkin, 1993a, 1993b, 1993c, 2000, 2005, 2009; Perez & Edmonds, 2012). The large discrepancy between the number of older adults in need of hearing health care and those receiving benefit from hearing aids was one of the motivating factors behind the organization of a National Institute on Deafness and Other Communication Disorders/National Institutes of Health (NIDCD/NIH) working group on accessible and affordable hearing health care for adults with mild-to-moderate hearing loss. The central focus of this meeting was the identification of research areas that would lead to the improvement of hearing health care for adults with mild-to-moderate hearing loss—in particular, enhancing the accessibility and affordability of hearing health care (Donahue, Dubno, & Beck, 2010). The end product of the working group's efforts was the identification and prioritization of several areas in need of additional research that would ultimately lead to the realization of this goal (Donahue et al., 2010). Although the focus was not exclusively on older adults, it is clear that this age group represents the one with the highest prevalence of mild-to-moderate hearing loss and also represents the largest group of hearing aid purchasers, with about two-thirds of the hearing aids sold in the United States being purchased by older adults (e.g., Strom, 2008).

The clinical trial described here addressed one of the high-priority research questions identified by the working group and summarized by Donahue et al. (2010). In particular, this study addressed the following research question identified in that report: “How can current delivery systems (including the system and the provider) be utilized or modified to increase accessibility and affordability of hearing health care?” (para. 40) In addition, this project also addressed several other research questions not designated as high priority but identified as important questions by the working group, including:

- “What is the comparative effectiveness between two delivery schemes (current best practice vs. others), with technology held constant?” (para. 40);
- “What is the minimal hearing health care delivery system needed for successful outcomes and quality care?” (para. 40); and
- “How does an individual define hearing aid value (performance/satisfaction/benefit related to cost)?” (para. 38)

As summarized by Donahue et al. (2010), the price per hearing aid at that time ranged from about $1,200 to $2,900 per device. With two devices typically purchased per person in the United States, this amounts to a cost of $2,400 to $5,800 per person. Assuming a device life expectancy of 5 years, older adults can expect to pay tens of thousands of dollars for hearing health care in their later years. Of course, these are just the purchase prices and do not include significant annual expenditures on maintenance, including the purchase of batteries (~$100/year per person). It should be noted, however, that other hearing-aid delivery models have been developed since the report by Donahue et al. (2010)—for example, delivery through large-volume retailers such as Costco—that have cut purchase prices for the consumer dramatically (e.g., Kasewurm, 2014).

There are two primary components to the average retail price of hearing aids sold in the United States: (a) the cost of the devices themselves from the manufacturer and (b) the fees for services rendered by the professionals delivering the devices. The production costs of most hearing aids are relatively low, although production costs do not include other costs such as research and development, marketing, technical support, and so on. For example, for the devices used in this project—good-quality devices with multiple-channel compression, feedback cancellation, noise reduction, and directional microphones—the production costs were about $100/aid at study onset. Extremely high-volume dispensers of these devices, such as Costco and the Veterans Administration, were charged by the manufacturer about $250–$500 per aid at that time. Wholesale prices to lower-volume dispensers for these devices ranged from about $900–$1,200, and the average retail price charged by these dispensers for these same aids in the United States was about $2,000–$3,000 per aid at that time. Of course, the difference between the dispenser's wholesale price paid and retail price charged is not all profit. The dispenser too has a variety of business expenses to cover. In part, the mark-up from the dispenser's cost to the consumer's purchase price has been justified on the basis of superior outcomes associated with the audiologist-based service-delivery model. No prior study, however, has documented differences in outcomes between the higher-priced audiologist-based service-delivery model and possible lower-priced alternative service-delivery models, including the over-the-counter (OTC), try-and-select service delivery model.

The NIDCD/NIH working group meeting and report sparked broader interest in the accessibility and affordability of hearing health care. The meeting and report provided the impetus for two additional recent influential reports. The first of these two subsequent reports was by the President's Council of Advisors on Science and Technology (PCAST, 2015). This report began by stating,

Untreated hearing loss, especially in older Americans, is a substantial national problem. Only a fraction of consumers who need assistance with hearing obtain and use hearing aids, in large part because of high cost, complex dispensing procedures, social stigma, and performance shortfalls. (p. 1)

The PCAST report went on to identify several problems with the classification of current hearing technologies by the Food and Drug Administration (FDA) and with the existing service-delivery systems, advocating throughout the report for a range of technologies and service-delivery models, as well as proposing modification of FDA regulations to parallel those addressing devices for vision problems.

More recently, the National Academies of Sciences, Engineering, and Medicine (NASEM) published a report titled, *Hearing Health Care for Adults: Priorities for Improving Access and Affordability* (NASEM, 2016). In the introduction of this report, the following was noted:

The loss of hearing—be it gradual or acute, mild or severe, present since birth or acquired in older age—can have significant effects on one’s communication abilities, quality of life, social participation, and health. Despite this, many people with hearing loss do not seek or receive hearing health care. The reasons are numerous, complex, and often interconnected. For some, hearing health care is not affordable. For others, the appropriate services are difficult to access, or individuals do not know how or where to access them … The unmet need for hearing health care is high. Estimates of hearing aid use are that 67 to 86 percent of adults who may benefit from hearing aids do not use them … This study focuses on improving the accessibility and affordability of hearing health care for adults of all ages … Hearing health care is viewed through the social-ecological model that emphasizes the multiple levels of support and action needed throughout society to promote hearing and communication and reduce hearing loss and its effects. (pp. 1–2)

The report then proceeds to advocate for a wide range of technologies and service-delivery models to better meet the hearing-health-care needs of millions of older Americans with impaired hearing. The report recommends strongly that the FDA create a new category of technology, “over-the-counter wearable hearing devices,” with corresponding changes in the ways such devices would be delivered to consumers.

Clearly, the OTC service-delivery approach is designed to address affordability by bypassing the additional professional services often bundled with the price of the devices—that is, it is assumed that a hearing aid purchased from an audiologist using the best-practices model of service delivery (e.g., Kochkin et al., 2010; Valente et al., 2006) would be sold to the consumer at a higher price than the same device purchased via an OTC service-delivery model. Somewhat surprisingly, we could not find prior peer-reviewed publications on the impact of purchase price itself on measured hearing aid outcomes. Thus, even within a given service-delivery model, the influence of purchase price on outcome measures—especially self-report measures—is unknown. This may complicate the comparison of outcomes for typically higher priced advanced hearing aids delivered through an audiology-based best-practices service-delivery model and lower priced basic devices delivered through an OTC service-delivery model. Differences in outcomes across these two models could be due to actual differences in service-delivery processes, price, or both.

With the foregoing context in mind, this article describes in detail the first-ever placebo-controlled double-blind randomized clinical trial of hearing aid outcomes. Two service-delivery models were included in the clinical trial: an audiology-based best-practices (AB) model and an OTC consumer-decides (CD) model. The placebo (P) group received the same services as the participants in the AB group, but the hearing aids were programmed to be acoustically transparent (0-dB insertion gain). Importantly, by inclusion of the placebo group in this trial, the efficacy of *both* the best-practices AB model and the alternative OTC CD treatment can be established (e.g., International Conference on Harmonization, 1998). Thus, there were three parallel branches in this clinical trial, with participants randomly assigned to one of the branches following an eligibility screen.

The impact of the purchase price of the hearing aids on outcomes was also evaluated in this trial. The first half of the trial's participants paid $3,600 for their pair of hearing aids, which was considered a typical purchase price at study onset, whereas the subsequent half of participants paid $600 per pair, which was considered a reduced purchase price. Ideally, it would have been preferred to randomly assign the purchase price as well as the service-delivery model, but it was deemed too difficult to do so while maintaining the blinding of the participants and the audiologists with regard to purchase price.

In this report, we provide full procedural details and a detailed examination of the results, all based on per-protocol analyses. In addition, the online supplemental materials contain a full copy of the detailed clinical trial protocol (see Supplemental Material S1) and all data-collection forms (see Supplemental Material S2). For the complete SPSS raw data file, please contact the corresponding author.

## Method

### Overview of Design

This was a placebo-controlled double-blind randomized clinical trial with three parallel branches: audiology-based (AB) best-practices service delivery, consumer-decides (CD) or OTC service delivery, and placebo (P). The purchase price of the hearing aids provided to the participants was also manipulated: either a “typical” purchase price of U.S. $3,600 or a reduced purchase price of U.S. $600 for both hearing aids. As noted previously, this was done in a stratified manner with roughly the first half of study participants paying the typical price and the second half paying the reduced price.

### Hearing Aids

The study devices provided to all participants in the trial were ReSound Alera 9 mini-behind-the-ear (BTE) open-fit hearing aids having 17 log_10_-frequency-based (warp) compression bands with nine gain handles and four programs available. These mini-BTE open-fit devices are representative of the most popular type of hearing aid sold in the United States to older adults (e.g., Kochkin, 2011). All hearing aids were tested in the Verifit test box (Model VF-1; Software Version 3.10.9) to ensure that they met American National Standards Institute (ANSI, 2009) specifications prior to programming. The function of the directional microphone systems was also verified acoustically at that time. Default programming for all devices made use of fixed directional microphones, dynamic feedback suppression, and noise reduction. The four programs were configured to serve as a volume control for all study participants. For the AB and CD groups, the default program at start-up (Program 1) had a nominal output level of 0 dB volume-control setting for all hearing aids. Basically, at this setting, the output was set to match the NAL-NL2 (i.e., National Acoustic Laboratories’ Non-Linear Prescription Formula, Version 2) targets at each frequency. Programs 2 and 3 represented across-frequency decreases in this output in steps of X and 2X dB, respectively, whereas Program 4 was an increase in output of X dB, all relative to the output of Program 1 (0 dB). Thus, the programs simply raised and lowered the entire NAL-NL2 response up or down by various amounts as with a volume control. The value of X varied with the severity of high-frequency hearing loss (high-frequency pure-tone-average [HFPTA] based on hearing thresholds at 1000, 2000, and 4000 Hz) such that X = 4 dB for mild hearing loss (29 < HFPTA < 39 dB HL; ANSI, 2010) and X = 8 dB for more severe hearing loss (HFPTA ≥ 65 dB HL). These are just two examples of several variations in X. Further details can be found in the *ABCD Protocol Manual* (see online Supplemental Material S1). For the participants in the placebo group, their hearing aids programmed for 0-dB insertion gain, had minor gain variation across programs as follows: Program 1 = +2 dB; Program 2 = +1 dB; Program 3 = 0 dB; and Program 4 = +3 dB. At the 6-week follow-up (Session 3), hearing aid datalogging revealed that, on average, the hearing aids were in Program 1 74% of the time with no significant (*p* > .10) variations in this percentage across groups.

Some placebo devices made use of omnidirectional microphones (Po; *n* = 35) and others made use of the default directional microphones (Pd; *n* = 20). It was unclear whether the loss of some of the front–back localization ability due to a microphone location above the pinna (Chung, Neuman, & Higgins, 2008; Kuk, Korhonen, Lau, Keenan, & Norgaard, 2013) should also be restored for the placebo devices. This led to the exploration of the two different microphone options for the placebo group. In both cases, the frequency-gain characteristics were adjusted to achieve acoustic transparency—that is, flat insertion gain.

All markings and materials revealing manufacturer or model of the devices were obscured. Manufacturer and model were revealed to all participants only after the conclusion of the entire trial. There were a few exceptions, primarily involving the desire to purchase manufacturer compatible accessories, but in these cases the participant signed a confidentiality agreement regarding the identity of the manufacturer. All hearing-aid fits were bilateral.

### Participant Recruitment, Selection, and Randomization

Participants were recruited primarily by ads posted in the local newspapers and around the community. All testing of this volunteer sample took place in a university research clinic at Indiana University, Bloomington. Those interested in participating contacted the Clinical Trials Coordinator (CTC) by phone for an initial eligibility screen and then arranged an initial assessment to make a final determination of eligibility (Session 1). Inclusion criteria were as follows: (a) age 55–79 years; (b) English as native language; (c) Mini-Mental State Exam–2nd Edition (MMSE-2 SV; Folstein, Folstein, White, & Messer, 2010) score > 25; (d) no prior hearing aid experience; and (e) pure-tone air-conduction hearing thresholds consistent with age-related hearing loss, within the fitting guidelines of the study hearing aid, and bilaterally symmetrical. Exclusion criteria were as follows: (a) presence of a medically treatable ear condition; (b) bilateral, flat tympanograms; (c) known fluctuating or rapidly progressing hearing loss; (d) presence of cognitive, medical, or language-based conditions that limited ability to complete all test procedures; (e) currently taking or recently taken any platinum-based cancer drugs or mycin-family antibiotics; (f) previously diagnosed with either multiple sclerosis or Meniere's disease; (g) failure to seek or waive medical evaluation and clearance following hearing evaluation; and (h) unwillingness to be randomly assigned to a treatment group. Two individuals enrolled were younger than specified (53, 54 years) and one was older (83 years).

**Figure 1.**
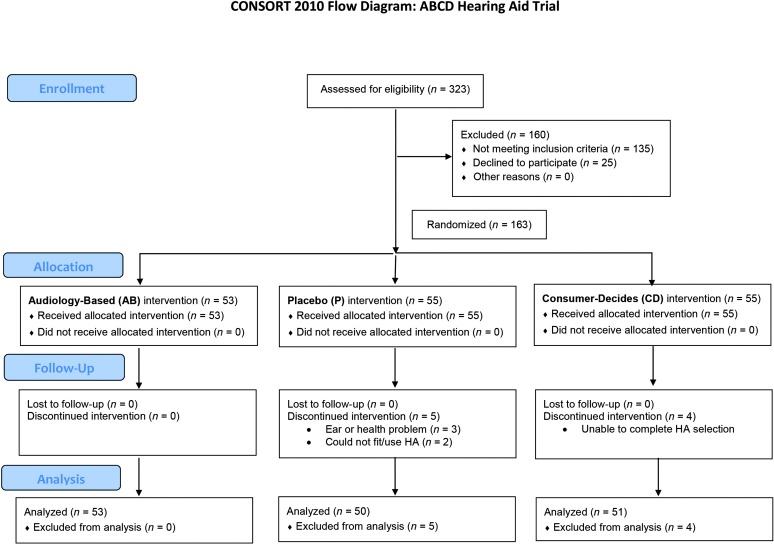
CONSORT flow diagram for the ABCD randomized clinical trial.

After each participant was consented for Session 1, measures completed were as follows: (a) otoscopic examination of both ears, (b) a complete audiological assessment, (c) MMSE-2 SV, and (d) a detailed case history. Following otoscopy, the audiological assessment began with 226-Hz tympanometry and automated ipsilateral pure-tone acoustic reflex threshold measurement at 500, 1000, and 2000 Hz using a Grason-Stadler Instruments (GSI) Model 39 immittance device. Air-conduction pure-tone audiometry followed, using a calibrated GSI-61 audiometer with Etymotic Research ER-3A insert earphones, at octave intervals from 250 Hz through 8000 Hz plus 1500, 3000, and 6000 Hz. Based on the foregoing inclusion and exclusion criteria, a preliminary determination of eligibility was made at this time. Those determined to be eligible completed the following measures in sequence: (a) speech reception threshold (SRT) for Central Institute for the Deaf (CID) W-1 spondaic words (Hirsh et al., 1952) using monitored live voice and 5-dB ascending step size; (b) word-recognition scores for recorded CID W-22 monosyllabic words (Department of Veterans Affairs, 2006) presented at 40 dB above SRT; (c) loudness discomfort level (LDL) measured with warble-tone stimuli centered at 500 Hz and 3000 Hz and using the instructions and 7-point scale from Cox, Alexander, Taylor, and Gray (1997); and (d) bone-conduction hearing thresholds at octave intervals from 250 Hz through 4000 Hz, plus 1500 Hz and 3000 Hz. After the audiological assessment, a sound-field measure of speech audiometry using a standard recording of the Connected Speech Test (CST; Cox, Alexander, & Gilmore, 1987) was completed using two passages (50 key words). This unaided sound-field CST score was used to block study participants prior to randomization (see below). Next, the MMSE-2 SV was completed, followed by a detailed case history (the full case history can be found in the *ABCD Protocol Manual*, see online Supplemental Material S1). A final determination of eligibility was then made and the Session 1 participant was counseled about the results (with a significant other present during the counseling, if desired). Acoustical real-ear-to-coupler differences (RECDs) were then measured for all participants determined to be eligible for the clinical trial. One of two audiologists, A1 or A2, was assigned to complete these Session 1 measures. As shown in the flow diagram in Figure 1, 323 individuals were assessed for eligibility. The trial commenced November 2, 2012, and data collection ended August 31, 2016, after enrollment targets based on preliminary statistical power calculations were met.

At completion of Session 1, eligible participants for the clinical trial (*n* = 188; see Figure 1) were escorted to the office of the CTC and were informed about the trial. Of the 188 eligible individuals, 163 enrolled in the trial, were consented for the trial, agreed to return for Session 2 (typically the next day), and were subsequently randomized to one of three groups: AB, CD, or P. Figure 1 presents additional information about the random allocation of participants and their completion of the trial. Of the 163 participants who enrolled, 98% self-reported their race as White and their ethnicity as not Hispanic or Latino, 78% had a college degree, and 80% reported annual household income greater than $45,000. Table 1 provides other demographic information for each group.

**Table 1.** Mean (*M*) and standard deviation (*SD*) for demographic measures for the audiology best practices (AB; *n* = 53; 28 males, 25 females), consumer decides/over-the-counter (CD; *n* = 55; 27 males, 28 females), and placebo device (P; *n* = 55; 37 males, 18 females) groups in this clinical trial.

Demographic	AB		CD		P		*F*(2, 160), *p*
	*M*	*SD*	*M*	*SD*	*M*	*SD*	
Age (years)	69.9	5.6	68.0	6.2	69.5	6.7	1.49, .23
MMSE-2 SV	28.9	1.1	28.7	1.2	28.7	1.3	0.75, .47
PTA (dB HL)	28.7	7.7	28.9	8.6	26.9	7.7	1.05, .35
HFPTA (dB HL)	39.2	7.8	38.9	7.9	38.4	8.2	0.14, .87
HHIE	27.7	13.6	29.3	17.3	29.0	16.4	0.15, .86

*Note.* The sample sizes are the original numbers at enrollment. Italics indicate significant (*p* < .05) analysis of variance (ANOVA) values, *F*(*df*). MMSE-2 SV = Mini-Mental State Exam–2nd Edition; PTA = pure-tone average; HFPTA = high-frequency pure-tone average; HHIE = Hearing Handicap Inventory for the Elderly.

Randomization was accomplished by generating columns of random numbers in a spreadsheet prior to study initiation. The a priori probability of assignment of a given participant to group AB, CD, or P was .33. In generating the randomization list for the study, iterations of the randomization were produced and evaluated until observed counts in each block of 100 randomizations were approximately 32–34 per group. For example, Participant 1 (P1), the first to enroll after screening, was designated by this randomization for assignment to group AB, the second participant (P2) to AB, P3 to CD, … P100 to P. Tallies of group assignments according to this randomization of the first 100 participants needed to be 32–34 participants for each of the three groups. If not, a new randomization list was generated. Once this was accomplished for the first set of 100 participants, a randomization list was then generated for a second and third set of 100 participants in similar fashion. Separate randomization lists were generated a priori for participants in each of the following unaided CST-performance categories: (a) CST ≤ 40%; (b) 40% < CST < 68%; and (c) CST ≥ 68%. The blocking of randomization by baseline (i.e., unaided) CST performance was necessary because several of the outcome measures included in the trial were relative measures of benefit. It is well known that the amount of benefit measured varies with the initial baseline performance. For example, those with high CST scores at unaided baseline will likely show smaller amounts of aided benefit, whereas those with lower baseline scores will likely show larger improvements when aided. By blocking the randomization based on CST baseline (i.e., unaided) performance prior to random assignment to groups AB, CD, or P, it was more likely that each group would include a similar number of low, medium, and high baseline CST scores and, therefore, would not bias the subsequent benefit measures derived for each group.

Only the CTC and the principal investigator (PI) had knowledge of and access to the randomization lists. The virtual lists were password-protected to prevent access by others on the research team. Once participants were assigned, those assignments were also secured digitally via password protection with hardcopy information secured via a locked file cabinet in the office of the CTC. The PI generated the random allocation sequences, and the CTC enrolled participants and assigned them to groups accordingly.

### Clinical Trial Baseline Measures and Interventions (Session 2)

**Figure 2.**
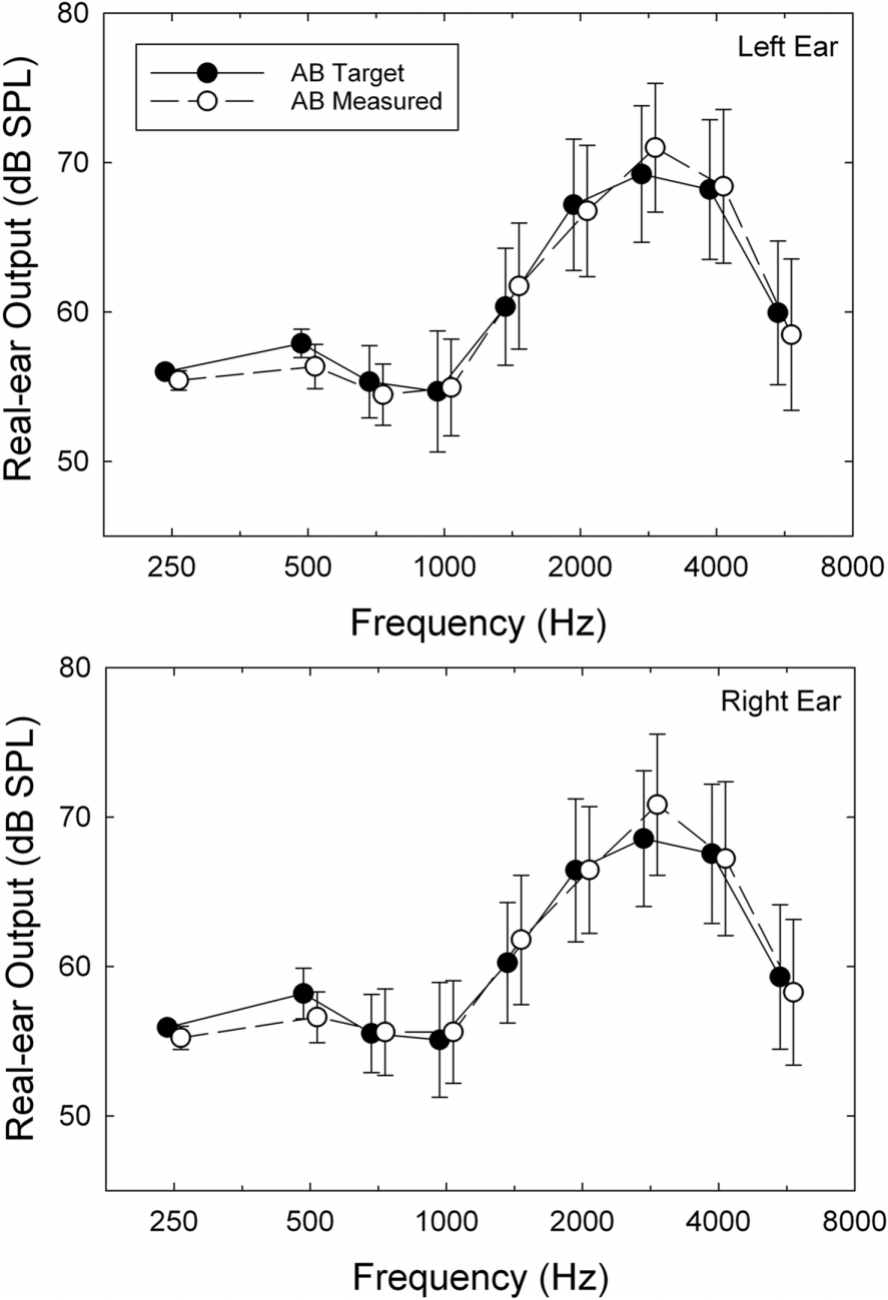
Comparison of prescribed (NAL-NL2) real-ear targets (filled circles) in dB SPL and measured (unfilled circles) real-ear levels for left (top) and right (bottom) ears for the audiology best practices (AB) group. The stimulus was a 65-dB SPL speech signal (“carrot passage”) for the speech-mapping measures from the Verifit test system. Symbol = mean value; error bar = ±1 standard deviation.

**Figure 3.**
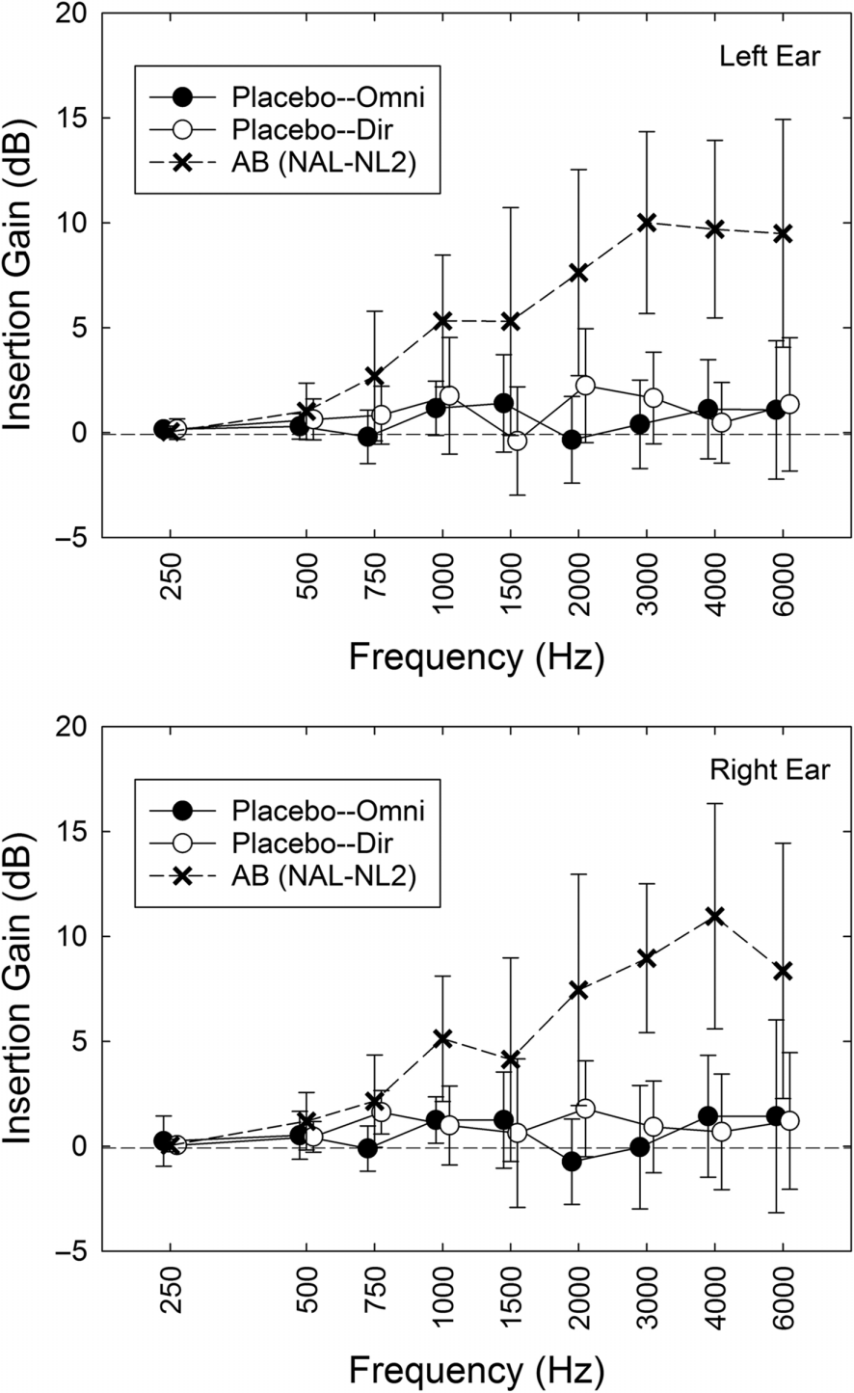
Means for real-ear insertion gain measured in the left (top) and right (bottom) ears of the placebo (P) group. Data are shown separately for each microphone-setting P subgroup: those with directional microphones (Placebo–Dir) and those with omnidirectional microphones (Placebo–Omni). The “target” gain for the P group is 0 dB. In addition, the mean measured real-ear insertion gain for the audiology best practices (AB) group is shown (X). All real-ear insertion gain values shown are for 65-dB-SPL pure-tone input stimuli. Error bars = ±1 standard deviation.

The procedures in Session 2 varied with group assignment. First, for all participants, unaided baseline scores were collected for two self-report outcome measures, the 66-item Profile of Hearing Aid Performance (PHAP; Cox & Gilmore, 1990) and the 25-item Hearing Handicap Inventory for the Elderly (HHIE; Ventry & Weinstein, 1982). Next, for groups AB and P, the procedures in Session 2 were nearly identical except that the hearing aids for the P participants were programmed to be acoustically transparent. First, the participant examined three colors of hearing aids available (gray, beige, brown) and selected the desired color. Audiologist A1 or A2, typically the same individual who worked with the participant in Session 1, selected the appropriate dome size (S, M, L, or tulip) and tubing length (0, 1, 2, or 3). ReSound's Aventa programming software (Version 3.6) was used to program the hearing aids. For those in group AB, the participant's audiograms were used to generate target gain prescriptions using the common NAL-NL2 (Dillon, Keidser, Ching, Flax, & Brewer, 2011) formula to compensate for the participant's hearing loss. (Using software obtained directly from the National Acoustics Laboratories [NAL], we verified that the targets generated in the Aventa software matched those intended for NAL-NL2 prescriptions. This is necessary because not all versions of “NAL-NL2” targets are actually equivalent to the desired targets; see, e.g., Sanders, Stoody, Weber, & Mueller, 2015.) For AB and the first 20 P participants, the default programming options were as follows: Fitting–experienced nonlinear; Binaural Correction–off; Directionality–fixed; Directional Mix–very low; Digital Feedback Suppression (DFS)–moderate; Expansion–off; Noise Tracker II–per environment; Wind Guard–off; Environmental Optimizer–0 dB; Tinnitus Sound Generator–off. For the subsequent 35 participants in group P, the microphone settings were changed from fixed to omnidirectional. For both AB and P participants, verification of the desired acoustic output of the hearing aids was accomplished via real-ear measurements using an Audioscan Verifit system (Model VF-1; Software Version 3.10.9). Figure 2 demonstrates good matches between prescribed and measured real-ear responses for speech input (“carrot passage” stimulus) for the AB group. Flat 0-dB insertion gain was also verified with real-ear measurements for the P group using pure-tone input stimuli, and Figure 3 demonstrates good matches to those targets. In both cases, the input level used for real-ear measures shown in Figures 2 and 3 was 65 dB SPL. Real-ear responses for input levels of 55 dB SPL and 75 dB SPL were also obtained for AB participants, but no fine-tuning of the response took place for these input levels. All real-ear measurements took place with the participant and the audiologist located in a sound-attenuating test booth meeting standards for ears-uncovered threshold testing (ANSI, 2013).

Next, for the AB participants only, pure-tone sweeps were run at 85 dB SPL to determine if any of these stimuli were uncomfortably loud using the prior LDL rating scale. The audiologist instructed the wearer to quickly raise his or her hand when an uncomfortably loud tone-burst (“beep”) was heard and observed the wearer while the sweep progressed from 250 Hz through 6000 Hz. Affirmative responses resulted in a 4-dB decrease in maximum output for that hearing aid at a frequency aligned with the affirmative response, and the 85-dB sweep was repeated. This process continued until the participant failed to raise his or her hand during a sweep. Once each hearing aid's maximum output had been set individually, both hearing aids were turned on and the 85-dB SPL sweep repeated for final verification of maximum output. Any indications of “uncomfortably loud” sound at this time resulted in a 2-dB reduction of maximum output of both hearing aids. For the 53 AB participants (106 hearing aids), no adjustments in maximum output were made in either ear for frequencies of 250 Hz, 500 Hz, or 750 Hz. The greatest number of adjustments occurred at 3000 Hz, with approximately 25% of the participants requiring adjustment. Of these, for 13 of the 24 hearing aids adjusted at 3000 Hz, adjustments were the smallest possible (4 dB), and only 5 of 24 exceeded two decrements (8 dB). Further adjustments in maximum output when the AB participant was wearing both hearing aids were very rare (5 out of a possible 477; 53 wearers × 9 frequencies). Matches to the 65-dB speech targets were not verified again after adjustments to maximum output were made following the 85-dB sweep. Given the low occurrence and small size of maximum output adjustments for the 85-dB stimulus summarized above, together with the nonlinear nature of the programmed output of the devices, it is unlikely that the responses for a 65-dB speech stimulus were impacted by these output adjustments. For the P participants, given the low gain of the devices, maximum output levels were established by entering 0 dB HL thresholds at all frequencies into the programming software (ReSound Aventa, Version 3.6) and using the first-fit option to generate the values for maximum power output. This resulted in maximum power output levels of 89, 94, 95, 98, and 100 dB SPL (re: HA-1 2-cm^3^ coupler) at octave intervals from 250 Hz through 4000 Hz and 94, 97, 99, and 100 dB SPL at octave intervals from 750 Hz through 6000 Hz. Based on the LDLs measured in each ear at 500 Hz and 3000 Hz, the default maximum power outputs noted above may have exceeded LDL for 10% or fewer of the P participants in one or both ears and at one or both frequencies.

This portion of Session 2 for both group AB and P participants concluded with a series of electroacoustic measurements performed on each hearing aid with an HA-1 2-cm^3^ coupler in the Verifit test box. The latter measures were used to facilitate comparisons to subsequent electroacoustic measurements when the participants returned 6 weeks later for Session 3.

Audiologist A1 or A2 did the hearing aid programming, real-ear verification, and any required fine-tuning to match targeted output. A third audiologist, A3, was blinded regarding group AB or P and was involved with the remainder of Session 2 for these two groups. This consisted of a 45–60-minute hearing aid orientation session during which Audiologist A3 reviewed the components, features, and function of the hearing aids; the insertion and removal of batteries; the insertion and removal of the hearing aids; maintenance practices; telephone use; and adjustment of volume. Supervised hands-on practice was provided for several of these skills. Participants were also counselled about benefits and limitations of hearing aids and effective communication strategies to use. A graduated usage schedule was also recommended for AB and P participants, beginning with relatively quiet listening conditions and at least 4 hours per day for the first week, progressively increasing the complexity of the listening conditions and the duration of usage (+2 hours/day each week). All participants (AB, CD, P) also received a written user guide for the hearing aids, which was modified from the manufacturer's guide to include much of this same information.

The use of probe-tube-microphone measurements to fine-tune the devices to the hearing aid wearer's hearing loss and the inclusion of hearing aid orientation information, including hands-on practice with these skills, are considered to be to critical aspects of audiology best practices (AB; Kochkin et al., 2010; Valente et al., 2006). Participants in both AB and P groups received these services. Participants in the CD group, however, did not receive real-ear adjustments of their hearing aids' response or a hearing aid orientation.

**Figure 4.**
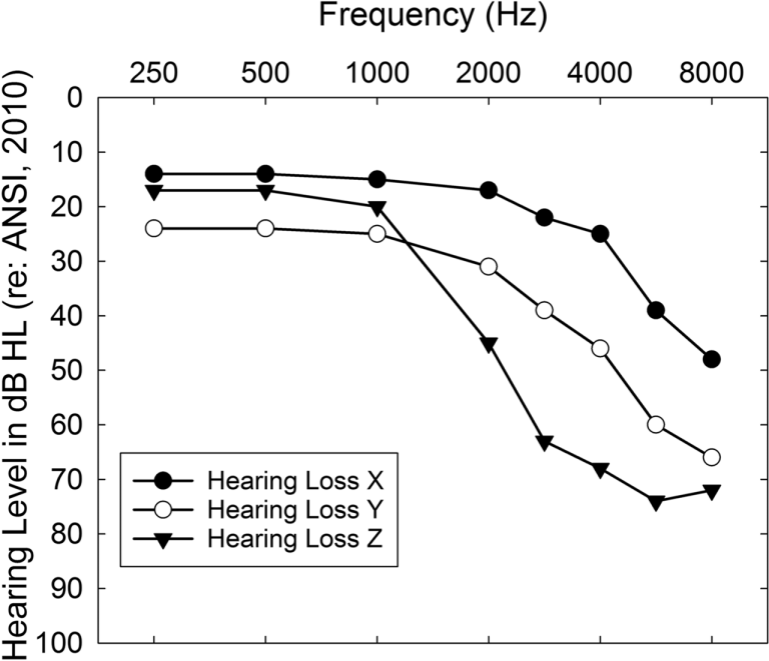
The X, Y, and Z audiograms from Ciletti & Flamme (2008), being among the most common audiometric configurations, were used to pre-match the hearing aids for the consumer decides/over-the-counter (CD) participants to NAL-NL2 targets.

For the CD participants, Session 2 commenced with the CTC escorting the participant to a small room. Containers for the ear tips/domes and tubing to be used—together with three bins containing six hearing aids each, with the hearing aids in each bin differing only in color (gray, beige, brown)—were located on a table in this room. Each bin was partitioned into three compartments; each compartment contained a pair of hearing aids labeled X, Y, or Z and programmed in advance to match the NAL-NL2 acoustic output prescriptions for the three most common patterns of hearing loss among older adults in the United States (Ciletti & Flamme, 2008). The three specific audiograms used for programming hearing aids X, Y, and Z are shown in Figure 4. The default programming options for these hearing aids were identical to those for the AB participants in regard to the various features of the hearing aids (microphone, feedback suppression, noise reduction, etc.). Maximum output levels for these devices were established by entering the pure-tone thresholds from the corresponding X, Y, or Z audiogram (see Figure 4) and using the first-fit option in the Aventa programming software to set the maximum power output levels. The resulting maximum output values are provided in Table 2.

**Table 2.** Maximum power outputs (MPOs) in dB SPL (re: HA-1 2-cm^3^ coupler) set using first-fit default settings in the *ReSound Aventa* programming software and NAL-NL2 for each of the audiograms depicted in Figure 4 (X, Y, and Z).

Frequency (Hz)	X	Y	Z
250	92	94	93
500	97	100	98
750	98	101	99
1000	99	102	100
1500	101	104	105
2000	102	106	110
3000	104	108	115
4000	105	110	118
6000	107	107	107

The participant watched a brief instructional video (see online Supplemental Material S3) overviewing the hearing aid self-selection process, then completed each step of the self-selection process. A hardcopy of the step-by-step instructions was also provided to the participant in the form of three binders, with the contents based on instructions provided for self-fitting hearing aids (Caposecco, Hickson, & Meyer, 2011). The CD participant first selected the desired hearing-aid color, examining several in the mirror; then selected an appropriately sized ear tip and tube; then the particular acoustic characteristics desired (X, Y, or Z). A tablet computer was available to provide standardized samples of speech, music, and environmental sounds for listening. Participants were allowed to have a significant other in the room with them during the selection process (only one CD participant opted to do so). As the participant tried the domes, tubes, and hearing aids, those in which he or she was not interested or did not fit properly were placed in a bin labeled “No.” When the participant's selections were finalized, the participant pressed a button to alert the CTC that the selections had been made. The CTC returned to the room, recorded these selections, and noted the contents of the other bin labeled “No.” Based on the tallies of items in the “No” bin, 65% of the CD participants had tried more than one dome, 59% tried more than one tubing size, and 90% tried more than one hearing aid. Typically, two to four hearing aids (out of six available) were in the “No” bin when the participant's selection process had been completed.

For all participants, Session 2 ended with a meeting with the CTC, during which the devices were delivered to the participant for use during the 6-week trial. Each participant was provided with the devices, tubes, and domes selected (as well as an extra set of domes); batteries; cleaning tools; and user guide. Payment was also collected at the end of this session. Participants either paid $3,600 with $300 (8.33%) credits for the completion of Sessions 2 and 3, or $600 with $50 (8.33%) credits for the completion of Sessions 2 and 3. The Session 2 credits were applied immediately upon completion of Session 2 and reduced the purchase price to $3,300 or $550. The final credit was disbursed as a check for either $300 or $50 at the completion of Session 3. The participants were not informed about the use of two different purchase prices until the final participant completed all testing. This deception was approved by the Indiana University Institutional Review Board and was necessary so as to assess the impact of purchase price on outcomes. After the final participant had completed the study, those paying the higher price received a letter from the PI and CTC informing them of the deception and a check for the difference in price ($2,500). Audiologists A1–A4 knew that two different purchase prices were being used in the trial, but they believed this was assigned randomly from participant to participant throughout the study, rather than blocked into two phases. The audiologists, moreover, were blind to the purchase price paid by each participant, as well as the blocking of payment in two phases during the trial, until completion of the entire trial.

If any of the participants had problems with their devices during the trial, they were asked to contact the CTC by phone. The CTC initially instructed the participant to review the user guide for assistance. If the participant did so and the problem persisted, and the problem was occurring for just one device, the CTC instructed the participant to remove both devices and compare them to see if anything differed between the two with regard to the tubing, dome, or battery. If the problem persisted, for either one or both hearing aids, the CTC made an appointment for an unscheduled visit. Unscheduled visits began with the CTC performing a visual inspection of the devices. If no problems were apparent, the CTC had the participant remove the devices and contacted Audiologist A1 or A2, whichever one had worked with this participant previously in Sessions 1 and 2. The audiologist first examined the hearing aids and, if nothing was found, next performed otoscopy, tympanometry, or other audiological measures to determine the nature of the problem and remedy it accordingly. The CTC received phone calls from a total of 31 participants between Sessions 2 and 3: 12 from AB participants, 10 from CD participants, and 9 from P participants. Of these, six AB, four CD, and six P participants came in for an unscheduled visit. Most complained of physical discomfort or irritation. Four of the six AB unscheduled visits, three of the four CD unscheduled visits, and two of the six P unscheduled visits resulted in changes to the domes or tubes. In addition, one of the two hearing aids were replaced for one AB participant (lost hearing aid) and one CD participant (broken battery door).

### Clinical Trial Post-Intervention Outcomes (Session 3)

During Session 3, typically 6 weeks after the initial fit of the hearing aids in Session 2 (*M* = 44.5 days; *SD* = 10.1 days), either Audiologist A1 or A2 (the one *not* involved in Session 2 programming) obtained as-worn aided CST scores and inspected the participant's ears, hearing aids, tubes, and domes, then obtained as-worn electroacoustic performance measures for each hearing aid. An inspection checklist was used to note any issues identified. Following completion of these as-worn measures, the hearing aids were reconditioned as needed (domes cleaned, kinked tubing replaced, dead or weak batteries replaced, etc.) and post-maintenance electroacoustic measures were obtained in the Verifit test box. Next, outcome measures were then obtained by another audiologist, A4. A4 was blind to participant group. The primary outcome measure for this trial was the Profile of Hearing Aid Benefit (PHAB). The PHAB score, a relative benefit measure, is the difference between the unaided and aided scores on the PHAP. The unaided PHAP was administered prior to (Session 2) and the aided PHAP following a 6-week period of hearing-aid use (Session 3), with the difference in ratings on the questionnaire used to compute the PHAB scores. Based on correlational and factor analyses of the seven PHAP subscales (unaided, aided, and difference or PHAB), the five communication-related subscales (Familiar Talkers, Ease of Communication, Reverberation, Reduced Cues, and Background Noise) were averaged to form a PHABglobal score, and the other two subscales (Aversiveness of Sound, Distorted Sound) were averaged to form a PHABavds score. The secondary outcome measure to be used in this clinical trial was derived from the unaided and aided CST speech-recognition scores. The secondary outcome measure was based on the difference in aided and unaided CST scores, CST benefit. The aided CST score was based on one passage pair or 50 key words and, like the unaided CST score, was obtained for a speech level of 65 dB SPL and a signal-to-babble ratio (SBR) of +3 dB. The speech level and SBR are similar to those used previously for the CST, including in a randomized clinical trial comparing hearing aids (Larson et al., 2000).

Because hearing-aid outcome in older adults has been demonstrated to be a multidimensional construct with three to four dimensions (Humes, 2001, 2003; Humes & Krull, 2012), some additional outcome measures were obtained from all participants. These included the self-reported benefit, measured as the difference between the unaided and aided HHIE scores; a 32-item survey of satisfaction with hearing-aid function (Humes, Garner, Wilson, & Barlow, 2001; Kochkin, 2000), referred to here as the Hearing Aid Satisfaction Survey (HASS); and the average daily usage of the hearing aid in hours/day, obtained from the data-logging feature of the hearing aids used in this trial. For the HASS, only 19 of the items dealing with hearing aid features and functions and the 13 pertaining to satisfaction with dispenser-related processes were included; the 10 additional items on satisfaction with performance in various listening situations were excluded from this trial due to redundancy with the PHAP. The items from each of these two subsections of the HASS were averaged (i.e., mean) to produce the HASShaf and HASSdisp satisfaction scores for the hearing-aid features/functions and the dispenser, respectively. For the measure of hearing aid usage, the total number of hours used from the data-logger was divided by the number of days between Sessions 2 and 3 to derive a measure of average hours used per day, or daily usage.

Next, all participants completed the Practical Hearing Aid Skills Test–Revised (PHAST-R; Doherty & Desjardins, 2012), modified slightly for open-fit BTE devices, to objectively assess the participant's ability to use and care for the hearing aids. A questionnaire about participation in musical training or auditory training during the trial period was also administered. Less than 2% of participants in the AB, CD, and P groups indicated participating in any auditory-based training exercises during the trial period, with no significant differences among the groups, χ^2^ (2) = 2.28, *p* > .05. With regard to musical experience or training during the trial, 16%–21% of each group reported singing or playing a musical instrument for at least 20 min twice per week, with no significant differences among the groups, χ^2^ (2) = 0.50, *p* > .05.

Each participant was then asked, “Now that you've worn these hearing aids for about 6 weeks, are you interested in keeping them?” Their responses were recorded as a preliminary indication of the likelihood that the hearing aids would be retained prior to revealing the group to which the participant had been assigned. Reasons offered in support of their responses were also noted on the response form by Audiologist A4.

Next, Audiologist A4 revealed the group to which each had been assigned. For the CD participants, regardless of their preliminary decision about keeping the hearing aids, each was offered another 4-to-5-week trial period with the AB service-delivery, including individual matching of gain and maximum output to the participant's hearing loss and subsequent verification, as well as a hearing-aid orientation session from an audiologist. P participants, given 0-dB insertion gain, were also offered another 4-to-5-week trial period for trial use of hearing aids matched to NAL-NL2 targets and maximum power outputs adjusted as needed. Because no adjustment of the hearing aids' programming took place between Sessions 2 and 3, the AB participants were also offered another 4-to-5-week trial following program adjustment to meet any complaints. Four of 53 AB participants, 49 of the 51 CD participants, and 47 of the 50 P participants who completed Session 3 opted for an additional 4-to-5-week trial at that time.

The procedures described for AB participants in Session 2 were followed for those in the CD and P groups participating in the additional 4-to-5-week trial, with the exception that those in the P group did not receive a second hearing-aid orientation session. After 4-to-5 weeks (*M* = 36.2 days, *SD* = 12.9 days), the procedures described previously for Session 3 were replicated. This is referred to here as Session 3B.

Those participants who completed Session 3 or Session 3B and did not wish to purchase the hearing aids at the conclusion of that session received a full refund of the purchase price and were exited from the study. Those who retained their hearing aids following Session 3 or Session 3B were scheduled for an annual follow-up and exited from the clinical trial.

## Results

### Examining Potential Participant-Selection Biases

As noted in Figure 1, of the 323 individuals who were screened and completed Session 1, 188 of these individuals met the clinical trial's eligibility criteria. Of these 188, 164 enrolled in the trial and 24 did not. It is appropriate to examine the characteristics of the participants (*N* = 164) and nonparticipants (*N* = 24) to see if the study sample may have been biased through this self-selection process.

Table 3 presents demographic and audiological data from the participants and nonparticipants. Independent-samples *t* tests were conducted for speech-recognition thresholds in the left and right ears, word-recognition scores in the left and right ears, LDLs at 500 Hz and 3000 Hz in the left and right ears, bilateral pure-tone averages (PTA; 500, 1000, and 2000 Hz), bilateral high-frequency pure-tone averages (HFPTA; 1000, 2000, and 4000 Hz), unaided CST scores, MMSE-2 SV scores, duration of hearing loss in years, and age. Nonparametric Mann–Whitney U and median tests were also conducted to examine differences in highest education level and annual household income, each of which was a closed-set ordinal measure in this study. Finally, a chi-square test was used to examine differences in gender between the participant and nonparticipant groups. No adjustments were made in the significance levels for multiple dependent variables. Of all the measures in Table 3, the participants differed significantly (*p* < .05) from the nonparticipants for only two measures: age and speech-recognition threshold in the left ear. On average, the nonparticipants were 3 years younger and had 5 dB lower speech-recognition thresholds than the participants. We conclude that the unwillingness of the nonparticipants to take part in the trial did not appreciably bias the study sample.

**Table 3.** Demographics for various measures from the 164 participants and the 24 nonparticipants.

Measure	Participants (*N* = 164)	Nonparticipants (*N* = 24)
Age in years, *M* (*SD*)	69.1 (6.1)	66.1 (6.6)*
Gender, % male	56.1	62.5
Median level of education	Master's degree	Bachelor's degree
Median annual household income	> $45,000	> $45,000
Duration of hearing loss in years, *M* (*SD*)	7.5 (8.4)	11.7 (14.0)
MMSE-2 SV, *M* (*SD*)	28.8 (1.2)	29.0 (1.1)
PTA bilateral in dB HL, *M* (*SD*)	28.1 (8.0)	26.7 (8.9)
HFPTA bilateral in dB HL, *M* (*SD*)	38.8 (7.9)	37.3 (9.6)
LDL 500 Hz right, dB HL, *M* (*SD*)	92.0 (11.8)	90.6 (12.1)
LDL 3000 Hz right in dB HL, *M* (*SD*)	95.4 (11.9)	96.2 (8.7)
LDL 500 Hz left dB HL, *M* (*SD*)	92.0 (12.2)	91.7 (13.4)
LDL 3000 Hz left in dB HL, *M* (*SD*)	96.7 (11.8)	99.6 (7.9)
SRT right in dB HL, *M* (*SD*)	22.4 (8.8)	19.8 (9.3)
SRT left in dB HL, *M* (*SD*)	22.8 (8.8)	17.9 (7.2)*
WRS-Q right (%), *M* (*SD*)	90.5 (8.2)	90.5 (8.3)
WRS-Q left (%), *M* (*SD*)	89.2 (8.5)	88.6 (7.9)
CST-unaided (%), *M* (*SD*)	53.0 (23.6)	49.2 (25.7)

*Note.* MMSE = Mini-Mental State Exam–2nd Edition; PTA = pure-tone average; HFPTA = high-frequency pure-tone average; LDL = loudness discomfort level; SRT = speech reception threshold; WRS-Q = word-recognition score in quiet; CST = Connected Speech Test.

* *p* < .05.

Also noted in Figure 1, of the 164 who enrolled in the clinical trial, one withdrew before returning for Session 2 and nine withdrew after completing Session 2 but prior to Session 3. Five of these nine had been assigned to group P and four to group CD. Reasons offered for withdrawal are noted in Figure 1. By completing Session 2, the nine who subsequently withdrew from the trial had all of the measures shown for the participants in Table 3, plus some additional baseline measures to be used for the outcome measures. In particular, measures of unaided PHAPglobal, PHAPavds, and HHIE were available for these nine participants. The same statistical analyses described previously for the analyses of participants and nonparticipants (independent-sample *t* tests, Mann–Whitney U and median tests, chi-square test) were conducted to compare the performance of the 154 participants who completed the trial to that of the nine participants who withdrew after completion of Session 2 for each of the demographic, audiological, and baseline outcome measures. Again, no corrections to the significance level were made for multiple dependent measures. No significant differences were observed, *p* > .05, between the performance of the 154 participants who completed Session 3 and those nine who withdrew after Session 2. We conclude that the withdrawal of these nine participants did not bias the study sample.

### Comparing the Two Placebo Subgroups

As noted previously, because we were uncertain how best to implement the placebo devices—with or without directional microphones—we included directional microphones in the first 18 placebo participants. After the next 18 (all of whom had omnidirectional microphones), a comparison was made between the two groups with regard to unaided baseline and aided measures for the primary and secondary outcome measures. No significant differences were observed for the PHAP subscales between these two versions of the placebo treatment, but there were marginally significant differences in aided CST scores with the directional microphone yielding superior performance. Given the configuration of the loudspeakers in the sound field for the target (0-degree azimuth) and competing (180-degree azimuth) stimuli for the CST, this observation was not entirely surprising. Nonetheless, because it was thought that the placebo treatment should not show improvements over unaided performance, especially for objectively measured speech recognition, the remainder of the P participants had hearing aids programmed with omnidirectional microphone function. In the end, there were 20 P participants who had hearing aids with directional microphones, two of whom withdrew between Sessions 2 and 3, and 35 P participants who had hearing aids with omnidirectional microphones, three of whom withdrew prior to study completion. The remaining 18 P participants wearing hearing aids with directional microphones were all in the typical purchase-price subgroup, and 8 of the 32 P participants wearing hearing aids equipped with omnidirectional microphones were in this same purchase-price subgroup (24 in the reduced purchase-price subgroup).

Table 4 compares the two P subgroups—those using hearing aids with directional microphones (Pd) and those using hearing aids with omnidirectional microphones (Po)—for several demographic, audiological, and outcome measures. As can be seen, there were no significant differences between these two P subgroups in terms of age, gender, level of education, annual household income, duration of hearing loss, or MMSE-2 SV, *p* > .05. There were also no significant differences in the average amount of hearing loss (PTA, HFPTA) or any of the unaided baselines for the outcome measures (PHAPglobal, PHAPavds, CST, HHIE), *p* > .05. Finally, of the primary (PHABglobal, PHABavds), secondary (CST benefit), and tertiary (HHIE benefit, HASS, and usage) outcome measures, the two P subgroups only differed significantly for the measure of CST benefit, *p* < .05, with the Pd subgroup demonstrating superior benefit. As noted in Table 4, all CST percent-correct scores were transformed into rationalized arcsine units (RAU; Studebaker, 1985) to stabilize the error variance prior to analyses. The superior performance of the Pd subgroup on the CST benefit measure results from a combination of this subgroup having worse unaided CST scores (45 RAU vs. 55 RAU for the Po subgroup), although not a statistically significant difference, *p* > .05, and better aided CST scores (64 RAU vs. 57 RAU for the Po subgroup), although again not a statistically significant difference, *p* > .05. The difference between aided and unaided CST scores, or CST benefit, shown in Table 4, *does* differ significantly between the two placebo subgroups, as noted above.

**Table 4.** Demographics and outcome measures for two placebo subgroups: those with omnidirectional microphones (Po) and those with directional microphones (Pd) in their hearing aids.

Measure	Po (*n* = 32)	Pd (*n* = 18)
Age in years, *M* (*SD*)	68.9 (6.8)	69.9 (5.4)
Gender, % male	68.7	66.7
Median level of education	Master's degree	Master's degree
Median annual household income	> $45,000	> $45,000
Duration of hearing loss in years, *M* (*SD*)	8.0 (6.4)	7.0 (7.8)
MMSE, *M* (*SD*)	28.5 (1.3)	28.9 (1.3)
PTA bilateral in dB HL, *M* (*SD*)	25.5 (8.3)	28.7 (6.9)
HFPTA bilateral in dB HL, *M* (*SD*)	37.1 (8.6)	40.5 (8.3)
Unaided PHAPglobal, *M* (*SD*)	0.40 (0.12)	0.41 (0.14)
Unaided PHAPavds, *M* (*SD*)	0.28 (0.12)	0.21 (0.09)
Unaided CST (RAU), *M* (*SD*)	55.2 (25.1)	45.1 (22.9)
Unaided HHIE, *M* (*SD*)	30.1 (15.2)	28.1 (15.7)
PHABglobal, *M* (*SD*)	0.03 (0.11)	0.05 (0.08)
PHABavds, *M* (*SD*)	0.02 (0.11)	0.03 (0.09)
CST benefit (RAU), *M* (*SD*)	2.8 (20.6)	19.1 (18.5)*
HHIE benefit, *M* (*SD*)	4.6 (11.9)	6.8 (11.0)
HASShaf, *M* (*SD*)	3.44 (0.67)	3.39 (0.38)
HASSdisp, *M* (*SD*)	4.54 (0.42)	4.49 (0.46)
Usage in hours/day, *M* (*SD*)	6.2 (3.3)	5.6 (3.6)

*Note.* MMSE = Mini-Mental State Exam–2nd Edition; PTA = pure-tone average; HFPTA = high-frequency pure-tone average; PHAPglobal = Profile of Hearing Aid Performance, average of the five communication-related subscales (Familiar Talkers, Ease of Communication, Reverberation, Reduced Cues, and Background Noise); PHAPavds = Profile of Hearing Aid Performance, average of the Aversiveness of Sound and Distorted Sound subscales; PHABglobal = difference between aided and unaided scores of PHAPglobal; PHABavds = difference between aided and unaided scores of PHAPavds; CST benefit = difference between aided and unaided Connected Speech Test scores; RAU = rationalized arcsine units; HHIE = Hearing Handicap Inventory for the Elderly; HHIE benefit = difference between aided and unaided HHIE scores; HASShaf = Hearing Aid Satisfaction Survey, items concerning hearing aid features; HASSdisp = Hearing Aid Satisfaction Survey, items concerning dispenser-related processes.

* *p* < .05.

Based on these analyses of the two P subgroups, the data for the Pd and Po subgroups will be pooled henceforth to represent the P group for all outcome measures. Specifically, for the examination of the factorial combination of the effects of service-delivery group (AB, CD, P) and purchase price (typical, reduced) on outcomes including CST benefit, the data from the Pd and Po subgroups are pooled to represent the entire P group. For CST benefit, this pooling of Pd and Po data increased the mean benefit by about 6 RAU regarding the benefit measured for the Po subgroup (8.7 RAU for combined P group vs. 2.8 RAU for Po alone).

### Hearing Loss

**Figure 5.**
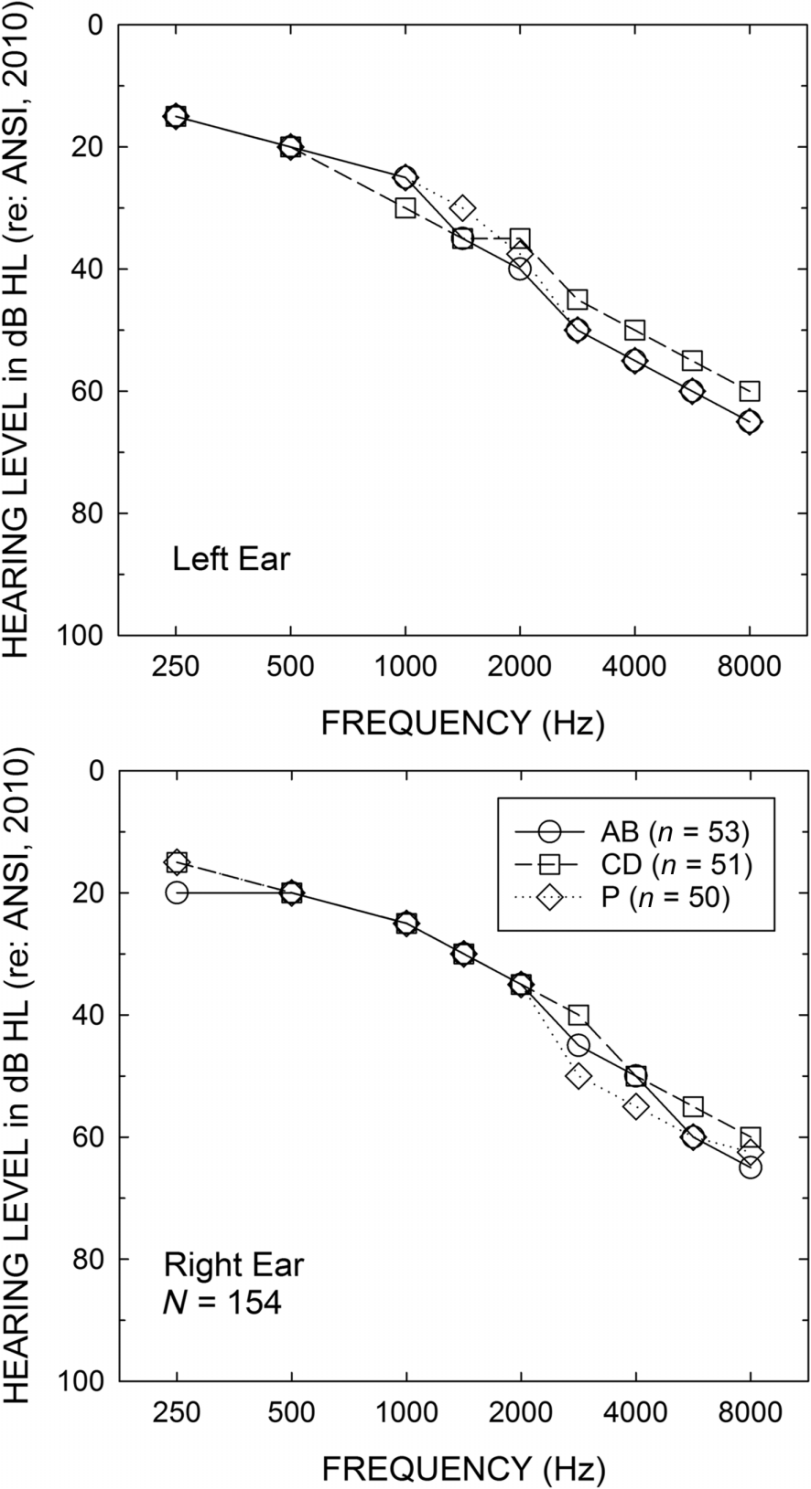
Median air-conduction pure-tone thresholds for left (top) and right (bottom) ears of the audiology best practices (AB), consumer decides/over-the-counter (CD), and placebo device (P) groups.

In the initial analyses of the results of this study, a series of general linear model (GLM) analyses were performed with between-subjects factors of service-delivery model (AB, CD, P) and price group (typical, reduced). We began first with evaluation of hearing loss, both PTA and HFPTA, using this 3 × 2 factorial analysis. There were no significant main effects of service-delivery group, both *F*(2, 148) values < 1.03, *p* > .05, or price group, both *F*(1, 148) values < 0.39, *p* > .05, and no significant interaction between these two factors for either PTA or HFPTA, both *F*(2, 148) values < 2.59, *p* > .05. Figure 5 shows the median air-conduction pure-tone thresholds for left (top) and right (bottom) ears of the AB, CD, and P groups. Because there were no main effects of price group or any interactions with this factor, the medians are for the pooled service-delivery groups. Clearly, there was little difference in pure-tone thresholds between ears and between groups.

### Outcome Measures

**Figure 6.**
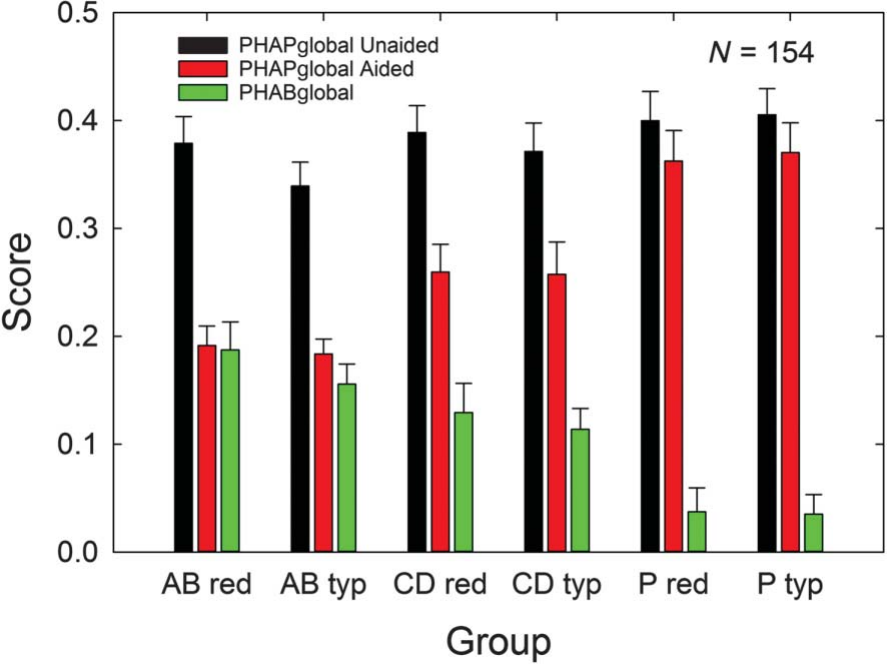
Means for the PHABglobal outcome measure for each of the six subgroups evaluated in the 3 × 2 factorial analysis. Unaided (black bars) and aided (red bars) PHAPglobal scores are provided, as well as the difference between these two scores, PHABglobal (green bars). Data are for *N* = 154, with 53 audiology best practices (AB), 51 consumer decides/over-the-counter (CD), and 50 placebo device (P) participants included. Purchase-price subgroups are “red” (reduced; $600) and “typ” (typical; $3,600). Error bars = +1 standard error; PHABglobal = difference between aided and unaided scores of PHAPglobal (Profile of Hearing Aid Performance, average of the five communication-related subscales: Familiar Talkers, Ease of Communication, Reverberation, Reduced Cues, and Background Noise).

Figure 6 shows the means and standard errors for the six subgroups evaluated in the 3 × 2 factorial analysis for the primary outcome measure in this clinical trial, PHABglobal. Figure 6 provides data for the unaided PHAPglobal (black bars), the aided PHAPglobal (red bars), and the difference between these two measures, the PHABglobal (green bars). Factorial (3 × 2) GLM analyses revealed that there were no significant main effects of price group, *F*(1, 148) values all < 0.82, *p* > .05, and no significant interactions with price group, *F*(2, 148) values all < 0.42, *p* > .05. The effect of service-delivery group was not significant for unaided PHAPglobal scores, *F*(2, 148) = 1.53, *p* > .05, but there were significant effects of service-delivery group on aided PHAPglobal, *F*(2, 148) = 27.37, *p* < .05, and PHABglobal, *F*(2, 148) = 18.56, *p* < .05. Post hoc Bonferroni-adjusted *t* tests revealed that group AB had significantly better (lower) aided PHAPglobal scores than both the CD and P groups, *p* < .05, and the CD group had significantly better aided PHAPglobal scores than the P group. With regard to the PHABglobal scores, the scores for the AB and CD groups were better than those of the P group, but the AB and CD groups did not differ significantly from one another, *p* > .05. In general, self-reported benefit for speech communication with amplification is improved for both AB and CD groups relative to the P group.

**Figure 7.**
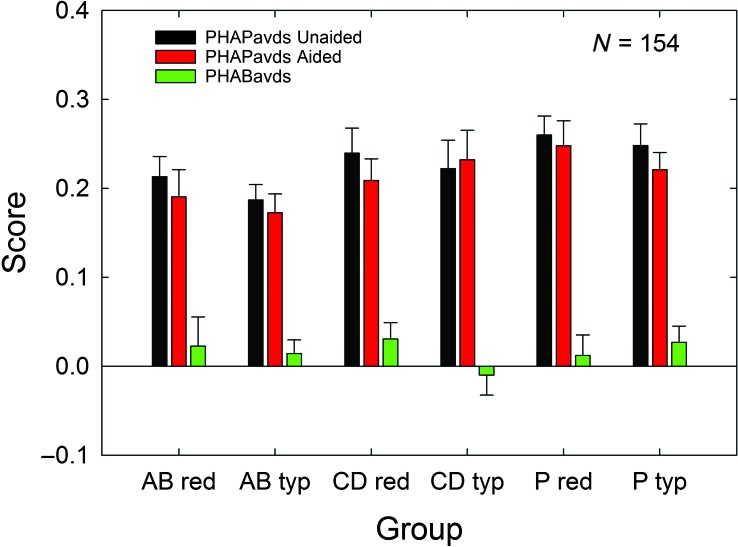
Means for the PHABavds outcome measure for each of the six subgroups evaluated in the 3 × 2 factorial analysis. Unaided (black bars) and aided (red bars) PHAPavds scores are provided, as well as the difference between these two scores, PHABavds (green bars). Data are for *N* = 154, with 53 audiology best practices (AB), 51 consumer decides/over-the-counter (CD), and 50 placebo device (P) participants included. Purchase-price subgroups are “red” (reduced; $600) and “typ” (typical; $3,600). Error bar = +1 standard error; PHABavds = difference between aided and unaided scores of PHAPavds (Profile of Hearing Aid Performance, average of Aversiveness of Sound and Distorted Sound subscales).

Figure 7 shows the means and standard errors for the unaided PHAPavds, aided PHAPavds, and PHABavds measures for each of the six participant subgroups. Factorial (3 × 2) GLM analyses of each of the three measures showed no significant effect of service-delivery group, all *F*(2, 148) values < 2.44, *p* > .05; no significant effect of price group, all *F*(1, 148) values < 0.85, *p* > .05; and no Service-Delivery Group × Price Group interaction for any of these three measures, all *F*(2, 148) values < 0.79, *p* > .05. Further, these analyses indicated that the PHABavds values (green bars)—pooled across all participants given the absence of any significant effects of service-delivery group, price group, and interaction—did not differ significantly from 0, *F*(1, 148) = 3.20, *p* > .05. The aversiveness and distortion of sound changes little from unaided to aided conditions, resulting in near-zero values for PHABavds.

**Figure 8.**
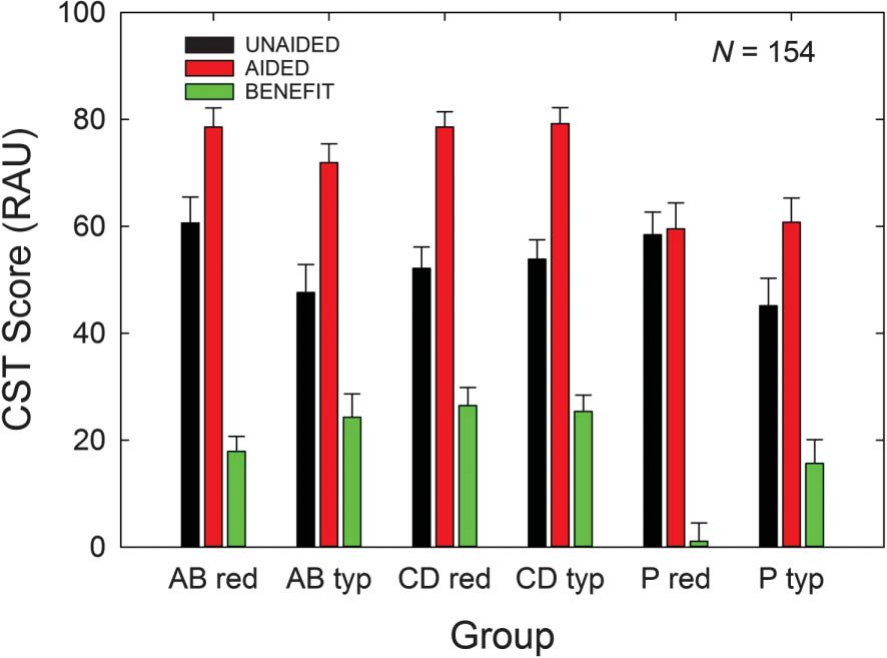
Means for the CST benefit outcome measure for each of the six subgroups evaluated in the 3 × 2 factorial analysis. Unaided (black bars) and aided (red bars) CST scores are provided, as well as the difference between these two scores, CST benefit (green bars). Data are for *N* = 154, with 53 audiology best practices (AB), 51 consumer decides/over-the-counter (CD), and 50 placebo device (P) participants included. Purchase-price subgroups are “red” (reduced; $600) and “typ” (typical; $3,600). All CST scores are shown in rationalized arcsine units (RAU). Error bars = +1 standard error; CST benefit = difference between aided and unaided Connected Speech Test scores.

Figure 8 depicts the means and standard errors for the secondary outcome measure in this clinical trial, the CST. All values in figures and used in GLM analyses made use of RAU-transformed CST scores. As in Figures 6 and 7, unaided (black), aided (red), and relative benefit (green) measures are provided. Factorial (3 × 2) GLM analyses revealed no significant interaction between the factors of service-delivery group and price group for any of the three measures shown in Figure 8, all *F*(2, 148) values < 2.19, *p* > .05. There was no effect of service-delivery group on unaided CST scores, but there was a significant effect on aided CST scores, *F*(2, 148) = 13.58, *p* < .05, and on CST benefit, *F*(2, 148) = 11.78, *p* < .05. Post hoc Bonferroni-adjusted *t* tests revealed that this significant effect was due to the P group performing significantly worse than both the AB and CD groups for the aided CST and CST benefit measures, *p* < .05. With regard to price group, there was a significant effect on unaided CST scores, *F*(1, 148) = 4.69, *p* < .05, and on CST benefit, *F*(1,148) = 4.79, *p* < .05, but not aided CST scores, *F*(1, 148) = 0.26, *p* > .05. Examination of Figure 8 shows that the reduced purchase-price subgroups (labeled on X axis as “red”) tend to have higher unaided CST scores and less CST benefit than their typical purchase-price counterparts (labeled as on X axis as “typ”). The impact of purchase price on CST benefit, the secondary outcome measure in this clinical trial, is clearly artefactual. There is no rational explanation for hearing-aid purchase price to impact *unaided* CST scores obtained *prior to* enrollment in the trial and subsequent purchase of the hearing aids. Given no differences in aided CST scores between these two purchase-price groups but significantly higher unaided scores for the reduced-price group, *CST benefit—*the difference between aided and unaided CST scores—is artificially lower in the reduced-price group. When the relative benefit measure, CST benefit, is confounded by seemingly arbitrary price-group differences in *unaided* CST scores, the measure of aided performance alone, aided CST, might be considered a more appropriate measure of the impact of amplification on speech understanding. Recall that there was no significant effect of purchase price on *aided* CST scores.

**Figure 9.**
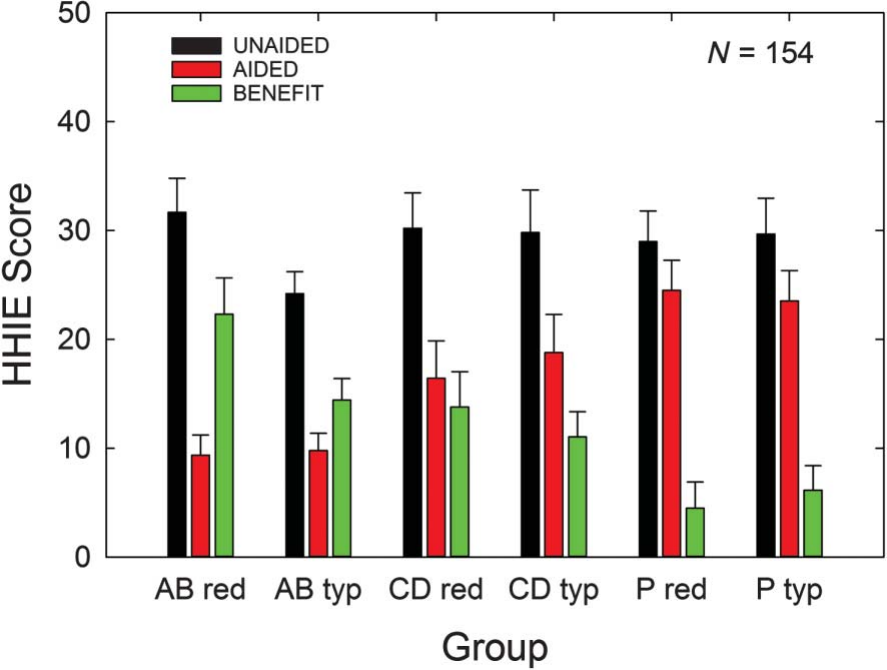
Means for the HHIE benefit outcome measure for each of the six subgroups evaluated in the 3 × 2 factorial analysis. Unaided (black bars) and aided (red bars) HHIE scores are provided, as well as the difference between these two scores, HHIE benefit (green bars). Data are for *N* = 154, with 53 audiology best practices (AB), 51 consumer decides/over-the-counter (CD), and 50 placebo device (P) participants included. Purchase-price subgroups are “red” (reduced; $600) and “typ” (typical; $3,600). All HHIE scores are total scores (emotional and social subscales combined). Error bar = +1 standard error; HHIE benefit = difference between aided and unaided Hearing Handicap Inventory for the Elderly scores.

Figure 9 shows the means and standard errors for unaided HHIE, aided HHIE, and HHIE benefit. There were no significant effects of price group, all *F*(1, 148) values < 1.90, *p* > .05, and no significant interactions between price group and service-delivery group, all *F*(2, 148) values < 1.63, *p* > .05. There was a significant effect of service-delivery group on aided HHIE, *F*(2, 148) = 13.90, *p* < .05, and on HHIE benefit, *F*(2, 148) = 12.09, *p* < .05, but not on unaided HHIE scores, *F*(2, 148) = 0.24, *p* > .05. Post hoc Bonferroni-adjusted *t* tests revealed that the AB group had significantly lower (better) aided HHIE scores than both of the other groups (CD, P), *p* < .05. No other post hoc comparisons were significant for aided HHIE. For HHIE benefit, the P group showed significantly less benefit than both of the other groups (AB, CD), *p* < .05, but the AB and CD groups did not differ significantly in terms of HHIE benefit.

**Figure 10.**
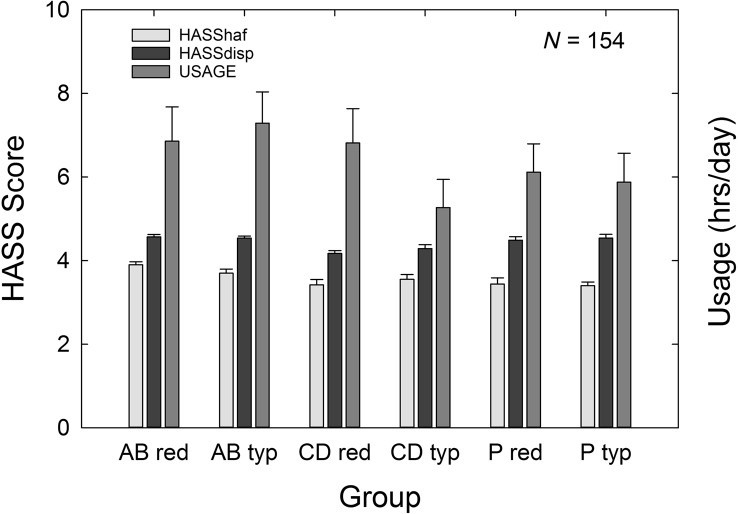
Means for the Hearing Aid Satisfaction Survey (HASS) satisfaction (left ordinate) and usage (right ordinate) outcome measures for each of the six subgroups evaluated in the 3 × 2 factorial analysis. Measures of satisfaction with hearing features and function, HASShaf (light gray bars); satisfaction with dispenser-related issues, HASSdisp (black bars); and daily hearing-aid usage from the datalogger (gray bars) are provided. Data are for *N* = 154, with 53 audiology best practices (AB), 51 consumer decides/over-the-counter (CD), and 50 placebo device (P) participants included. Purchase-price subgroups are “red” (reduced; $600) and “typ” (typical, $3,600). Error bars = +1 standard error.

Figure 10 shows the means and standard errors for the two measures of satisfaction, HASShaf and HASSdisp, and for average daily usage derived from the hearing aids' data-logging. All of these outcome measures are aided only; no unaided or “benefit” measures are possible. Data-logging values for the right and left hearing aids were strongly correlated, *r* = 0.96, *p* < .05. As a result, the mean of these two values was calculated and represents the average hours of use per day bilaterally. Factorial (3 × 2) GLM analyses on each of these three outcome measures revealed no significant main effect of price group, all *F*(1, 148) values < 0.72, *p* > .05, and no significant interactions, all *F*(2, 148) values < 0.58, *p* < .05, on any of the three outcome measures. However, a significant effect of service-delivery group was observed for both satisfaction measures, both *F*(2, 148) values > 6.92, *p* < .05, but not for daily usage, *F*(2, 148) = 1.33, *p* > .05. Post hoc Bonferroni-adjusted *t* tests revealed that the AB group had significantly higher HASShaf scores than both of the other groups (CD, P), *p* < .05, and that the CD group had significantly lower HASSdisp scores than the other two groups (AB, P).

#### Summary of GLM Analyses of Outcome Measures

Table 5 presents the group means and standard deviations for unaided baseline measures and aided outcome measures for each of the service-delivery groups: AB, CD, and P. No significant differences were observed for the unaided baselines, but five of the seven outcome measures show significant group effects. Only PHABavds and daily usage do not show significant differences across groups.

**Table 5.** Mean (*M*) and standard deviation (*SD*) for unaided baseline scores and outcome measures for the audiology best practices (AB; *n* = 53), consumer decides/over-the-counter (CD; *n* = 51), and placebo device (P; *n* = 50) groups in this clinical trial.

Measure	AB		CD		P		*F*(2, 148), *p*
	*M*	*SD*	*M*	*SD*	*M*	*SD*	
**Unaided baselines**							
HHIE	27.7	13.6	29.3	17.3	29.0	16.4	0.24, .79
CST (RAU)	53.8	27.1	51.8	20.5	53.4	24.7	0.13, .88
PHAPglobal	0.36	0.12	0.38	0.13	0.40	0.13	1.53, .22
PHAPavds	0.20	0.10	0.23	0.14	0.25	0.12	2.43, .09
**Outcome Measures**							
PHABglobal	0.17	0.12	0.12	0.12	0.04	0.10	***18.56, < .001***
PHABavds	0.02	0.13	0.01	0.10	0.02	0.10	0.10, .90
CST benefit (RAU)	21.3	19.4	26.6	16.1	8.7	21.2	***11.78, < .001***
HHIE benefit	18.2	14.2	12.3	14.1	5.5	11.6	***12.09, < .001***
HASShaf	3.80	0.45	3.48	0.62	3.42	0.58	***6.92, .001***
HASSdisp	4.56	0.28	4.23	0.40	4.52	0.43	***11.40, < .001***
Usage (hours/day)	7.09	3.95	6.12	3.93	5.99	3.39	1.33, .27

*Note.* These are per-protocol sample sizes based on number of participants who completed the clinical trial (*N* = 154). Bold values indicate significant (*p* < .05) general linear model (GLM) F values, *F*(*df*), for effects of service-delivery group from 3 × 2 factorial analyses. HHIE = Hearing Handicap Inventory for the Elderly; HHIE benefit = difference between aided and unaided HHIE scores; CST = Connected Speech Test; CST benefit = difference between aided and unaided Connected Speech Test scores; RAU = rationalized arcsine units; PHAPglobal = Profile of Hearing Aid Performance, average of the five communication-related subscales (Familiar Talkers, Ease of Communication, Reverberation, Reduced Cues, and Background Noise); PHAPavds = Profile of Hearing Aid Performance, average of the Aversiveness of Sound and Distorted Sound subscales; PHABglobal = difference between aided and unaided scores of PHAPglobal; PHABavds = difference between aided and unaided scores of PHAPavds; HASShaf = Hearing Aid Satisfaction Survey, items concerning hearing aid features; HASSdisp = Hearing Aid Satisfaction Survey, items concerning dispenser-related processes.

Table 6 presents a summary of the results from the GLM analyses for each outcome measure presented above. Here, the specific post hoc group differences underlying the significant effects of service-delivery group in the GLM analyses is noted. As can be seen, for the primary outcome measure (PHABglobal), the secondary outcome measure (CST benefit), and HHIE benefit, the pattern of results is very similar: no significant effects of purchase price, no significant interaction between purchase price and service-delivery model, and a significant main effect of service-delivery model with both AB and CD being significantly superior to P. The key exception to this general summary is that the effect of purchase price was significant for CST benefit, but as noted above, this appears to be artefactual to some extent due to significant effects of purchase price on *unaided* CST performance.

**Table 6.** Summary of significant main effects and interactions for general linear model (GLM) analyses of all hearing-aid outcome measures in the clinical trial.

Outcome measure	Service delivery (S)	Purchase price (PP)	S × PP
PHABglobal	AB > P, CD > P	NS	NS
PHABavds	NS	NS	NS
CST benefit	AB > P, CD > P	Typical > reduced^a^	NS
HHIE benefit	AB > P, CD > P	NS	NS
HASShaf	AB > CD, P	NS	NS
HASSdisp	AB, P > CD	NS	NS
Usage	NS	NS	NS

*Note.* AB = audiology best practices group; CD = consumer decides/over-the-counter group; P = placebo device group; NS = not significant (*p* > .05); PHABglobal = difference between aided and unaided scores of PHAPglobal (Profile of Hearing Aid Performance, average of the five communication-related subscales: Familiar Talkers, Ease of Communication, Reverberation, Reduced Cues, and Background Noise); PHABavds = difference between aided and unaided scores of PHAPavds (PHAPavds = Profile of Hearing Aid Performance, average of the Aversiveness of Sound and Distorted Sound subscales); CST benefit = difference between aided and unaided Connected Speech Test scores; HHIE benefit = difference between aided and unaided Hearing Handicap Inventory for the Elderly scores; HASShaf = Hearing Aid Satisfaction Survey, items concerning hearing aid features; HASSdisp = Hearing Aid Satisfaction Survey, items concerning dispenser-related processes.

a Typical > reduced also for unaided CST scores.

As summarized in Table 6, there were no significant main effects of service-delivery model or purchase price on PHABavds and no significant interaction between these two factors for this outcome measure. In fact, as noted above, the PHABavds scores overall did not differ significantly from zero, indicating that, overall, there were no effects of amplification on this outcome measure regardless of service-delivery or purchase-price group.

The remaining three outcome measures in Table 6 are aided-only measures: satisfaction (HASShaf, HASSdisp) or daily usage. There are no significant differences in usage attributable to service delivery or purchase price. For the two HASS measures, the CD group had significantly lower satisfaction ratings than the AB group for the hearing aids' features and function (HASShaf) as well as for the dispenser-related items (HASSdisp). The P group also reported significantly lower satisfaction scores than the AB group for the hearing aids' features and function, but not with regard to the dispenser.

#### Effect Sizes

The availability of data from the P group enabled the calculation of effect sizes for the two service-delivery models, AB and CD. Table 7 displays Cohen's *d* values for each service-delivery group relative to the P group and for each outcome measure included in this clinical trial. Cohen (1988) cautiously offered labels of “small,” “medium,” and “large” for *d* values of 0.2, 0.5, and 0.8, respectively. Using these labels as a loose guide, medium-to-large effect sizes were observed for *both* AB and CD service-delivery approaches for the primary outcome measure (PHABglobal), the secondary outcome measure (CST benefit), and HHIE benefit. This attests to the efficacy of both approaches to hearing-aid provision examined in this trial. On the other hand, the CD service-delivery model yielded considerably smaller effect sizes than the AB model for measures of satisfaction with the hearing aids' function and features and the daily usage of the devices. This was also true for satisfaction with dispenser-related performance (HASSdisp), but, by design, the CD group did not have interactions with a professional audiologist (minimal interactions with *any* member of the research team) and would be expected to be less satisfied with that aspect of the delivery process. Finally, as expected, the effect size for PHABavds was essentially zero and reflects the lack of benefit provided by hearing aids for aversive and distorted sounds regardless of the service-delivery model.

**Table 7.** Effect sizes (Cohen's *d*) for audiology best practices (AB) and consumer decides/over-the-counter (CD) service-delivery models relative to placebo device (P) for each of the outcome measures in this clinical trial.

Outcome measure	Cohen's *d*	
	AB vs. P	CD vs. P
PHABglobal	1.25	0.76
PHABavds	–0.02	–0.08
CST benefit	0.62	0.92
HHIE benefit	0.99	0.54
HASShaf	0.74	0.10
HASSdisp	0.39	–0.45
Usage	0.30	0.04

*Note.* PHABglobal = difference between aided and unaided scores of PHAPglobal (Profile of Hearing Aid Performance, average of the five communication-related subscales: Familiar Talkers, Ease of Communication, Reverberation, Reduced Cues, and Background Noise); PHABavds = difference between aided and unaided scores of PHAPavds (PHAPavds = Profile of Hearing Aid Performance, average of the Aversiveness of Sound and Distorted Sound subscales); CST benefit = difference between aided and unaided Connected Speech Test scores; HHIE benefit = difference between aided and unaided Hearing Handicap Inventory for the Elderly scores; HASShaf = Hearing Aid Satisfaction Survey, items concerning hearing aid features; HASSdisp = Hearing Aid Satisfaction Survey, items concerning dispenser-related processes.

### Other Findings From the 6-week Clinical Trial

At the end of the trial and prior to divulging the service-delivery approach or placebo status of the hearing aids, each participant who completed the trial (*N* = 154) was asked to indicate, based on their experience over the 6-week trial, whether they were likely to keep the hearing aids or return them for a refund. For the AB group, 81% indicated that they would keep the hearing aids, whereas only 55% of the CD group and 36% of the P group planned to keep them (the balance of the participants in each group were either not planning to keep the devices or were undecided). Chi-square testing revealed the AB percentage to be significantly greater than both CD, χ^2^ (2) = 8.3, *p* < .05, and P, χ^2^ (2) = 21.7, *p* < .05, percentages, but the difference between the CD and P groups was not significant, χ^2^ (2) = 3.8, *p* > .05. Of those who were not planning to keep their hearing aids or were undecided at that time, the most frequently cited reason for their response was the lack of benefit provided (1.9% of AB group, 17.6% of CD group, 38% of P group).

As noted, during Session 3, a slightly modified version of the PHAST-R was completed by all participants. This allowed for a formal assessment of their skills, such as inserting and removing the hearing aids, removing and inserting the battery, cleaning and maintaining the devices, and so on. Points were assigned for each skill by an audiologist (A4) blinded to study group. Points were totaled and divided by the maximum points possible, which could vary slightly from person to person, to yield a proportion of the maximum possible on the PHAST-R. The means (standard deviations) were 0.90 (0.09), 0.84 (0.09), and 0.89 (0.07) for the AB, CD, and P groups, respectively. Analysis of variance revealed a significant effect of service-delivery group on PHAST-R proportions, *F*(2, 151) = 8.39, *p* < .05, and follow-up Bonferroni-adjusted *t* tests showed that the CD group had significantly lower PHAST-R proportions than the other two groups, *p* < .05, with no other significant paired comparisons emerging. Given the lack of hearing-aid orientation during Session 2 for the CD participants, the poorer performance of the CD group on the PHAST-R is not unexpected.

When participants returned for Session 3, the blinded audiologist (A1 or A2) first inspected the hearing aids for proper insertion, examined the tubing and the domes, and removed the hearing aid battery and measured its voltage. The number of times a problem occurred was noted for each hearing aid during these Session 3 inspections. Tallies were compiled for the number of inspection problems and then compared across groups. Hearing-aid insertion problems were noted about 36% of the time on one or both sides, but there were no differences among the three groups in the frequency of these problems, χ^2^(4) = 4.63, *p* > .05. Tubing problems, especially twisted or kinked tubing, were the most commonly observed problem, occurring on one or both ears 88% of the time, but there were no differences among groups regarding the frequency of observed tubing problems, χ^2^(8) = 6.63, *p* > .05. Weak or dead batteries, on the other hand, were the least frequent problem, occurring 17.5% of the time, with no significant differences among groups in the frequency of these problems, χ^2^(4) = 1.47, *p* > .05.

Given the high prevalence of various hearing-aid problems, we sought to determine the functional significance of these issues. We had obtained as-worn acoustical measures of each hearing aid in the Verifit test box at the beginning of Session 3 and also had subsequent postmaintenance measures of the same type for each hearing aid. For these acoustical measurements, several gain and output measures were available. Here, we opted to use the aided Speech Intelligibility Index (SII; ANSI, 2012) from the Verifit device as a global single-valued estimator of the acoustical quality of fit. In general, over a fairly wide range of SII values, speech-understanding performance is monotonically related to the SII. Values from each ear were available for input levels of 55, 65, and 75 dB SPL and, based on the strong significant correlations among the SII values for different levels within the same ear, *r* > 0.70, *p* < .01, we opted for the SII values at 65 dB SPL for each hearing aid as our global indicator of acoustical function. In addition, CST scores were obtained in Session 3 for both the initial as-worn condition of the hearing aids and also after maintenance and adjustment of the hearing aids by the audiologist. These RAU-transformed scores served as a single-valued metric for functional consequences of these hearing aid maintenance issues. Table 8 shows the means and standard deviations for these acoustical and functional measures as-worn and postmaintenance for each group. For each of the SII values—one for each hearing aid—and for the sound-field (binaural) CST scores, mixed-model 3 × 2 factorial GLM analyses were performed to examine the between-subjects effects of service-delivery group (AB, CD, P) and repeated-measures effect of hearing-aid maintenance (as-worn, postmaintenance). As expected, for each of the three GLM analyses, a significant effect of group was observed with post hoc testing revealing that the P group had significantly lower SII values and aided CST scores, *p* < .05, than each of the other groups (AB, CD), all *F*(2, 151) values > 7.22, *p* < .05. The AB and CD groups did not differ significantly from one another, *p* > .05, nor was the interaction between group and hearing-aid maintenance significant in any of the three GLM analyses, *F*(2, 151) values < 1.42, *p* > .05. In all three GLM analyses, SII values and CST scores obtained postmaintenance were significantly greater than those measured as-worn, *F*(1, 151) values > 18.6, *p* < .05. Thus, the hearing-aid maintenance problems observed at the end of the 6-week trial were not inconsequential; rather, they resulted in significantly decreased amplification of speech (SII) and significantly poorer aided speech-understanding performance (CST).

**Table 8.** Mean (standard deviation) of the Speech Intelligibility Index (SII) values, in percentage points, obtained in the Verifit test box for 65 dB SPL input for each hearing aid, as-worn (AW) and post-maintenance (PM), and rationalized arcsine units–transformed Connected Speech Test (CST) scores at 65 dB SPL, AW and PM, for each of the service-delivery groups.

Group	SII-AW-R	SII-PM-R	SII-AW-L	SII-PM-L	CST-AW	CST-PM
AB	65.5 (15.4)	69.5 (10.3)	64.9 (11.4)	67.7 (9.4)	66.5 (21.6)	75.1 (18.4)
CD	60.9 (21.5)	66.3 (16.4)	58.5 (19.8)	64.2 (15.6)	68.7 (17.4)	78.9 (14.6)
P	54.4 (18.1)	57.9 (15.7)	52.5 (17.2)	55.5 (15.5)	49.5 (25.2)	60.2 (23.3)

*Note.* R = right; L = left; AB = audiology best practices group; CD = consumer decides/over-the-counter group; P = placebo device group.

We thought that the CD group, lacking exposure to hearing-aid orientation with an audiologist, might show a greater dependence on use of the user guide or find it to be more helpful than the other two groups who had received such instruction (AB, P). Approximately 90% of the participants in each group (86.7%–94.1%) indicated that they read the user guide that was sent home with the hearing aids. There was no significant difference in the proportion of each group who did so, χ^2^(4) = 3.87, *p* > .05. Further, when those who read it were asked to rate the helpfulness of the user guide on a scale of 1 to 10, with 10 = *most helpful,* the mean (standard deviation) ratings were 7.6 (2.1), 7.0 (2.1), and 7.0 (2.3) for the AB, CD and P groups, respectively. There were no significant differences in these user guide ratings among groups, *F*(2, 136) = 0.90, *p* > .05. Overall, all groups tended to use the guides and considered the user guides to be helpful, similarly so across groups.

### Outcomes from the Follow-Up Trial (Session 3B)

Recall that at the conclusion of the 6-week clinical trial, all 154 participants in Session 3 were informed of their group assignment and offered the opportunity to continue for another 4-week trial period after adjustments to their hearing aids. For the 53 AB participants, five had decided to return their hearing aids at the end of the 6-week trial, four requested adjustments and another 4-week trial period, and the rest purchased their hearing aids. Given the small number of AB participants continuing for another 4-week trial period, these individuals were omitted from any comparisons among outcome measures across Sessions 3 and 3B. For the 51 CD participants, one kept the hearing aids selected for the trial and one returned the hearing aids in Session 3, with the remaining 49 CD participants requesting the additional 4-week trial period before making a final decision. For the P participants, three returned their hearing aids at the conclusion of Session 3 and 47 requested the additional 4-week trial period.

One CD participant and one P participant withdrew from the extended trial period prior to completing the measures in Session 3B, but both opted to purchase their devices after adjustment. All 94 remaining participants (48 CD, 46 P) received the AB service-delivery model, including real-ear measurements to verify NAL-NL2 targets and individual adjustment of maximum power output. Only the CD participants also received a hearing-aid orientation at this time because the P participants received hearing-aid orientation during the original 6-week trial period.

**Figure 11.**
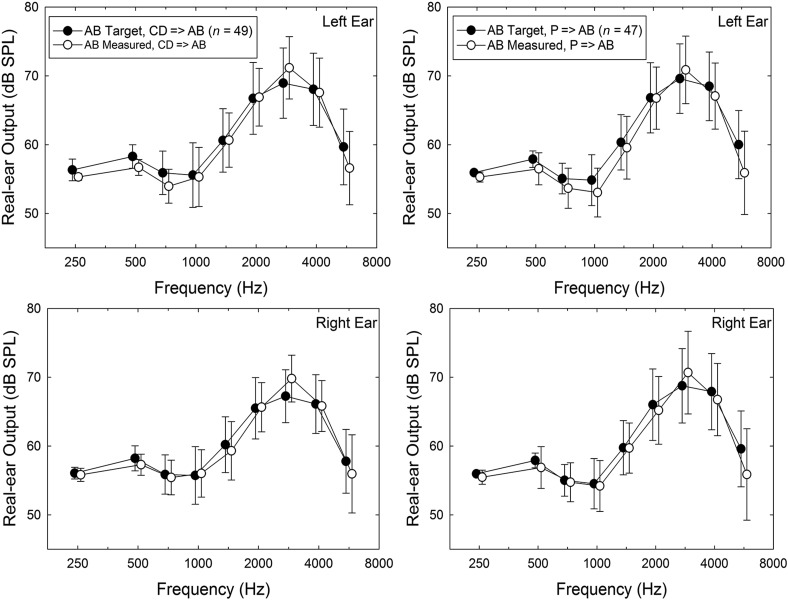
Comparison of real-ear output to NAL-NL2 targets after hearing aids for consumer decides/over-the-counter (CD; left) and placebo device (P; right) participants were reprogrammed to match targets following audiology best practices (AB) procedures in Session 3. Mean and ±1 standard deviation are shown in each panel. Top panels show data for the left ear and bottom panels for the right ear. The stimulus was a 65-dB SPL speech signal (“carrot passage”) for the speech-mapping measures from the Verifit test system.

Figure 11 provides the aided output (unfilled circles) for a 65-dB SPL speech input for each hearing aid when reprogrammed for the original CD and P participants after matching NAL-NL2 targets at the end of Session 3. Target output values (filled circles) are also shown in each panel.

**Figure 12.**
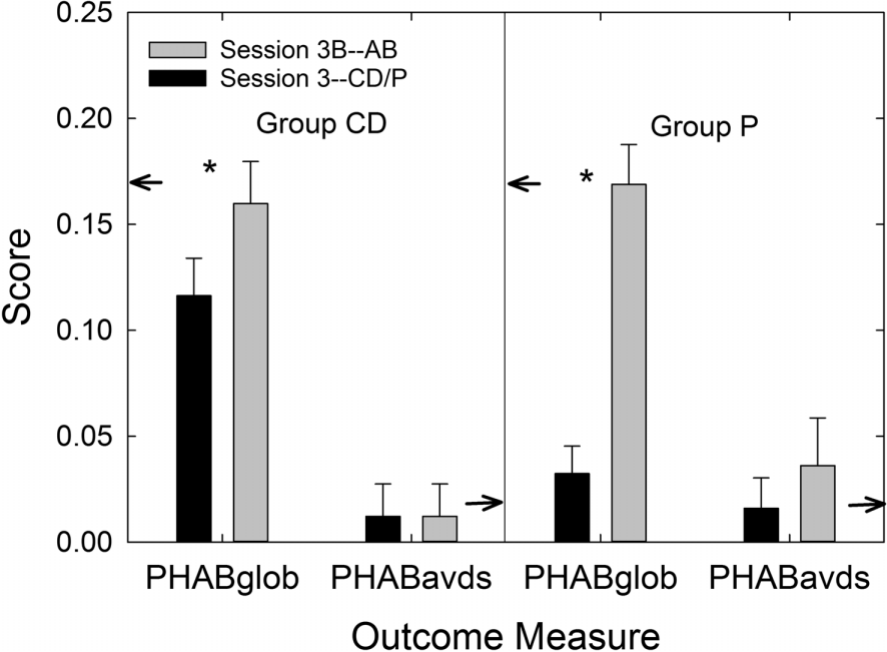
Mean PHABglobal and PHABavds scores obtained from the consumer decides/over-the-counter (CD) group (left panel; *n* = 48) and the placebo device (P) group (right panel; *n* = 46) during Sessions 3 (black bars) and Session 3B (gray bars). The left-pointing arrows show the mean values for the original audiology best practices (AB) group from Session 3 for the lefthand outcome measure in each panel, and the right-pointing arrows show the corresponding Session 3 AB values for the righthand outcome measure in each panel. Error bar = +1 standard error; PHABglob = difference between aided and unaided scores of PHAPglobal (Profile of Hearing Aid Performance, average of the five communication-related subscales: Familiar Talkers, Ease of Communication, Reverberation, Reduced Cues, and Background Noise); PHABavds = difference between aided and unaided scores of PHAPavds (PHAPavds = Profile of Hearing Aid Performance, average of the Aversiveness of Sound and Distorted Sound subscales). *Significant (*p* < .05) paired-sample *t* tests in each panel.

Figure 12 shows the mean (+1 standard error) PHABglobal and PHABavds scores obtained from the CD group (left panel) and the P group (right panel) at Session 3 (black bars) and after the additional 4-week trial in Session 3B (gray bars). Asterisks (*) mark significant (*p* < .05) paired-sample *t* tests in each panel. The PHABglobal scores improved significantly for both the CD and P participants after AB service delivery and the extra 4-week trial period. No significant differences were observed for the PHABavds scores. The left-pointing arrows show the mean values for the original AB group from Session 3 for the lefthand outcome measure in each panel, and the right-pointing arrows show the corresponding Session 3 AB values for the righthand outcome measure in each panel. As can be seen, the mean values for the PHABglobal and PHABavds outcome measures obtained in Session 3B from the original CD and P participants following an additional 4-week AB-based trial (gray bars) are very similar to those obtained from the original AB participants (arrows).

**Figure 13.**
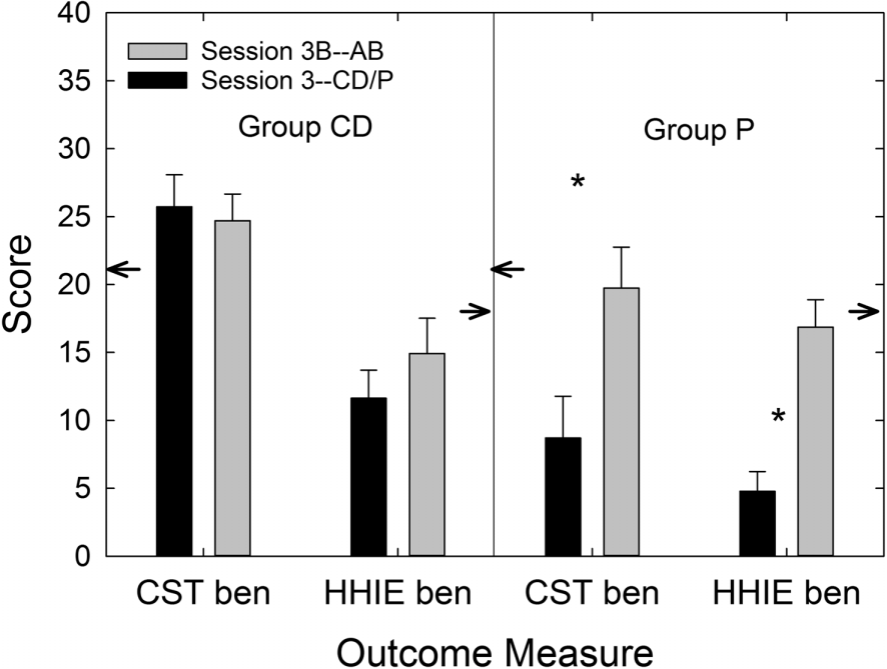
Mean CST benefit, in rationalized arcsine units (RAU), and Hearing Handicap Inventory for the Elderly benefit (HHIE ben) scores obtained from the consumer decides/over-the-counter (CD) group (left panel) and the placebo device (P) group (right panel) during Sessions 3 (black bars) and Session 3B (gray bars). The left-pointing arrows show the mean values for the original audiology best practices (AB) group from Session 3 for the lefthand outcome measure in each panel, and the right-pointing arrows show the corresponding Session 3 AB values for the righthand outcome measure in each panel. Error bar = +1 standard error; CST ben = difference between aided and unaided Connected Speech Test scores. *Significant (*p* < .05) paired-sample *t* tests in each panel.

Figure 13 provides the mean (+1 standard error) CST benefit and HHIE benefit measures for the participants assigned to the CD and P groups for the initial trial period. Whereas the CD group shows no significant improvement from Session 3 to Session 3B for either CST or HHIE benefit, the P group shows significant improvements on both outcome measures. The mean values for CST and HHIE benefit obtained in Session 3B from the original CD and P participants following an additional 4-week AB-based trial (gray bars) are very similar to those obtained from the original AB participants (arrows).

**Figure 14.**
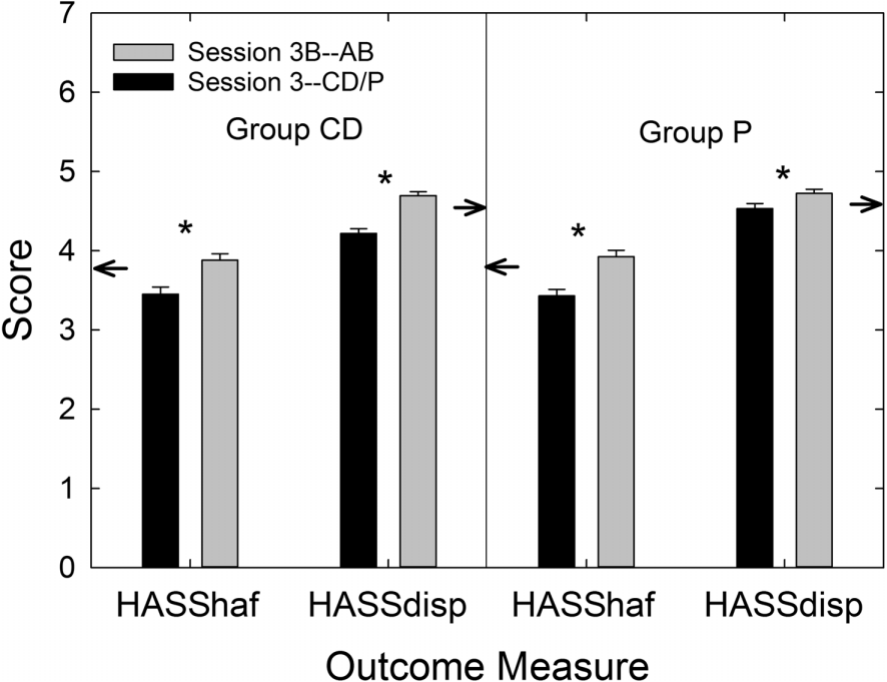
Mean HASShaf and HASSdisp scores obtained from the consumer decides/over-the-counter (CD) group (left panel) and the placebo device (P) group (right panel) during Sessions 3 (black bars) and Session 3B (gray bars). The left-pointing arrows show the mean values for the original audiology best practices (AB) group from Session 3 for the lefthand outcome measure in each panel and the right-pointing arrows show the corresponding Session 3 AB values for the righthand outcome measure in each panel. Error bar = +1 standard error; HASShaf = Hearing Aid Satisfaction Survey, items concerning hearing aid features; HASSdisp = Hearing Aid Satisfaction Survey, items concerning dispenser-related processes. *Significant (*p* < .05) paired-sample *t* tests in each panel.

Figure 14 provides a similar comparison of the mean (+1 standard error) outcomes from Session 3 to Session 3B for the CD and P participants for the two measures of hearing-aid satisfaction, HASShaf and HASSdisp. For both the CD and P groups, the AB service-delivery model and 4-week trial led to significantly higher satisfaction ratings both about the features and function of the hearing aids (HASShaf) and the dispenser-related items (HASSdisp). The mean values for both HASS outcome measures obtained in Session 3B from the original CD and P participants following an additional 4-week AB-based trial (gray bars) are very similar to those obtained from the original AB participants (arrows).

**Figure 15.**
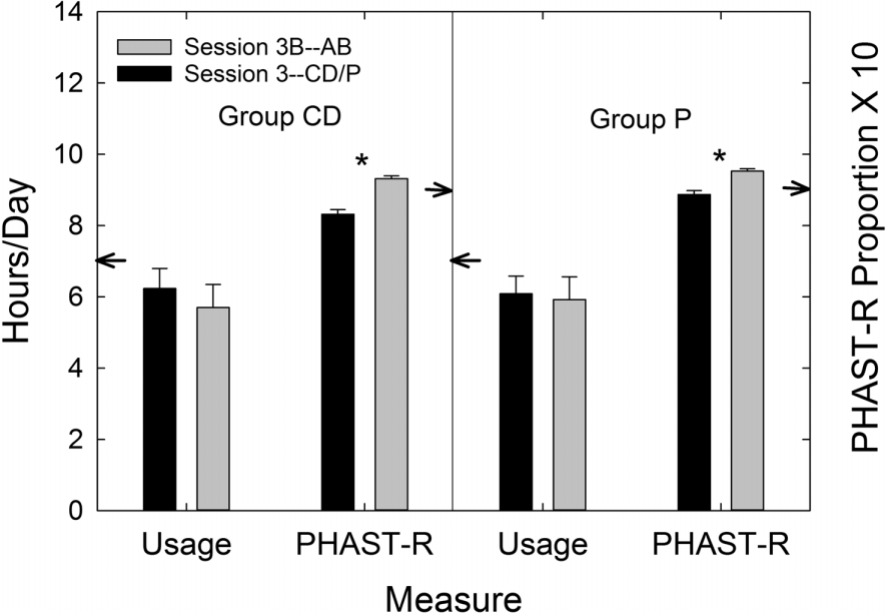
Mean usage outcome (left ordinate) and Practical Hearing Aid Skills Test–Revised (PHAST-R) scores (right ordinate) obtained from the consumer decides over-the-counter (CD) group (left panel) and the placebo device (P) group (right panel) during Sessions 3 (black bars) and Session 3B (gray bars). Note that the PHAST-R proportions have a maximum of 1.0 but are multiplied by 10 for appropriate scaling in this figure. The left-pointing arrows show the mean values for the original audiology best practices (AB) group from Session 3 for the lefthand outcome measure in each panel and the right-pointing arrows show the corresponding Session 3 AB values for the righthand outcome measure in each panel. Error bar = +1 standard error. *Significant (*p* < .05) paired-sample *t* tests in each panel.

Figure 15 shows the mean (+1 standard error) for the hearing-aid usage outcome measure derived from data-logger readings obtained from each group and from Session 3 and Session 3B. The usage measures show no significant change from Session 3 to Session 3B for either group and average about 6 hours per day across groups and sessions. The mean values for hours of daily usage from the original CD and P participants are similar to the means of the original AB participants (arrows), although averaging about 1 hour less per day than the original AB group.

Also shown in Figure 15 are the mean (+1 standard error) PHAST-R scores obtained from each group at both Session 3 and Session 3B. For both groups, the PHAST-R scores increased significantly from Session 3 to Session 3B. Once again, the mean values for the PHAST-R obtained in Session 3B from the original CD and P participants following an additional 4-week AB-based trial (gray bars) are very similar to those obtained from the original AB participants (arrows).

**Figure 16.**
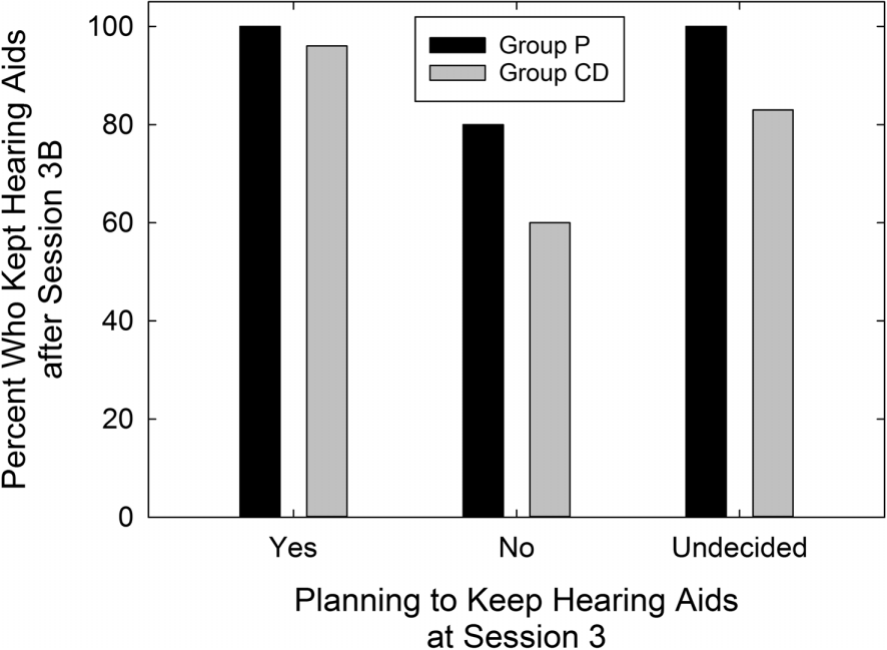
Percentage of participants, assigned at the start of the trial to placebo device (P) and consumer decides/over-the-counter (CD) group and who participated in the extra 4-week trial with audiology best practices (AB) service delivery, who ultimately kept their hearing aids after Session 3B. This is plotted against these same participants' plans to keep the hearing aids when asked at the conclusion of the initial 6-week trial and prior to revealing their group.

Finally, as was noted, all but a few of the original CD and P group members opted to receive the AB service-delivery and an additional 4-week trial. Recall that at the end of the clinical trial, Session 3, and prior to divulging group membership, each participant indicated whether they planned to keep the hearing aids after the trial. At the end of the extra 4-week trial with AB service delivery, Session 3B, each participant decided whether to purchase the hearing aids. Figure 16 shows the percentage of those who purchased their hearing aids after Session 3B when the participants in each group are segregated by their original *plans* to keep the hearing aids after the initial trial. One-hundred percent (17 of 17) of group P and 96% (25 of 26) of group CD who *planned* to keep their hearing aids after the initial 6-week trial actually kept them at the conclusion of the study. Of those who were undecided after the initial 6-week trial, 100% (14 of 14) of the P participants and 83% (10 of 12) of the CD participants ultimately kept their hearing aids after the additional 4-week trial. Of course, it is unknown how many in the undecided group would have ultimately kept their hearing aids if the study ended at Session 3 and they were forced to make a final decision at that point in time. For those who indicated in Session 3, after the initial 6-week trial, that they did not intend to keep their hearing aids, 80% (12 of 15) of the P group and 60% (6 of 10) of the CD group ultimately kept their hearing aids after reprogramming and an additional 4-week trial.

At study conclusion, 134 of 154 participants (87%) kept their hearing aids, with small nonsignificant variations in the percentage across initial group assignment (88.7% or AB, 84.3% for CD, and 88% for P), χ^2^ (2) = 0.50, *p* > .05. For the majority of CD and P participants, however, these retention rates are after completion of the additional 4-week AB service-delivery trial. That is, as noted previously, based on their stated *intentions* to keep their hearing aids after the initial 6-week trial, the percentage of “yes” responses were 81% (AB), 55% (CD), and 36% (P). Because “undecided” was a permitted response to this Session 3 query, it is impossible to indicate exactly how many of these respondents would have answered “yes” or “no” if forced to choose.

Although initial assignment to service-delivery group had no impact on the percentage of participants who returned their hearing aids, purchase price did impact returns for credit. Whereas roughly half of those who kept their hearing aids paid the typical purchase price (60 of 134 or 44.8%), 85% (17 of 20) who returned their hearing aids had paid the higher typical purchase price. This effect of purchase-price on the number of returns for credit was significant, χ^2^ (1) = 11.26, *p* < .05.

## Discussion

This first-ever placebo-controlled double-blind randomized clinical trial of hearing aids in older adults with impaired hearing demonstrated the efficacy of the audiology best-practices approach (AB) to hearing aid provision. The outcomes obtained for the participants assigned to the AB treatment were significantly better than those obtained from the participants assigned to the placebo group (P). This was true for the primary outcome measure, PHABglobal; the secondary outcome measure, CST benefit; and other tertiary outcome measures as well (HHIE benefit, HASShaf). Medium or large effect sizes were observed for most of the outcome measures included in this clinical trial. The use of audiology best practices, including the individual evaluation and adjustment of the maximum power output of the hearing aids based on the listener's uncomfortable loudness judgments, did not yield better perception of aversive or distorted sounds (PHABavds) compared to the placebo group. Further, daily usage was not impacted by service-delivery approach.

The outcomes obtained by the participants in the AB group were generally positive, as well as being significantly different from the P group. For example, aided speech-recognition performance for sentences in a noise background (CST) was, on average, 21 RAU better than unaided performance. In addition, perceived benefit improved 17–18 percentage points for the speech-communication scales of the PHAB (PHABglobal) and a measure of perceived hearing handicap (HHIE). Moreover, the average satisfaction ratings for the features and functions of the hearing aids (HASShaf) for the AB group corresponded to a scale value of “satisfied.” Finally, the AB participants wore their hearing aids for 7 hours per day. Again, all of these results are quite positive outcomes and serve to establish that hearing aids do provide benefits to older adults with mild-to-moderate sensorineural hearing loss.

Recall that the CD service-delivery model differed from the AB in two key areas: (a) the AB model made use of real-ear measures to fine-tune the hearing aids to match prescribed NAL-NL2 targets on an individual basis and (b) the AB model included a 45–60 min hearing-aid orientation session. Despite the absence of these two processes from the CD model, this clinical trial also established the efficacy of this simulated OTC service-delivery model. Specifically, the CD group performed significantly better than the P group on the primary outcome measure, PHABglobal; the secondary outcome measure, CST benefit; and on one of the additional outcome measures, HHIE benefit. The CD group, however, demonstrated significantly lower hearing-aid satisfaction scores than the AB group (HASShaf) or both the AB and P groups (HASSdisp). The latter is not surprising, however, given that both the AB and P groups interacted with a professional audiologist during the delivery of the hearing aids, whereas the participants in the CD group had virtually no contact or interaction with a professional during the hearing-aid delivery process. Many of the HASSdisp items pertain to how well the dispenser explained the care and maintenance of the hearing aids, as well as strategies for their use and limitations to their benefits—items covered formally in the hearing-aid orientation by the audiologist for both AB and P participants. As with the AB participants, the CD group did not differ from the other two groups on the measures of perceived aversiveness/distortion of sound (PHABavds) and daily hours of usage.

Aside from the inferior satisfaction ratings from the CD group compared to the AB group noted above, a significantly smaller percentage of CD participants than AB participants indicated that they planned to keep their hearing aids at the conclusion of the trial. Moreover, the outcomes obtained from the CD participants after the extra 4-week AB-based trial were significantly higher for the PHABglobal and HASS outcome measures than the results obtained at the conclusion of the trial. Also, 6 of the 10 CD participants who indicated that they were not planning to keep their hearing aids at the conclusion of the original 6-week trial decided otherwise at the conclusion of the extra 4-week AB-based trial. All told, although both the AB and CD service-delivery approaches were found to be efficacious and to yield generally positive outcomes, these additional metrics may give a slight edge to the AB approach over the CD approach.

The OTC service-delivery approach, one possible version simulated here via the CD model, has been advocated for many years as a way to potentially increase the accessibility and affordability of hearing aids (Donahue et al., 2010; NASEM, 2016; PCAST, 2015; Zimmerman, 2004). It was, in part, for this reason that we also included factorial combinations of purchase price. Because hearing-aid purchase price was not directly manipulated as a factor in a controlled fashion in any prior studies, we opted for its inclusion as a full factorial factor. That is, as conceived, OTC hearing aids are associated with lower purchase prices than conventional hearing aids delivered through AB practices. During study design, we contemplated delivering the hearing aids via the AB service-delivery model with a typical AB purchase price ($3,600) and via the CD service-delivery model with the anticipated OTC/CD purchase price ($600). However, if we had observed differences in outcome at the study's conclusion, we would have been unable to determine whether the differences were due to the service-delivery model, the purchase price, or an interaction between them. As a result, we included purchase price as a part of the factorial design such that both purchase prices were paired with each service-delivery approach. Somewhat surprisingly, except for one significant effect of purchase price considered to be artefactual in nature (CST benefit), there were no significant main effects or interactions of purchase price on the seven outcome measures examined. Perhaps the ratio of purchase prices (6:1) was too small to influence the measured outcomes. Given the annual household income of the majority of trial participants, maybe both dollar amounts used here were too low to influence outcomes. The observation that 85% of those who decided not to keep their hearing aids were in the typical $3,600 purchase-price group, however, suggests that purchase price *did* influence their ultimate decision regarding retention of their hearing aids.

We examined other variables to see if those retaining their hearing aids (*n* = 134) differed significantly from those opting to return them (*n* = 20). Demographic (age, gender, annual income, highest education level, duration of hearing loss), audiological (PTA, HFPTA, LDLs), unaided baselines for outcome measures (PHAPglobal unaided, PHAPavds unaided, HHIE unaided, CST unaided), and MMSE-2 SV cognitive-screen scores were examined. Only hearing loss (both PTA and HFPTA) and age differed significantly between the two groups. Those who returned the hearing aids had less hearing loss and were younger than those who kept them, all *t*(152) values > 2.6, *p* < .05. Specifically, mean PTAs (dB HL), HFPTAs (dB HL), and age (years) were 28.9, 39.6, and 69.6, respectively, for the 134 participants who kept their hearing aids and 21.9, 33.0, and 66.0, respectively, for the 20 participants who returned their hearing aids.

**Figure 17.**
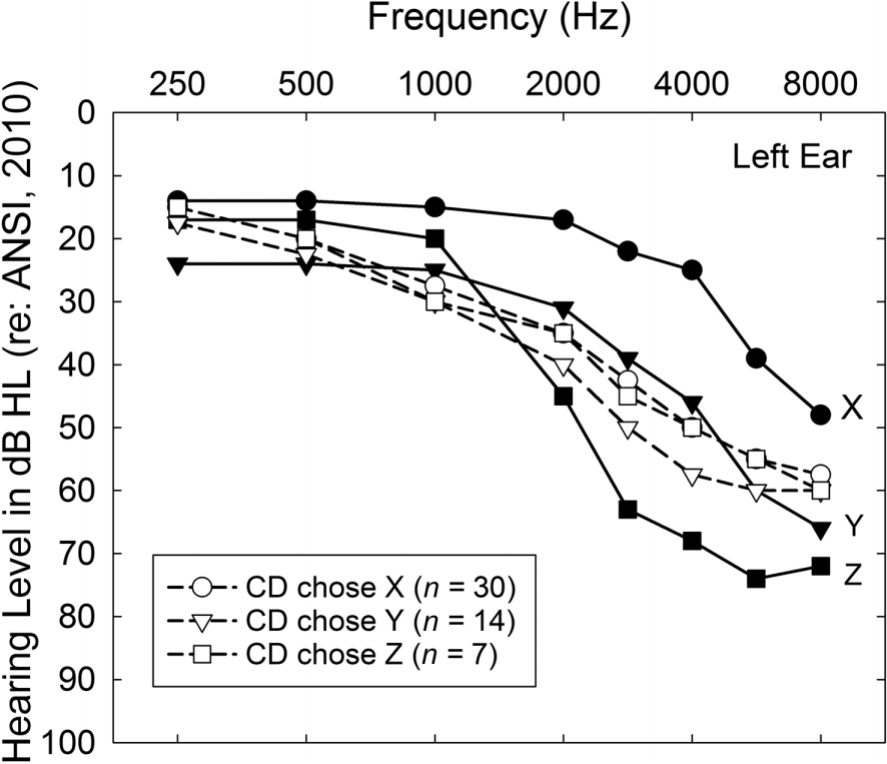
The X, Y, and Z audiograms from Figure 4 (filled symbols) and the median audiograms of the subgroups of consumer decides/over-the-counter (CD) participants (unfilled symbols) who had chosen hearing aids programmed to match NAL-NL2 targets for the X, Y, and Z audiograms. Only data for the left ears of the CD participants are shown here for clarity.

We have also examined in more detail the choices made by the CD participants with regard to the hearing-aid frequency-gain characteristics selected for themselves. Examination of the choice of frequency responses based on the NAL-NL2 targets for audiograms X, Y, or Z (Figure 4) revealed that 65% and 59% of the time hearing aids programmed for audiogram X were selected by CD participants for their right and left ears, respectively. Similarly, hearing aids programmed for the Y audiogram were selected 22% and 28% of the time for the right and left ears, respectively, with the balance (13%) selecting hearing aids that were matched to audiogram Z for both ears. Assuming that the NAL-NL2 targets are valid estimates of desired frequency-gain characteristics, and given that NAL-NL2 only requires hearing thresholds to generate targets, then we would expect “good choices” by the CD participants to be reflected in good matches of their audiograms to the X, Y, or Z audiograms (Figure 4) used to program the hearing aids they selected. Figure 17 shows the median audiograms of the CD participants (unfilled symbols) when they were partitioned into subgroups based on the hearing aid selected (X, Y or Z) during the trial. These data are for the left ear only, but are nearly identical to those for the right ear (not shown). The X, Y and Z audiograms from Figure 4 used to pre-program the hearing aids available to the CD participants are also shown in Figure 17. The median audiogram for the 30 CD participants who selected the X hearing aid (unfilled circles) is much closer to the audiogram used to program the Y hearing aids than to that used to program the X hearing aids. With matches to NAL-NL2 targets as the criterion for a “good match,” these 30 CD participants opted for a hearing aid programmed with less gain in the middle and higher frequencies than prescribed by NAL-NL2. A similar pattern, although not as great a mismatch, is apparent for those CD participants who chose hearing aids programmed on the basis of audiogram Y (*n* = 14; unfilled triangles). In this case, however, the median audiogram for that subgroup falls between the Y and Z audiograms suggesting that either could be a reasonable match. Finally, a small number (*n* = 7; unfilled squares) of CD participants appear to have chosen hearing aids (Z) that likely provided considerably more gain in the middle and higher frequencies than recommended by NAL-NL2.

Rather than rely on the median audiograms for each subgroup of CD participants, we calculated the root-mean-square (rms) error between each participant's audiogram and the X, Y, and Z audiograms used to pre-program the hearing aids. Based on these individual matches, the best-matching audiogram (smallest rms error) among the X, Y, and Z alternatives was selected. Table 9 shows the comparisons between the hearing aid chosen (X, Y, or Z) and the audiogram with the closest match to that of the CD participant. For 24 of the 30 who selected hearing aid X for the left ear and 27 of the 33 who chose X for the right ear, a hearing aid with more gain in the high frequencies, either Y or Z, would have been a more appropriate selection based on threshold-based prescription procedures such as NAL-NL2.

**Table 9.** Comparisons of number of times a particular hearing aid was chosen (X, Y, Z) versus choice based on best match (lowest root-mean-square error) of consumer decides/over-the-counter (CD) participant's audiogram to audiogram X, Y, or Z (see Figure 17) used to program the hearing aids via the NAL-NL2 method.

Hearing aid chosen	Audiogram best match		
	X	Y	Z
Left ear			
X	6	18	6
Y	0	7	7
Z	1	4	2
Right ear			
X	6	23	4
Y	1	6	4
Z	1	4	2

Based on the comparison of median audiograms for CD subgroups who chose hearing aids X, Y, or Z to the X, Y, and Z audiograms used to program those hearing aids (Figure 17) and the examination of best-matching audiograms on an individual basis (Table 9), there is a clear tendency for first-time hearing aid users selecting their own devices to select ones with less high-frequency gain than prescribed by threshold-based procedures such as the NAL-NL2 method used in this trial. Of course, the audiogram mismatch noted here would likely be greater still for threshold-based prescriptive procedures that prescribe more high-frequency gain than NAL-NL2, such as the Desired Sensation Level (DSL) method (Johnson, 2013). The observation that the CD participants self-select hearing aids that are somewhat underpowered may explain some of the inferior outcomes observed in this group compared to the AB participants. On the other hand, the fact that many of the outcomes for the AB and CD participants were similar, including daily usage, suggests that less-expensive OTC options might be a reasonable alternative for older adults to enter the hearing aid market, perhaps seeking assistance from professionals after an initial period of adjustment with their self-selected options. We demonstrated here that following a 6-week CD trial with a subsequent 4-week AB trial significantly improved outcomes and also led to greater retention of the devices. In this case, the additional 4-week AB-based trial immediately followed the initial 6-week trial, but that may not be necessary to obtain the same improvements in outcome.

By study conclusion, there were a total of 147 participants who had completed the AB service-delivery model and provided a full set of outcome measures, 53 original AB participants plus 94 original CD (*n* = 48) and P (*n* = 46) participants who opted for and completed the extra 4-week AB trial. Several 3 × 2 factorial GLM analyses of the effects of *initial* service-delivery group and price group on outcome measures failed to find significant effects of service-delivery, all *F*(2, 140) < 2.23, *p* > .05; price group, all *F*(1, 140) < 2.30, *p* > .05; or their interaction, all *F*(2, 140) < 1.53, *p* > .05, on post-AB outcomes for these 147 participants. For service-delivery group, these results are entirely consistent with the visual impression of the data in Figures 12, 13, 14, and 15, which compared the outcomes for CD and P participants after the 4-week AB treatment (gray bars) to the original AB participants (arrows). As a result, the post-AB data from these 147 participants were pooled. Table 10 shows the pooled means, standard errors, and standard deviations for these 147 post-AB participants. These could be considered to be normative AB-based values for these outcome measures for samples of older adults with mild-to-moderate sensorineural hearing loss and demographics similar to those of the participants in this study (see demographics in Table 3).

**Table 10.** Mean (*M*), standard error of the mean (*SE*), and standard deviation (*SD*) for several outcome measures from this trial all obtained following audiology best practices (AB) service provision (*N* = 147).

Measure	*M*	*SE*	*SD*
Aided PHAPglobal	0.213	0.009	0.112
Aided PHAPavds	0.197	0.012	0.143
Aided HHIE	12.1	1.2	14.6
Aided HASShaf	3.87	0.04	0.52
Aided CST (RAU)	74.1	1.44	17.43
Usage (hours/day)	6.3	0.4	4.3
PHABglobal	0.167	0.010	0.126
PHABavds	0.031	0.012	0.140
HHIE benefit	16.7	1.3	15.4
CST benefit (RAU)	21.9	1.5	18.1

*Note.* PHAPglobal = Profile of Hearing Aid Performance, average of the five communication-related subscales (Familiar Talkers, Ease of Communication, Reverberation, Reduced Cues, and Background Noise); PHAPavds = Profile of Hearing Aid Performance, average of the Aversiveness of Sound and Distorted Sound subscales; PHABglobal = difference between aided and unaided scores of PHAPglobal; PHABavds = difference between aided and unaided scores of PHAPavds; HHIE = Hearing Handicap Inventory for the Elderly; HHIE benefit = difference between aided and unaided HHIE scores; HASShaf = Hearing Aid Satisfaction Survey, items concerning hearing aid features; CST = Connected Speech Test; CST benefit = difference between aided and unaided Connected Speech Test scores; RAU = rationalized arcsine units.

Typically, clinical trials assume that only one or two outcome measures, the primary and secondary outcomes, are to be included. We argued above, based on earlier studies, that this poses challenges for studies of hearing aids as the outcomes appear to be multidimensional, often having three or four primary components (e.g., Humes, 2001, 2003; Humes & Krull, 2012). The data from the 147 post-AB participants for the ten outcome measures appearing in Table 10 were subjected to a principal-components factor analysis (Gorsuch, 1983) to evaluate the redundancy among these measures. As in prior analyses of this type (e.g., Humes, 2003; Humes & Krull, 2012), considerable redundancy was observed among this set of ten outcome measures. In particular, a four-factor solution emerged that accounted for 74.0% of the total variance (Kaiser-Meyer-Olkin [KMO] sampling statistic = .68; all communalities > .60). The loadings of each of the ten variables on the four factors, after orthogonal (varimax) rotation, appear in Table 11. Interpretation of these four factors is fairly straightforward from the pattern of loadings in Table 11. The first outcome factor is aided performance, the second is self-reported benefit, the third is daily usage, and the fourth is aided speech-recognition benefit. Recall that each of these is an orthogonal or independent aspect of hearing aid outcome. Thus, although it is not necessary to include all ten outcome measures noted in this table, it would be equally inappropriate to include only one or two outcome measures to get a comprehensive picture of hearing-aid outcome. Of these four aspects or dimensions of hearing-aid outcome, it could be argued that usage is the least sensitive measure, in that our results demonstrated that AB participants did not differ significantly from the P participants with regard to usage in this trial. On the other hand, most would likely consider usage a fundamental aspect of outcome in that all the other aspects of outcome are trivial if the hearing aids are not worn.

**Table 11.** Rotated (orthogonal) factor loadings of each of the 10 outcome measures on the four outcome factors that emerged from the factor analysis for the 147 post–audiology best practices (AB) participants.

Measure	PC1	PC2	PC3	PC4
Aided PHAPglobal	0.860	—	—	—
Aided PHAPavds	0.598	–0.513	—	—
Aided HHIE	0.827	—	—	—
Aided HASShaf	–0.562	0.406	—	—
Aided CST (RAU)	–0.616	—	—	0.485
Usage (hours/day)	—	—	0.854	—
PHABglobal	—	0.777	—	—
PHABavds	—	0.806	—	—
HHIE benefit	—	0.764	—	—
CST benefit (RAU)	—	—	—	0.852

*Note.* Dashes indicate loadings < 0.40 omitted for clarity. PC1 = first outcome factor, aided performance; PC2 = second outcome factor, self-reported benefit; PC3 = third outcome factor, daily usage; PC4 = fourth outcome factor, aided speech-recognition benefit; HHIE = Hearing Handicap Inventory for the Elderly; HHIE benefit = difference between aided and unaided HHIE scores; HASShaf = Hearing Aid Satisfaction Survey, items concerning hearing aid features; CST = Connected Speech Test; CST benefit = difference between aided and unaided Connected Speech Test scores; RAU = rationalized arcsine units; PHAPglobal = Profile of Hearing Aid Performance, average of the five communication-related subscales (Familiar Talkers, Ease of Communication, Reverberation, Reduced Cues, and Background Noise); PHAPavds = Profile of Hearing Aid Performance, average of the Aversiveness of Sound and Distorted Sound subscales; PHABglobal = difference between aided and unaided scores of PHAPglobal; PHABavds = difference between aided and unaided scores of PHAPavds.

There are several limitations to this study. These include the fairly homogeneous study sample: White, highly educated, with median annual household income greater than $45,000. Generalizing beyond this sample is not appropriate. Further, the CD service-delivery method was implemented as one of many possible OTC approaches to the delivery of hearing aids to older adults. The positive outcomes observed here for the CD approach are specific to that instantiation of an OTC model. Moreover, it is important to note that all participants in this study received a thorough audiological examination in Session 1 to determine candidacy for the trial hearing aids. It is conceivable that some OTC service-delivery models would forego this initial audiological examination. The results from the current trial cannot be used to validate other OTC models or clinical protocols that might exclude any of the protocol procedures completed here. In addition, by design, all participants were fit with the same high-quality devices, programmed with different frequency-gain characteristics based on group assignment. Generalization to other devices is not possible. OTC devices may be considerably simpler and more basic, both electroacoustically (a potential negative) and in terms of coupling (a potential positive), than the conventional mini-BTE slim-tube open-fit hearing aids used in this trial. It should be noted, however, that we have failed previously to observe significant differences in hearing aid outcomes, for outcome measures similar to those in this study, for a wide range of hearing-aid technologies, including some that today would be representative of simpler OTC devices (Humes, Ahlstrom, Bratt, & Peek, 2009). More recently, Cox and colleagues have demonstrated equivalent outcomes between basic and advanced contemporary hearing aids (Cox, Johnson, & Xu, 2014, 2016; Johnson, Xu, & Cox, 2016). Nonetheless, the results from this study using high-quality hearing aids cannot be generalized to other devices, including those more likely to be used in OTC approaches to hearing aid delivery.

One could argue that the AB model evaluated here, including real-ear verification of targets, individual adjustment of maximum power output, and a hearing-aid orientation and counseling session, may still not be representative of “audiology best practices.” True best practices would also include follow-up appointments during the 6-week trial to adjust the devices in response to patient complaints. Guidelines for best practices in delivering hearing aids to adults from the American Academy of Audiology (Valente et al., 2006) and the American Speech-Language-Hearing Association (ASHA, 2015), however, do not include such fine-tuning sessions as a part of best practices. Further, the only available peer-reviewed research on this practice (Cunningham, Williams, & Goldsmith, 2001) indicates that such fine-tuning during follow-up sessions has no significant effect on measured outcomes. Finally, assuming that real-ear measurements would guide such adjustments of responses away from targets, it is unlikely that this is a widespread practice given the continued low use of real-ear verification in general by audiologists (e.g., Mueller & Picou, 2010). Thus, we do not believe that the AB model evaluated here was an inappropriately stripped-down version of true best practices that may have minimized differences in outcome between it and the CD model.

On the other hand, one could argue that the CD participants experienced some aspects of audiology best practices, including a full audiological evaluation and medical clearance or waiver of such, and that this may have optimized the outcomes for the CD group. At the time this trial was initiated, the FDA required medical clearance (or waiver of such) for all hearing aids provided to consumers and, given what we knew at study inception, we considered it unethical to provide hearing aids to participants without a full audiological evaluation. It also could be argued that the restriction of the choice of devices to only three responses already pre-programmed for commonly encountered hearing losses to match NAL-NL2 targets, on average, and using the same hearing aids as those in the other two branches, unnecessarily constrained the range to be encountered in true OTC approaches. Again, it is acknowledged that the CD instantiation of the OTC approach here is just one of many possibilities, and it is up to others to validate alternative OTC approaches. However, the CD model does not seem to be too farfetched as an early approach to the delivery of OTC devices. Given the positive outcomes observed for the CD approach in this study, as well as the FDA's recent elimination of the medical-clearance requirement for hearing aids and its exploration of OTC devices as an alternative to hearing aids, the CD approach could certainly serve as a starting point against which other OTC service-delivery options could be compared.

Finally, with regard to improvements in outcomes for the CD and P participants re-examined after an additional 4-week AB-based trial, it should be kept in mind that there could very well be an order bias such that scores the second time around may be higher due to additional experience with the hearing aids. We did not have participants assigned initially to the AB group complete a 4-week CD or P trial to counterbalance the order of service-delivery approaches. Our purpose for including the additional 4-week trial was based on the ethical treatment of participants and our state of the knowledge base at the inception of the clinical trial. Based on the knowledge available at the start of this clinical trial, the AB approach was considered superior and, due to random assignment to CD or P groups, those participants should not be deprived of a presumably superior service-delivery approach. AB participants could opt for an additional 4 weeks to evaluate adjustments to their devices, but they did not receive an alternative service-delivery model. On the other hand, arguing against a significant order effect or experience-based explanation of outcome improvements from Session 3 to Session 3B is the systematic nature of the improvements across outcome measures. For example, neither group showed improvement in PHABavds or usage. The no-gain P group showed significant improvements in PHABglobal, CST benefit, and HHIE benefit once adequate gain was provided. The CD group also showed significant improvement in PHABglobal, despite having a reasonable amount of gain in both Session 3 and Session 3B, but this could be due to better use of communication strategies discussed in the hearing-aid orientation. Finally, as indicated by the arrows showing the means from the original AB participants in Figures 12–15 and the subsequent analyses of the 147 post-AB participants, the average performance of the original CD and P participants following a 4-week AB-based extended trial did not differ significantly from that of the original AB participants who had only a single 6-week trial.

## Conclusions

This single-site double-blind placebo-controlled randomized clinical trial is the first to demonstrate that hearing aids are efficacious in older adults for an audiology-based best-practices (AB) service-delivery model. The efficacy of an alternative OTC approach (CD) in which the consumer selected pre-programmed devices was also established. Overall, the CD model of OTC service delivery yielded only slightly poorer outcomes than the AB model. Nonetheless, outcomes for CD participants (as well as P participants) improved significantly following a 4-week AB-based follow-up trial. Purchase price, $600 versus $3,600 per pair of hearing aids, had no effect on outcomes, but a high percentage (85%) of those who rejected hearing aids paid the higher of the two prices for their devices. Efficacious OTC service-delivery models (and devices) may increase accessibility and affordability of hearing aids for millions of older adults, but further research is required to evaluate various devices and approaches, as well as to examine the generalization of the findings from this clinical trial.

## Supplementary Material

10.1044/2017_AJA-16-0111SMS1 Supplemental Material S1. ABCD Protocol Manual.

10.1044/2017_AJA-16-0111SMS2 Supplemental Material S2. Data-collection forms.

10.1044/2017_AJA-16-0111SMS3 Supplemental Material S3. Instructional video for participants.
