# Supplementary material for: The Effects of Service-Delivery Model and Purchase Price on Hearing-Aid Outcomes in Older Adults: A Randomized Double-Blind Placebo-Controlled Clinical Trial
Source: Am J Audiol. 2017 Mar;26(1):53–79. doi: 10.1044/2017_AJA-16-0111 (PMC5597084; doi:10.1044/2017_AJA-16-0111)
Supplement: Supplemental Material S1. [file AJA-26-53-s001.pdf]

Supplemental Material S1. ABCD Protocol Manual.

Evaluating Hearing Aid Service-Delivery Models (ABCD) Protocol

Audiology Research Laboratory (ARL)

Department of Speech and Hearing Sciences

Indiana University

Bloomington, IN 47405 USA

November 2, 2012

Supported, in part, by NIDCD research grant R01 DC011771

Larry E. Humes (PI), Christine Herring, Dana Kinney, Anna Main, Tera Quigley and Sara Rogers

Preliminary Notes:

- When completing various forms for this clinical trial, use black ink only. If a mistake is made, cross it out and initial.
- Review every form for completion. Identify missing items with participant and if the participant does not wish to complete for any reason note “did not complete” in the space provided. Do not leave any section blank.
- In the event of an adverse event, the Medical Officer is to be contacted and a current Prompt Reporting Form will need to be downloaded from the Office of Research Administration website and submitted within 24 hours of the event.
- Forms used can be found in the appendix. Forms are cited as “(x-y)” in this manual where “x” is the session number and “y” is the sequential form number in that session.

TABLE OF CONTENTS

I. SESSION 1 – AUDIOLOGY RESEARCH LAB (ARL) 5

A. PHONE SCRIPT (1-1) 6

B. SCREENING CONSENT FORM #1 (1-2) 6

C. HIPAA FORM (1-3) 6

D. PERMISSION TO CONTACT FORM (1-4) 7

E. DEMOGRAPHIC FORM (1-5) 7

F. AUDIOLOGIC EVALUATION [AUDIOGRAM (1-6), INSTRUCTIONS FOR LOUDNESS TEST (1-7), CATEGORIES OF LOUDNESS (1-8) 7

G. UNAIDED CST (CONNECTED SPEECH TEST) FORM (1-9) 10

H. COUNSELING 11

I. MMSE-2 SV (MINI MENTAL STATE EXAMINATION, SECOND EDITION, STANDARD VERSION) (1-11) 12

J. CASE HISTORY (1-12) 12

K. ELIGIBILITY PART 1 (1-13) 12

L. MEASURE RECD 13

M. SESSION 1 CHECKLIST (1-14) 15

N. STUDY CONSENT FORM #2 (1-15A FOR TYPICAL PURCHASE PRICE GROUP OR 1-15B FOR REDUCED PURCHASE PRICE GROUP), MEDICAL WAIVER (1-16) AND ELIGIBILITY PART 2 (1-17) 15

II. SESSION 2 –ARL 18

A. UNAIDED PHAP (PROFILE OF HEARING AID PERFORMANCE) (T-1) 19

B. UNAIDED HHIE (HEARING HANDICAP INVENTORY FOR THE ELDERLY) (T-2) 19

C. HEARING AID SELECTION AND FITTING 20

D. UNSCHEDULED VISITS (ALL GROUPS) (M-1) 37

III. SESSION 3 – ARL 39
A. PARTICIPANT ARRIVAL 40
B. AS-WORN AIDED CST FORM (3-1) 40
C. PHYSICAL INSPECTION OF HEARING AIDS (3-2) 42
D. AS-WORN SIMULATED SPEECHMAP (RIGHT, THEN LEFT) (3-2) 42
E. HEARING AID MAINTENANCE (3-2) 43
F. AIDED PHAP (T-3), AIDED HHIE (T-4) AND HASS (HEARING AID SATISFACTION SURVEY) (T-5) 44
G. POST-MAINTENANCE AIDED CST FORM (3-3) 44
H. PHAST-R (PRACTICAL HEARING AID SKILLS TEST-REVISED) (T-6) 45
I. MUSIC/TRAINING QUESTIONNAIRE (3-4) 45
J. FINAL HEARING AID DECISION FORM #1 (3-5) 46
K. REVEAL GROUP DESIGNATIONS (3-5) 46
L. DATA LOGGING FORM (3-6) 46
M. PROGRAMMING AND/OR ADJUSTMENT OF HEARING AIDS (3-7) 48
N. HEARING AID ADJUSTMENT LOG (3-8) 52
O. HEARING AID QUESTIONS FORM (3-9) 52
P. HEARING AID STATUS FORM #1 (3-10) 52
Q. SCHEDULE FOLLOW-UP 52
IV. SESSION 3B – ARL 53
A. PARTICIPANT ARRIVAL 54
B. AS-WORN AIDED CST FORM (3B-1) 54
C. PHYSICAL INSPECTION OF HEARING AIDS (3B-2) 56
D. AS-WORN SIMULATED SPEECHMAP (RIGHT, THEN LEFT) (3B-2) 56
E. HEARING AID MAINTENANCE (3B-2) 57

F. AIDED PHAP (T-3), AIDED HHIE (T-4) AND HASS (T-5) 58

G. POST-MAINTENANCE AIDED CST FORM (3B-3)... 58

H. PHAST-R (T-6)... 59

I. MUSIC/TRAINING QUESTIONNAIRE (3B-4) 59

J. HEARING AID DECISION FORM #2 (3B-5) 60

K. DATA LOGGING FORM (3B-6)... 60

L. ADJUSTMENT OF HEARING AIDS (3B-7).. 61

M. HEARING AID ADJUSTMENT LOG (3B-8)... 62

N. HEARING AID QUESTIONS FORM (3B-9).. 62

O. HEARING AID STATUS FORM #2 (3B-10)... 62

P. SCHEDULE FOLLOW-UP 62

I. SESSION 1 – AUDIOLOGY RESEARCH LAB (ARL): 60-90 MINUTES REQUIRED

<u>Study personnel</u>

CTC: Clinical Trials Coordinator

A1: Audiologist 1

A2: Audiologist 2

<u>Components</u>

A. CTC-PHONE SCRIPT (1-1)
B. CTC-SCREENING CONSENT FORM #1 (1-2)
C. CTC-HIPAA FORM (1-3)
D. CTC-PERMISSION TO CONTACT FORM (1-4)
E. CTC-DEMOGRAPHIC FORM (1-5)
F. A1/A2-AUDIOGRAM (1-6), INSTRUCTIONS FOR LOUDNESS TEST (1-7), CATEGORIES OF LOUDNESS (1-8) AND TYMPANOMETRY
G. A1/A2-UNAIDED CST FORM (1-9)
H. COUNSELING [EXIT FORM #1 (1-10) IF INELIGIBLE]
I. A1/A2-MMSE-2 SV (1-11)
J. A1/A2-CASE HISTORY (1-12)
K. A1/A2-ELIGIBILITY PART 1 (1-13)
L. A1/A2-MEASURE REAL-EAR-TO-COUPLER-DIFFERENCE (RECD)
M. SESSION 1 CHECKLIST (1-14)
N. CTC- STUDY CONSENT FORM #2 (1-15A OR 1-15B), MEDICAL WAIVER (1-16) AND ELIGIBILITY PART 2 (1-17)

Forms Needed:

Phone Script (1-1), Screening Consent Form #1 (1-2), HIPAA Form (1-3), Permission to Contact Form (1-4), Demographic Form (1-5), Audiogram (1-6), Instructions for Loudness Test (1-7), Categories of Loudness (1-8), Unaided CST Form (1-9), Exit Form #1 (1-10, needed only if not eligible for study), Mini Mental State Exam (MMSE-2, SV) (1-11), Case History (1-12), Eligibility Part 1 (1-13), Session 1 Checklist (1-14), Study Consent Form #2 (1-15a Or 1-15b), Medical Waiver (1-16), Eligibility Part 2 (1-17),

A. PHONE SCRIPT (1-1)

1. Participant calls CTC in response to advertisement.
2. Phone Script (1-1): CTC explains nature of study, verifies eligibility, and schedules 2 appointments [Session 1 and Session 2 (the day after Session 1)].

B. SCREENING CONSENT FORM #1 (1-2)

1. Participant arrives at CTC's office.
2. CTC instructs participant to read the consent form, initial at bottom of pages where indicated, and sign form. CTC provides the participant with a copy of the consent form. CTC reviews the general nature of the study including eligibility criteria, approximate number of sessions and compensation (free audiologic evaluation).
3. CTC assigns participant number at this time and labels Session 1 folder with participant number.

C. HIPAA FORM (1-3)

1. CTC instructs participant to read the HIPAA Form (1-3), fill in their personal information, initial, and sign where indicated.

D. PERMISSION TO CONTACT FORM (1-4)

1. This form requests permission from the participant to contact him or her about future studies. CTC explains that signing it only allows us to contact him or her about possible participation; it does not mean that he or she is required to participate in future studies.

E. DEMOGRAPHIC FORM (1-5)

1. Participant fills out numbers 1, 2, and 3 of this form pertaining to the participant's gender, race and ethnicity, respectively. CTC completes remainder of form. This is an optional form for the participant. If the participant declines to fill out the form, CTC should complete form to the best of their ability.
2. CTC stores Demographic Form (1-5) in separate location from other participant information, and pages A1 or A2 to meet new participant and begin audiologic evaluation.
3. CTC gives A1 or A2 folder with the participant number on it that contains Unaided CST Form (1-9), MMSE (1-11), Case History (1-12) and Eligibility Part 1 (1-13).
4. The participant's spouse or guest must remain in the waiting room during audiologic evaluation.

F. AUDIOLOGIC EVALUATION [AUDIOGRAM (1-6), INSTRUCTIONS FOR LOUDNESS TEST (1-7), CATEGORIES OF LOUDNESS (1-8)]

1. Complete otoscopic examination prior to audiometric or tympanometric testing to check for ear canal obstruction and/or eardrum abnormalities.
2. Complete tympanometry and acoustic reflex testing using the GSI 39 Auto Tymp.
   a. If not already set up for required reflex measurements after tympanogram, press "Tymp/Reflex" button, then press "Ipsi" and finally press the frequencies "500 Hz,

1000 Hz and 2000 Hz".

b. Tympanometry: 226-Hz probe frequency. Follow instructions on the machine.

c. Ipsilateral acoustic reflex testing at frequencies: 500, 1000 and 2000 Hz will happen automatically following tympanometry.

d. Record ipsilateral acoustic reflex thresholds on audiogram.

3. Seat participant in designated chair in the booth (make sure chair is located in correct position on the floor).

4. Obtain pure-tone air conduction thresholds using ER-3A insert earphones and ASHA standard procedures using the GSI 61 audiometer in the ARL. Set "transducer" to "insert".

   a. AC Frequencies: 250, 500, 1000, 1500, 2000, 3000, 4000, 6000, and 8000 Hz. Apply masking as needed.

   b. If it is now known that participant is ineligible based on their hearing loss: complete speech testing (SRT and WRS) and bone-conduction testing, counsel participant (see H. below).

      (1) Note on audiogram "Comments" section that participant is ineligible based on hearing loss, whether her or she is a viable hearing aid candidate, and if we will follow up with participant in one year.

      (2) Note if a referral to ENT was made or other advice was given.

5. Speech testing (keep ER-3A insert earphones in)

   a. Administer Speech Reception Threshold (SRT) testing with monitored live voice (MLV).

   b. Administer CID W-22 word-recognition test at 40 dB SL re: the SRT level using

stimuli on CD ("VA Speech Recognition and Identification Materials").

(1) Audiometer settings: Channel 2, External B

(2) Track 1: calibrate

(3) Track 3: list 1A

6. Measure Loudness Discomfort Levels (LDL) at 500 and 3000 Hz

a. Give participant laminated Instructions for Loudness Test (1-7) and Categories of Loudness (1-8). Let the participant read over and verify understanding.

b. Reinstruct participant prior to testing:

(1) "The purpose of this test is to find your judgments of the loudness of different sounds. You will hear sounds that increase and decrease in volume. You must make a judgment about how loud the sounds are. Pretend you are listening to the radio at that volume. How loud would it be? After each sound, tell me which of these categories best describes the loudness. Keep in mind that an uncomfortably loud sound is louder than you would ever choose on your radio no matter what mood you are in."

c. Stimuli: warble tone at 500 Hz followed by 3000 Hz

d. Participant will respond to varied presentation levels on seven-point scale of loudness using the Categories of Loudness (1-8) as a reference.

e. Procedure: start at threshold and use an ascending approach in 5 dB steps until an "uncomfortably loud" response is obtained. Return to threshold and repeat for a total of 4 times.

f. The median of the values obtained on four runs may be assumed to represent the loudness percept in each category.

g. Record on audiogram.

7. Put bone vibrator on participant's mastoid and administer pure-tone bone-conduction (BC) testing using ASHA standard procedures using the GSI 61 audiometer. Set "transducer" to "bone".

    a. BC test frequencies: 250, 500, 1000, 1500, 2000, 3000, and 4000 Hz. Apply masking as needed.

8. To be eligible for ABCD, participant's hearing thresholds need to be bilaterally symmetrical and within a mild to moderate sensorineural hearing loss range, and within fitting range of hearing aid. *See shaded region on Audiogram (1-6).

    a. Asymmetry: more than 20 dB difference at any one frequency 250-2000 Hz makes participant ineligible.

    b. Participant may have one threshold at a single frequency in each ear that is outside the shaded area by ≤ 10 dB and still be eligible for study.

    c. If participant's thresholds are close to, but outside our accepted range, "flag" the file and notify PI and CTC.

    d. If participant is eligible, continue with unaided CST.

    e. If ineligible, counsel (see H below).

        (1) Note on audiogram if a referral to ENT was made or other advice was given.

G. UNAIDED CST (CONNECTED SPEECH TEST) FORM (1-9)

1. Audiometer settings:

    a. Track 1: Calibrate—complete prior to every test

    b. Channel 1: 50 dB HL, External A, Left (front speaker), Speaker

    c. Channel 2: 46 dB HL, External B, Right (back speaker), Speaker

2. Instructions: “You will hear a woman talking from in front of you. There will be noise behind you. Ignore the background noise, and try to repeat back what the woman says. She will say one sentence at a time. Each group of sentences has a topic. The first topic is “window.” Do you have any questions?”
3. Present CST (track 32) at 50 dB HL from loudspeaker located at 0˚ azimuth and elevation with the multi-talker babble from the CST presented at 46 dB HL (+3 dB signal-to-babble ratio) from an identical loudspeaker located at 180˚ azimuth and 0˚ elevation.
4. Have participant complete two passage pairs (“window” and “glove”; 50 total key words). Give participant second passage topic prior to starting second passage.
5. Present sentences one at a time, pausing the CD after each sentence to score.
6. Key words are capitalized and bolded on Unaided CST Form (1-9). To score, cross out key words that are repeated incorrectly or are not repeated at all.
7. Participant must repeat the word exactly as written for correct response. Any other variation of the word, incorrect word, or absence of the word should be marked incorrect.
8. Score = total number of correct words. Record as percentage score next to total correct score.

H. COUNSELING

1. Ask participant if anyone is waiting in the waiting room that he or she would like to have present during counseling re: hearing test. If yes, go get person in waiting room.
2. Counsel participant regarding audiologic evaluation results.
3. If participant is ineligible, escort out of building and inform CTC that participant’s

tentative appointment needs to be cancelled. Exit Form # 1 (1-10) is completed and signed at this time. If participant's thresholds were close to our acceptable range, notify participant that we will contact him or her if requirements change.

I. MMSE-2 SV (MINI MENTAL STATE EXAMINATION, SECOND EDITION, STANDARD VERSION) (1-11)

1. Follow the instructions on the MMSE-2 SV (1-11). Participant needs to score at least 26/30 to pass. Record the score on the MMSE-2 SV (1-11).
2. If participant is still eligible, continue to Case History.

J. CASE HISTORY (1-12)

1. Ask the participant to fill out this form with a black pen. Be available for any questions that may arise while filling it out. If the participant has a lengthy list of medications, make a copy of this and staple it to the case history form.
2. A1/A2 will review Case History (1-12) to make sure no section is left blank. If there is a blank section, address this with the participant and either have him or her fill in the blank or make a note as to why this section was left blank.

K. ELIGIBILITY PART 1 (1-13)

1. The top portion of the Eligibility Part 1 (1-13) will be checked off after reviewing the Case History (1-12) for completeness.
2. Ask the participant the remaining questions (# 2, pertaining to bilateral flat, type B, tympanograms will be evident during the hearing test). If the answer to any of the exclusion criteria is "yes", the participant is not eligible.
3. If participant is still eligible, continue to Real-Ear-to-Coupler-Difference (RECD).

L. MEASURE RECD

1. On the Audioscan Verifit, connect RECD transducer to the RECD jack on the front of the test chamber.
2. Click: “Tests”→”On-ear measures”→”RECD”
3. Coupler measurement needs to be completed daily. Connect RECD transducer to the BTE coupler (see below).

Image not shown here due to potential copyright issues. Please see page 77 in the Audioscan Verifit User's Guide Version 3.4.

4. Click “Measure coupler” and follow setup instructions.
   a. Click “Continue” to generate the coupler response curve (green). When the curve is stable, click “Continue” to save it.
   b. Insert probe tube into ear canal to within 2-5 mm of the eardrum. Insert foam tip into ear, flush with entrance to ear canal, and allow to fully expand.
   c. Click “Continue” to generate the real-ear response curve (pink) and RECD curve (cyan).
      (1) If RECD curve is negative and unstable in low frequencies, check seal.
      (2) If RECD curve deviates more than 10 dB from the age-related average in 4-6 kHz region check for proper probe placement.

Image not shown here due to potential copyright issues. Please see page 79 in the Audioscan Verifit User's Guide Version 3.4.

d. When curves are stable press “Continue” to save.

e. Complete RECD measurements on both ears using “Dual View” mode to display both figures on screen at once.

f. Change format setting to “Table”.

g. Insert USB flash drive into Verifit USB port.

   (1) Select “Print”, enter file name, and press “Continue” to save to USB.

   (2) Save as an XML file:

      (a) Press “Session” on Verifit, then select “Store session file”.

      (b) Select the “Client ID” box and enter file name and press “Continue”. This will save file in “Sessions” folder on USB flash drive along with a style sheet (audioscan.xsl).

h. When RECD measurements are complete for both ears, disinfect probe tubes with two disinfectant wipes, dry with paper towel, and place in envelope with participant number on it which will go in folder.

i. Print RECD measurements from USB flash drive and place in Session 1 folder.

j. Take participant and participant's folder to CTC.

M. SESSION 1 CHECKLIST (1-14)

1. Ensure all portions of the checklist are completed.

N. STUDY CONSENT FORM #2 (1-15A FOR TYPICAL PURCHASE PRICE GROUP OR 1-15B FOR REDUCED PURCHASE PRICE GROUP), MEDICAL WAIVER (1-16) AND ELIGIBILITY PART 2 (1-17)

1. CTC reviews the general nature of the study including approximate number of sessions and the requirement to purchase hearing aids.
2. CTC informs participant of the FDA requirement for medical clearance. If participant elects to waive the right to medical evaluation/clearance and is interested in participating in the clinical trial, he or she will sign the Study Consent Form #2 (1-15a or 1-15b) and the Medical Waiver (1-16).
    a. If participant does not waive the medical evaluation and clearance, the participant is not eligible for the study. Only participants who choose to waive medical clearance prior to hearing aid purchase are accepted to the study. If participant does not wish to waive medical evaluation and clearance, participant should be referred to the IU Hearing Clinic.
3. CTC will complete Eligibility Part 2 (1-17). If the answer to any of the exclusion criteria questions is "yes", the participant is not eligible.
4. If participant is not eligible, or is eligible but does not want to participate in study, CTC will cancel the Session 2 appointment and escort participant out of building.
5. If participant agrees to participate, signs Study Consent Form #2 (1-15a or 1-15b), completes the Medical Waiver (1-16), and does not answer "yes" to any of the exclusion criteria, CTC will assign participant to one of 6 possible treatment groups.

a. Using the unaided CST score, CTC will place participant in a low (≤ 40% correct), medium (40% < score < 68%), or high (≥ 68% correct) performance group, and use this information for randomizing the participant into a treatment group.

b. Participant groups are stratified by purchase price. For the initial half of the study, participants are randomized into treatment groups (AB, CD, P) using the typical purchase price (TPP). Then, for roughly the second half of the study, participants are randomized into treatment groups using the reduced purchase price (RPP). Within each purchase price group, subjects are randomized to one of three treatment groups: audiology-based (AB), placebo (P-AB), or consumer-decides (CD) using pre-generated random orders yielding roughly equal proportions of AB, CD and P group assignments. The possible treatment groups include: P-AB/TPP, P-AB/RPP, AB/TPP, AB/RPP, CD/RPP, and CD/TPP. (NOTE: Sect 5.b, was edited AFTER STUDY COMPLETION to reflect what actually took place. The shared protocol manual used by the Clinical Trials Group indicated that the purchase price was also RANDOMLY DETERMINED for each participant enrolled. Audiologists A1, A2, A3 and A4 were unaware of this deception re: the ordering of purchase price assignment as was confirmed in individual interviews with the audiologists by the PI at study completion.)

c. Depending on the purchase price being used, CTC discusses the purchase price with the participant, which is due, in full, at the time of Session 2.

d. CTC informs the participant that a 16.7% discount on purchase price will be provided by the completion of 3 sessions.

6. CTC tells participant: “The audiologists are involved in only the research portion of this

project; therefore, they will not know information about purchase price. I am the business contact, so if you have questions please contact me.” CTC gives participant a business card.

7. At this time, CTC verifies the appointment to come in the next day for Session 2.
8. A copy of the participant’s completed Audiogram (1-6), RECD measurements, and an envelope containing the probe tubes used during RECD should be placed in Session 2 folder for hearing aid programming.

II. SESSION 2 –ARL: 120-150 MINUTES REQUIRED

Study personnel

CTC: Clinical Trials Coordinator

A1: Audiologist 1

A2: Audiologist 2

A3: Audiologist 3

Components

A. CTC- UNAIDED PHAP (T-1)
B. CTC- UNAIDED HHIE (T-2)
C. A1/A2/CTC- HEARING AID SELECTION AND/OR FITTING (BASED ON GROUP ASSIGNMENT)
    4. AUDIOLOGY-BASED (AB) GROUP: PAGES 20-28
    5. PLACEBO (P-AB) GROUP: PAGES 28-34
    6. CONSUMER-DECIDES (CD) GROUP: PAGES 34-36
D. UNSCHEDULED VISITS (M-1)

Forms/Materials Needed: Tablet for Unaided PHAP (T-1) and Unaided HHIE (T-2), Session 2 Audiologist Checklist (2-1), Hearing Aid Decision Form for Audiologist (2-2), Orientation Checklist (2-3), User Guide (2-4, inside hearing aid case), Hearing Aid Case Checklist for CTC (2-5), CD Rx Form (2-6), New Hearing Aid Check-in Protocol (M-2), Black binder (2-7), Red binder (2-8), Blue binder (2-9), Hearing Aid Decision Form for CTC (2-10), Unscheduled Visit Checklist (M-1), Audiogram from Session 1 (1-6), RECD measurement from Session 1, envelope with probe tubes from Session 1.

A. UNAIDED PHAP (PROFILE OF HEARING AID PERFORMANCE) (T-1)

1. Participant arrives at CTC's office and CTC provides participant with the Samsung Series 7 Slate tablet to complete the Unaided PHAP in the waiting room.
   a. If the participant has a spouse or guest along, the guest must remain in a different room until self-report measures are completed. The guest may join the participant when the hearing aid orientation begins (ABgroups), or when the CTC escorts the participant to the Clinical Trial Hearing Aid Room (CD group).
2. Enter participant number.
3. Instruct participant to follow the instructions and complete the Unaided PHAP (T-1) using the tablet. Instruct participant to press the button on pager device when finished.
4. CTC will transfer results to the server. CTC will also print the output file from the tablet and place into participant's Session 2 folder.
   a. Once results are printed and files have been copied to the server, CTC will delete the files from the USB and the tablet.

B. UNAIDED HHIE (HEARING HANDICAP INVENTORY FOR THE ELDERLY) (T-2)

1. Enter participant number.
2. Instruct participant to follow the instructions and complete the Unaided HHIE (T-2) using the tablet. Instruct participant to press the button on pager device when finished.
3. CTC will transfer results to the server. CTC will also print the output file from the tablet and place into participant's Session 2 folder.
   a. Once results are printed and files have been copied to the server, CTC will delete the files from the USB and the tablet.

C. HEARING AID SELECTION AND FITTING

1. If the participant is in the CD group, CTC escorts the participant and his or her guest (if applicable) to the Clinical Trial Hearing Aid Room.
2. If the participant is in one of the AB groups (AB or Placebo), CTC pages A1 or A2 (preferably the same audiologist who saw the participant for Session 1).
3. Find the corresponding group below and follow the remainder of the instructions to complete Session 2.
   a. AB- see #4 below
   b. P- see #5 below
   c. CD- see #6 below
   d. Group designation is indicated by a post-it note the CTC placed inside the participant's Session 2 folder.
4. **AB group hearing aid selection and fitting**
   a. A1/A2 escorts participant to participant side of audio booth.
   b. Turn chair and place within marks on the floor and position external speaker within marks on the floor for real-ear testing. Participant is seated in this chair for hearing aid programming.
   c. Hearing aid color and dome choice
      (1) Participant chooses hearing aid color. Audiologist chooses appropriate dome size and tubing length using hearing aid manufacturer's tubing measurement tool.
   d. Program hearing aids
      (1) Audiologist places Audiogram (1-6) on lap to be in view of the participant to

mimic what occurs in typical audiology clinic

(2) Enter participant number and audiogram into Noah, then click Aventa in upper right corner.

(3) Connect hearing aid and generate first fit in the manufacturer's fitting software using NAL-NL2 fitting algorithm.

(4) Fitting should default to "Experienced Non-Linear".

(5) Select "Fitting" tab (top toolbar) and turn "Binaural Correction" off.

(6) Change "Physical Properties" to the size of dome and tube chosen.

(7) Change P1 to "Basic+Softswitching" if it is not already on that setting.

(a) Directionality: Fixed

(b) Directional Mix: Very Low

(c) DFS Ultra: Moderate

(d) Expansion: Off

(e) Noise Tracker II: Per Environment

(f) Wind Guard: Off

(8) Click on "Environmental Optimizer" and click "Reset to…" then select "0 dB" so that all levels are at zero.

(9) If applicable, click on "Tinnitus Sound Generator" and ensure it is off.

e. Real-ear measurement on Verifit

(1) Use Verifit to obtain real-ear measurements to verify hearing aids meet NAL-NL2 target output for speechmap.

(2) "Tests"→ "On-ear measures"→ "Speechmap"

(3) Enter participant's RECD measurements along with pure-tone air-conduction

thresholds.

(4) Insert one probe tube in each ear.

 (a) Check that the probe tube is connected to the appropriate R/L cord.

 (b) Check that each reference microphone is facing outwards.

 (c) Mute hearing aids prior to insertion.

 (d) Choose "Open" as the hearing aid type on the Verifit.

 (e) Click "Equalize" while hearing aids are still muted. After noise has stopped, un-mute hearing aids.

(5) Run speechmap with "carrot passage" speech stimulus at 65 dB SPL and then fine-tune gain until it matches targets appropriately.

 (a) Target-matching rule: for 250 through 4000 Hz, all measured values within 4 dB of target at all frequencies in this range is the goal, but will accept fit after initial fine-tuning attempts if within 7 dB of target at all frequencies in this range.

 (b) If unable to meet + or – 7 dB criterion, participant will not be exited from the study, but we will likely need to replace that participant in the study. If this happens, "flag" the file and let PI and CTC know.

(6) Run speechmap at 55 dB SPL and 75 dB SPL. Do not fine tune hearing aids following these measurements.

(7) Record SII measurements for 65, 55, and 75 dB SPL inputs for both ears on Session 2 Audiologist Checklist (2-1).

 (a) Do not make hearing aid adjustments based on participant feedback until outcome measures are completed in Session 3.

f. Loudness discomfort measurement

(1) Categories of Loudness (1-8) should be in front of participant (participant was familiarized with this during audiologic evaluation).

(2) Re-familiarize participant with loudness categories and instruct:

(a) "You will hear several loud beeps that get higher in pitch. The beeps will be loud, but if they get "uncomfortably loud" (refer to Categories of Loudness), raise your hand. The beeps are very quick, so try to raise your hand as quickly as possible. We can repeat if necessary."

(3) Run the 85 dB SPL MPO stimulus with the hearing aid on in right ear only. Left hearing aid should be muted.

(4) If participant indicates a tone is "uncomfortably loud", decrease maximum output at corresponding frequency respectively by 4 dB, record on Session 2 Audiologist Checklist (2-1), and re-initiate 85 dB SPL stimuli.

(5) Continue until "uncomfortably loud" responses are no longer obtained.

(6) Repeat with left hearing aid on and right hearing aid muted.

(7) Finally, turn both hearing aids on and run the MPO stimulus. If participant reports none of the sounds are "uncomfortably loud" move on to programming VC (g). If participant reports one of the sounds uncomfortably loud, determine which frequency to lower by 2 dB in both hearing aids until "uncomfortably loud" response is no longer obtained. Record any adjustments on Session 2 Audiologist Checklist (2-1).

(8) Before removing hearing aids, review "beeps" with participant by clicking on "Beeps and Volume Control" in Aventa software and presenting each beep

sample (delayed start, low battery, and program change), verifying that beeps are loud enough.

(9) Change format settings to “Table” on Verifit speechmap screen.

(10) Insert USB flash drive into Verifit USB port.

(a) Select “Print” and enter file name and press “Continue” to save to USB flash drive.

g. Program VC based on HFPTA

HFPTA (1,2, and 4kHz)	VC P1	VC P2	VC P3	VC P4
≤21	0	-2	-4	2
22-29	0	-3	-6	3
30-38	0	-4	-8	4
39-47	0	-5	-10	5
48-55	0	-6	-12	6
56-64	0	-7	-14	7
≥ 65	0	-8	-16	8

(1) Click “copy” with “P1” (Program 1) gain settings visible.

(2) Click on “P2” and click “paste”.

(a) Select “all” gain values on “P2” frequency gain response and decrease overall gain by appropriate amount based on HFPTA chart above.

(3) Click on “P3” and click “paste”.

(a) Select “all” gain values on “P3” frequency gain response and decrease overall gain by appropriate amount based on HFPTA chart above.

(4) Click on “P4” and click “paste”.

(a) Select “all” gain values on “P4” frequency gain response and increase overall gain by appropriate amount based on HFPTA chart above.

(5) Before removing the hearing aids, review “beeps” with the participant by clicking on “Beeps and Volume Control” and presenting each beep sample (delayed start, low battery, and program change) and verifying that the beeps are loud enough.

(6) Click “Save” to save settings to hearing aids and to Aventa software (click “No” if a box pops up that prompts you to “use auto relate to apply gain adjustments from one program to the other programs”).

h. Real ear insertion gain using Verifit

(1) “Tests”→ “On-ear measures”→”Insertion gain” (65 dB SPL pure tones)

(2) Remove hearing aids, keeping probe tube inserted in ear canals.

(3) Select “Audiogram” and change “REUR” to “Measure”.

(4) Measure REUR for both ears (probe tubes are the only things in ears).

(5) After REUR is measured in both ears, insert hearing aids and measure REAR using a 65 dB SPL tonal sweep for both ears.

(6) Change format settings to “Table”.

(7) Select “Print” and enter file name and press “Continue” to save to USB flash drive.

(8) Disinfect probe tubes with two disinfectant wipes, dry with paper towel, and place in participant’s envelope.

i. Final coupler measurements using Verifit

(1) Run test box speechmap on hearing instruments following programming (right, then left) (open/close battery door following programming to disconnect from Aventa).

(2) Audiograms and the RECD should still be entered from real ear measurements.

(3) “Tests”→ “Test box measures”→ “Speechmap”

(4) Couple hearing aid to ITE coupler using new strip of sticky tack (this will be used for their hearing aids only) keeping dome in place.

   (a) Run speechmap for “carrot passage” at 65, 55 and 75 dB SPL input.

   (b) Run MPO test.

   (c) Record SII values for 65, 55, and 75 dB SPL stimuli on Session 2 Audiologist Checklist (2-1).

   (d) Repeat with left hearing aid.

(5) Change format settings to “Table”.

(6) Select “Print”, enter file name, and press “Continue” to save to USB flash drive.

(7) Save as an XML file

   (a) Press “Session” on Verifit, then select “Store session to file”.

   (b) Select the “Client ID” box and enter file name and press “Continue”. This will save file in “Sessions” folder on USB flash drive along with a style sheet (audioscan.xsl).

(8) Discard sticky tack.

(9) Record hearing aid serial number, color, tubing and dome sizes on light blue Hearing Aid Decision Form for Audiologist (2-2).

(10) Draw shade to block the view of the participant side of the booth.

(11) Take participant to tester side of booth and tell him or her that A3 will be in shortly to complete the hearing aid orientation and answer any questions about hearing aid maintenance.

(12) Leave hearing aids (without batteries inserted) in participant's hard case on tester side of the booth for A3.

(13) Take participant's folder to CTC.

(14) Notify A3 that participant is ready for orientation. Participant's guest may join him and her at this time (A3 will escort guest if applicable).

(15) A1/A2 will transfer all (renamed) Verifit files from USB flash drive onto server in appropriate folders. Also transfer same files on USB flash drive into "USB dump file" on server.

(16) Print the testbox speechmap for both ears and place in the Session 2 folder.

j. Hearing aid orientation

(1) A3 completes hearing aid orientation (~45-60 minutes) covering each point on Orientation Checklist (2-3).

(2) Inform participant that any additional questions that come up during the six-week trial period need to be directed to CTC.

(3) A3 escorts participant to CTC's office after orientation is completed.

k. CTC does the following

(1) Gives participant 8.35% discount, and collects full payment for hearing aids.

(2) Refers to light blue Hearing Aid Decision Form for Audiologist (2-2) to determine which size of extra domes to place in participant's hearing aid kit.

(3) Records participant's hearing aid information (serial numbers, warranty info,

battery size, etc.) in the User Guide (2-4) on the inside of the front cover.

(4) Provides participant with hearing aid kit containing batteries, cleaning tools, extra domes, User Guide, etc.

(5) Opens hearing aid kit to verify presence of all required items. Checks these items on the Hearing Aid Case Checklist for CTC (2-5). If the participant has any questions regarding contents, CTC will tell the participant to refer to the User Guide.

(6) Schedules participant's Session 3 appointment time for six weeks (±one week) after Session 2, providing him or her with parking pass and reminder card with the CTC's contact number.

(7) Emphasizes the importance of calling CTC if questions or problems arise, and escorts participant out of the building.

5. Placebo group hearing aid selection and fitting

a. If participant is one of the 20% that are designated tulip-dome fittings, the post-it will read ("P Tulip") letting A1/A2 know that a tulip dome should be used with this participant instead of an open dome. (NOTE: given 0-dB insertion gain, a tulip dome would most likely never be needed by placebos; therefore, the presence of a tulip dome could potentially tip off the blinded audiologist regarding group assignment. This led to use of 20% tulip domes for placebos.)

b. A1/A2 escorts participant to participant side of audio booth.

c. Turn chair and place within marks on the floor and position the external speaker within the marks on the floor for real-ear testing. Participant is seated in this chair for hearing aid programming.

d. Hearing aid color and dome choice

(1) Participant chooses hearing aid color. Audiologist chooses appropriate dome size and tubing length using hearing aid manufacturer's tubing measurement tool.

e. Program hearing aids

(1) Audiologist places Audiogram (1-6) on lap to be in view of the participant to mimic what occurs in typical audiology clinic.

(2) Enter participant number and audiogram into Noah, then click Aventa in upper right corner.

(3) Connect hearing aid and generate first fit in the manufacturer's fitting software using NAL-NL2 fitting algorithm.

(4) Fitting should default to "Experienced Non-Linear".

(5) Select "Fitting" tab (top toolbar) and turn "Binaural Correction" off.

(6) Change "Physical Properties" to the size of tube and dome chosen.

(7) Change P1 to "Basic+Softswitching" if it is not already on that setting.

(a) Directionality: Fixed (Omni as of 5/5/2014)

(b) Expansion: Off

(c) Noise Tracker II: Per Environment

(d) Wind Guard: Off

(8) Click on "Environmental Optimizer" and click "Reset to…" then select "0 dB" so that all levels are at zero.

(9) If applicable, click on "Tinnitus Sound Generator" and ensure it is off.

f. Real-ear insertion gain using Verifit

(1) Prior to completing Verifit tests, enter zeros for audiogram thresholds and enter

participant’s measured RECD into Verifit.

(2) “Tests”→ “On-ear measures”→”Insertion Gain” (65-dB-SPL pure tones)

(3) Insert one probe tube in each ear.

(a) Check that the probe tube is connected to the appropriate right or left cord.

(b) Check that each reference microphone is facing outwards.

(4) Select “Audiogram” and change “REUR” to “measure”.

(5) Measure REUR for both ears (probe tubes are only things in ears).

(6) After REUR is measured in both ears, insert hearing aids and measure REAR using a 65 dB SPL tonal sweep for both ears.

(7) Fine tune gain in software to make REIG response as close to zero as possible.

(8) If REIG is too high: Use “Environmental Optimizer” settings to reduce REIG (these settings must change equally for all environments and can only be reduced down to a maximum of 6 dB).

(9) If REIG is too low: use gain adjustment to increase gain at specific frequencies.

(10) Change format settings to “Table”.

(11) Insert USB flash drive into Verifit USB port.

(a) Select “Print” and enter file name and press “Continue” to save to USB flash drive.

(12) Disinfect both probe tubes with two disinfectant wipes, dry with paper towel, and place in envelope with participant # labeled on front.

g. Program VC

(1) Click “copy” with “P1” (Program 1) gain settings visible.

(2) Click on “P2” and click “paste”.

(a) Select “all” gain values on “P2” frequency gain response and increase overall gain +1 dB.

(3) Click on “P3” and click “paste”.

(a) Do not make any gain changes to this program.

(4) Click on “P4” and click “paste”.

(a) Select “all” gain values on “P4” frequency gain response and increase overall gain by +3 dB.

(5) Return to “P1”.

(a) Select “all” gain values on “P1” frequency gain response and increase overall gain +2 dB.

(6) Before removing hearing aids, review “beeps” with participant by clicking on “Beeps and Volume Control” and presenting each beep sample (delayed start, low battery, and program change) and verifying that beeps are loud enough.

(7) Click “Save” to save settings to hearing aids and to Aventa software (click “No” if a box pops up that prompts you to “use auto relate to apply gain adjustments from one program to the other programs”).

h. Final coupler measurements

(1) Run test box speechmap with “carrot passage” input on hearing instruments following programming (right, then left) (open/close battery door following programming to disconnect from Aventa).

(2) Audiogram and the RECD should still be entered from real ear measurements.

(3) “Tests”→ “Test box measures”→ “Speechmap”

(4) Couple hearing aid to ITE coupler using new strip of sticky tack (this will be

used for their hearing aids only) keeping dome in place.

(a) Run speech at 65, 55 and 75 dB SPL input.

(b) Run MPO test.

(c) Record SII values for 65, 55, and 75 dB SPL stimuli on the Session 2 Audiologist Checklist (2-1).

(d) Repeat with left hearing aid.

(5) Change format settings to “Table”.

(6) Select “Print” and enter file name and press “Continue” to save to USB flash drive.

(7) Save as an XML file:

(a) Press “Session” on Verifit then select “Store session file”.

(b) Select the “Client ID” box and enter file name and press “Continue”. This will save file in “Sessions” folder on USB stick along with a style sheet (audioscan.xsl).

(8) Discard sticky tack.

(9) Record hearing aid serial number, color, tubing and dome sizes on light blue Hearing Aid Decision Form for Audiologist (2-2).

(10) Draw shade to block the view of the participant side of the booth.

(11) Take participant to testing side of booth and tell the participant that A3 will be in shortly to complete the hearing aid orientation and answer any questions about hearing aid maintenance.

(12) Leave hearing aids (without batteries inserted) in participant’s hard case on tester side of the booth for A3.

(13) Take participant's folder to CTC.

(14) Notify A3 that participant is ready for orientation. Participant's guest may join him or her at this time (A3 will escort the guest if applicable).

(15) A1/A2 will transfer all (renamed) Verifit files from USB flash drive onto server in appropriate folders. Also transfer same files on USB flash drive into "USB dump file" on server.

(16) Print the test box speechmap for both ears and place in the Session 2 folder.

i. Hearing aid orientation (2-3)

(1) A3 completes hearing aid orientation (~45-60 minutes) covering each point on Orientation Checklist (2-3).

(2) Inform participant that any additional questions that come up during the six-week trial period need to be directed to CTC.

(3) A3 escorts participant to CTC's office after orientation is completed.

j. CTC does the following:

(1) Gives participant 8.35% discount, and collects full payment for hearing aids at this time.

(2) Refers to light blue Hearing Aid Decision Form for Audiologist (2-2) to determine which size of extra domes to place in participant's hearing aid kit.

(3) Records participant's hearing aid information (serial numbers, warranty info, battery size, etc.) in the User Guide (2-4) on the inside of the front cover.

(4) Provides participant with hearing aid kit containing batteries, cleaning tools, extra domes, User Guide, etc.

(5) Opens hearing aid kit to verify presence of all required items. CTC checks these

items on the Hearing Aid Case Checklist for CTC (2-5). If the participant has any questions regarding contents, CTC will tell the participant to refer to the User Guide (2-4).

(6) Schedules participant’s Session 3 appointment time for six weeks (±one week) after Session 2, providing him or her with parking pass and reminder card with the CTC’s contact number.

(7) Emphasizes the importance of calling CTC if questions or problems arise, and escorts participant out of building.

6. CD group hearing aid selection

a. CTC sets up Push and Play tablet.

(1) Double tap “start” → “My Computer”→ “python” → “push and play”

(2) Enter participant number.

b. CTC escorts the participant to the Clinical Trial Hearing Aid Room equipped with:

(1) CD Rx Form (2-6) for recording hearing aid choice

(2) Two mirrors

(3) Tablet (HP slate 2) for speech, music, and environmental sounds demos

(4) Tablet (Samsung Series 7 Slate) for introduction video (see supplemental materials)

(5) Loud speaker mounted to wall directly in front of participant

(6) Three partitioned bins (beige bin, brown bin, and grey bin) each with two X hearing aids, two Y hearing aids, and two Z hearing aids [programmed according to section #9 of the New Hearing Aid Check-in Protocol (M-2)] in each partition

(7) Two bins (left/right) with four partitions for slim tube choices (0B, 1B, 2B, 3B)

(8) One bin with four partitions for dome choices (S, M, L, tulip)

(9) Black bin labeled “No” for pieces that were tried, but unwanted

(10) Three instruction binders [black (2-7), red (2-8), blue (2-9)]

c. CTC starts the introduction video on the Samsung tablet for the participant.

d. When the video is over, CTC gets the Samsung tablet from the participant, and reminds him or her that the participant should begin choosing their hearing aid by reading through the binders [starting with the black binder (2-7)].

e. CTC instructs participant to press button on pager system when he or she has made a hearing aid selection for both ears then leaves Clinical Trials Hearing Aid Room.

f. Participant chooses hearing aid (2-7), (2-8), (2-9)

(1) Participant selects which aid (X, Y, Z), color (beige, brown, grey), tubing (0B, 1B, 2B, 3B), and tip (S, M, L, tulip) by reading the black (2-7), red (2-8) and blue (2-9) binders, then pages CTC.

g. CTC re-enters CD room and closes the Push and Play by tapping “X” in the upper right corner and prints the results.

(1) CTC records details of the unwanted hearing aids, tubes, and/or domes in the black bin and records this on the Hearing Aid Decision Form for CTC (2-10).

h. CTC escorts participant (and guest) into the CTC office and does the following:

(1) Reviews what the participant filled out on the Rx Form (2-6), and records hearing aid models, serial numbers, slim tube size and dome size on Hearing Aid Decision Form for CTC (2-10)

(2) Verifies the serial numbers of the selected hearing aids match up with the

appropriate X, Y, or Z programming indicated on the CD check-in chart

(3) Records dates and initials next to each hearing aid purchased on the CD check-in chart

(4) Removes the sticker on hearing aid signifying “X”, “Y”, or “Z”

(5) Marks the right hearing aid with the red indicator and the left hearing aid with the blue indicator and explains to participant what these colors indicate

(6) Gives participant 8.35% discount, and collects full payment for hearing aids

(7) Refers to light blue CD Rx Form (2-6) to determine which size of extra domes to place in participant’s hearing aid kit

(8) Records participant’s hearing aid information (serial numbers, warranty info, battery size, etc.) in the User Guide (2-4) on the inside of the front cover

(9) Provides participant with hearing aid kit containing batteries, cleaning tools, extra domes, User Guide, etc.

(10) Opens hearing aid kit to verify presence of all required items, checks these items on the Hearing Aid Case Checklist for CTC (2-5). If the participant has any questions regarding contents, CTC will tell him or her to refer to the User Guide (2-4).

(11) Schedules participant’s Session 3 appointment time for six weeks (±one week) after Session 2, providing the participant with parking pass and reminder card with the CTC’s contact number

(12) Emphasizes the importance of calling CTC if questions or problems arise, and escorts participant out of building

D. UNSCHEDULED VISITS (ALL GROUPS) (M-1)

1. If participant contacts CTC following Session 2 reporting hearing aid problem(s), the following steps will be followed:
   a. CTC will ask him or her to first review their User Guide (2-4) for assistance.
   b. If the problem persists, and it is just for one hearing aid, have the participant remove both hearing aids and compare the functioning hearing aid to the non-functioning hearing aid in terms of battery, tubing, and dome or tip (e.g., plugged with wax?).
   c. If participant cannot resolve issue, participant will schedule appointment with CTC at earliest convenience.
   d. Unscheduled visit:
      (1) CTC will inspect hearing aid using the Unscheduled Visit Checklist (M-1) for obvious problems (one checklist per hearing aid).
      (2) If CTC is not able to resolve issue, participant will wait in the waiting room while CTC consults with the audiologist who saw the participant during Session 1 (if possible).
         (a) CTC will tell participant: "I need to run some checks on the hearing aid, I will be right back." CTC will not tell the participant that there is an audiologist involved- regardless of group.
      (3) Audiologist assists CTC in returning hearing aid to previous functioning.
      (4) If no problem in hearing aid function is discovered, CTC will re-introduce participant to audiologist from Session 1. Audiologist will take the participant to the booth area for otoscopy, tympanometry, etc. as needed to attempt to determine underlying issue.

(5) CTC reminds participant of next appointment date before he or she leaves.

e. Log all inquiries, noting the problem, additional unscheduled visits (if needed), and the final resolution.

III. SESSION 3 – ARL: 90-150 MINUTES REQUIRED

Study personnel

A1: Audiologist 1

A2: Audiologist 2

A4: Audiologist 4

CTC: Clinical Trials Coordinator

Components

A. CTC-PARTICIPANT ARRIVAL
B. A1/A2-AS-WORN AIDED CST FORM (3-1)
C. A1/A2-PHYSICAL INSPECTION OF HEARING AIDS (3-2)
D. A1/A2-AS-WORN SIMULATED SPEECHMAP (3-2)
E. A1/A2- HEARING AID MAINTENANCE (3-2)
F. A4- AIDED PHAP (T-3), AIDED HHIE (T-4) AND HASS (T-5)
G. A4- POST-MAINTENANCE AIDED CST FORM (3-3)
H. A4- PHAST-R (T-6)
I. A4-MUSIC/TRAINING QUESTIONNAIRE (3-4)
J. A4- FINAL HEARING AID DECISION FORM #1 (3-5)
K. A4- REVEAL GROUP DESIGNATIONS (3-5)
L. A4- DATA LOGGING FORM (3-6)
M. A4- PROGRAMMING AND/OR ADJUSTMENT OF HEARING AIDS (3-7)
N. A4- HEARING AID ADJUSTMENT LOG (3-8)
O. A4- HEARING AID QUESTIONS FORM (3-9)
P. A4- HEARING AID STATUS FORM #1 (3-10)

Q. CTC- SCHEDULE FOLLOW-UP

<u>Forms/Materials Needed:</u> As-Worn Aided CST Form (3-1), Hearing Aid Inspection Checklist (3-2), Tablet for Aided PHAP (T-3), Aided HHIE (T-4), HASS (T-5), and PHAST-R (T-6), Post-Maintenance Aided CST Form (3-3), Music/Training Questionnaire (3-4), Final Hearing Aid Decision Form #1 (3-5), Data Logging Form (3-6), Session 3 Checklist (3-7), Hearing Aid Adjustment Log (3-8), Hearing Aid Questions Form (3-9), Hearing Aid Status Form #1 (3-10), Final Exit Form #2 (3-11, if Session 3B not needed), Orientation Checklist if needed (2-3), Audiogram (1-6), RECD, and envelope with probe tubes from Session 2.

A. PARTICIPANT ARRIVAL

1. Participant arrives at CTC's office.
2. CTC takes him or her to the waiting room and pages A1/A2 (whoever did not work with the participant during Sessions 1 and 2).
3. CTC provides A1/A2 with participant's Session 3 folder containing the RECD, Audiogram (1-6), and envelope containing the participant's probe tubes as well as other needed forms.

B. AS-WORN AIDED CST FORM (3-1)

1. A1/A2 escorts participant to the ARL.
2. Administer the as-worn aided CST (3-1)
    a. If participant is not wearing hearing aids, instruct him or her to put on the hearing aids.
    b. Scoring sheet will be in tester side of booth.
    c. Seat participant in designated chair (make sure chair is located in correct position on floor).

d. Audiometer settings:

   (1) Track 1: Calibrate—complete prior to every test

   (2) Channel 1: 50 dB HL, External A, Left (front speaker), Speaker

   (3) Channel 2: 46 dB HL, External B, Right (back speaker), Speaker

e. Instructions: “You will hear a woman talking from in front of you. There will be noise behind you. Ignore the background noise, and try to repeat back what the woman says. She will say one sentence at a time. Each group of sentences has a topic. The first topic is “umbrella”. Do you have any questions?”

f. Present CST (track 33) at 50 dB HL from loudspeaker located at 0˚ azimuth and elevation with the multi-talker babble from the CST presented at 46 dB HL (+3 dB signal-to-babble ratio) from an identical loudspeaker located at 180˚ azimuth and 0˚ elevation.

g. Have participant complete two passage pairs (“umbrella” and “giraffe”; 50 total key words). Provide participant with second passage topic prior to starting second passage.

h. Present sentences one at a time, pausing the CD after each sentence to score.

i. Key words are capitalized and bolded on the scoring sheet. To score, cross out key words that are repeated incorrectly or are not repeated at all.

j. Participant must repeat the word exactly as it is to get it correct. Any other variation of the word, incorrect word, or absence of the word should be marked incorrect.

k. Score = total number of correct words

3. Draw shade to cover window so that A4, who will sit on tester side while participant does surveys on tablet, can’t see what A1/A2 are doing during hearing aid inspection.

4. Seat participant at tympanometry table and remove hearing aids to complete otoscopy and record on Hearing Aid Inspection Checklist (3-2).
    a. Bring otoscope to participant side of audio booth to assist with hearing aid inspection.
5. Page A4 using one button pager to notify A4 that participant is ready to complete surveys on tablet (see F below) while A1/A2 complete steps C-E.

C. PHYSICAL INSPECTION OF HEARING AIDS (3-2)

1. A1/A2 completes Hearing Aid Inspection Checklist (3-2).
    a. Do not open battery door until after electroacoustic exam is complete.
2. Otoscopy (status of EAC and pinna should be recorded on checklist)
3. Physical examination of as-worn hearing aids
    a. Visual inspection of ear tips or tubing to check for breaks or cerumen occlusion
    b. Record on the checklist.

D. AS-WORN SIMULATED SPEECHMAP (RIGHT, THEN LEFT) (3-2)

1. Couple hearing aid to ITE coupler using new sticky tack.
2. On the Verifit, “Test Box Measures” → “Speechmap” (“carrot passage”)
3. Enter participant’s RECD and audiogram.
4. Run speechmap at 55, 65 and 75 dB SPL.
5. Run MPO test.
6. Record SIIs for 65, 55, and 75 dB SPL stimuli on the Hearing Aid Inspection Checklist (3-2).
7. Change format settings to “Table”.
8. Insert USB into Verifit.

9. Select “Print” and enter file name and press “Continue” to print to USB.
10. Repeat with the left hearing aid.
11. Save as an XML file:
    a. Press “Session” on Verifit, then select “Store session file”.
    b. Select the “Client ID” box and enter file name and press “Continue” (refer to file naming nomenclature sheet in booth). This will save file in “Sessions” folder on USB stick along with a style sheet (audioscan.xsl).
12. Listening check
    a. Record the program hearing aids are in on checklist.
    b. Record any abnormalities in hearing aid sound quality on checklist.
    c. Record any hearing device deficiencies on checklist.
13. Open battery door and record battery condition on checklist (record action to fix battery condition on checklist if necessary).

E. HEARING AID MAINTENANCE (3-2)

1. Complete remaining portions of Hearing Aid Inspection Checklist (3-2) and record any maintenance performed.
2. Post-maintenance test box measures (same as was done on as-worn hearing aids)
    a. Always re-run in test box even if no maintenance was needed.
3. Leave sticky tack on coupler for A4.
4. Record post-maintenance SIIs for 65, 55, and 75 dB SPL stimuli on Hearing Aid Inspection Checklist.
5. Select “Print” and enter file name and press “Continue” to print to USB.
6. Save as an XML file:

a. Press “Session” on Verifit, then select “Store session file”.
b. Select the “Client ID” box and enter file name and press “Continue” (refer to file naming nomenclature sheet in booth). This will save file in “Sessions” folder on USB stick along with a style sheet (audioscan.xsl).

7. Give hearing aids to A4 with battery doors open.
8. Print the post-maintenance simulated speechmap for both ears and give it to CTC to along with the As-Worn CST Form (3-1) and Hearing Aid Inspection Checklist to be placed in participant’s file.

F. AIDED PHAP (T-3), AIDED HHIE (T-4) AND HASS (HEARING AID SATISFACTION SURVEY) (T-5)

1. A4 will have participant follow instructions and complete the surveys on the tablet. Participant will page A4 with one-button pager when finished with each survey and A4 will start the next survey until all three surveys are complete.

G. POST-MAINTENANCE AIDED CST FORM (3-3)

1. A4 instructs participant to sit in designated chair (make sure chair is located in correct position on floor).
2. A4 places participant’s hearing aids in ears.
3. Audiometer settings:
   a. Track 1: Calibrate (already completed by A1/A2)
   b. Channel 1: 50 dB HL, External A, Left (front speaker), Speaker
   c. Channel 2: 46 dB HL, External B, Right (back speaker), speaker
4. Instructions: “You will hear a woman talking from in front of you. There will be noise behind you. Ignore the background noise, and try to repeat back what the woman says.

She will say one sentence at a time. Each group of sentences has a topic. The first topic is “nail”. Do you have any questions?”

5. Present CST (track 33) at 50 dB HL from loudspeaker located at 0˚ azimuth and elevation with the multi-talker babble from the CST presented at 46 dB HL (+3 dB signal-to-babble ratio) from an identical loudspeaker located at 180˚ azimuth and 0˚ elevation.
6. Have participant complete two passage pairs (“nail” and “woodpecker”; 50 total key words). Provide participant with second passage topic prior to starting second passage.
7. Present sentences one at a time, pausing the CD after each sentence to score.
8. Key words are capitalized and bolded on the scoring sheet. To score, cross out key words that are repeated incorrectly or are not repeated at all.
9. Participant must repeat the word exactly as it is to get it correct. Any other variation of the word, incorrect word, or absence of the word should be marked incorrect.
10. Score = total number of correct words

H. PHAST-R (PRACTICAL HEARING AID SKILLS TEST-REVISED) (T-6)

1. A4 escorts the participant to the tester side of the booth.
2. Instructions: Place the following items in front of the participant: A telephone, different size batteries (10, 312, 13, 675), magnetic tool for battery removal, cleaning tools: brush, cloth, wax loop, plastic “wire” tube cleaner.
3. Use PHAST-R (T-6) score sheet on tablet to rate participant’s performance on each item.

I. MUSIC/TRAINING QUESTIONNAIRE (3-4)

1. Administer Music/Training Questionnaire (3-4).

J. FINAL HEARING AID DECISION FORM #1 (3-5)

1. A4 will now ask, “Based on your experience over the past 6 weeks, are you going to keep the aids?”, and record response on form 3-5.
    a. If the participant responds “no”, ask “why?” Record response on Final Hearing Aid Decision Form #1 (3-5)
    b. Regardless of participant’s response, proceed with 2.
2. A4 will take Tablet to CTC who will then reveal participant’s group to A4.
3. CTC will transfer tablet questionnaire results to appropriate folder on server. CTC will also print the output files from the tablet and place into participant’s Session 3 folder.
4. Once results are printed and files have been copied to the server, CTC will delete the files from the USB and the tablet.

K. REVEAL GROUP DESIGNATIONS (3-5)

1. Purchase price is not discussed at this time.
2. Regardless of participant response about keeping hearing aids, A4 will reveal the assigned group to the participant using the descriptions on form 3-5. A4 will then proceed with data logging and then either run DFS (digital feedback suppression) and reprogram hearing aids, or accompany participant to CTC for a refund.

L. DATA LOGGING FORM (3-6)

1. Regardless of participant group, A4 will connect hearing aids to software and view data logging and record on Data Logging Form (3-6).
    a. Print data logging screen.
    b. Reset data logging.
3. If participant elects to keep hearing aids and wants them re-programmed or adjusted, see “M”.

a. A program swap is not considered to be a reprogramming/adjustment.

4. If participant elects to keep hearing aids but doesn't want them re-programmed:

   a. Run DFS.

   b. Complete simulated speechmap measurements in test box for 65, 55, 75 dB SPL and MPO.

      (1) If programs have been swapped, simulated speechmaps should be run with hearing aids in former program 1.

   c. Record SIIs for 65, 55, and 75 dB SPL stimuli on Session 3 Checklist (3-7).

   d. Discard sticky tack.

   e. Save coupler results:

      (1) Change format settings to "Table".

      (2) Insert USB into Verifit.

         (a) Select "Print" and enter file name and press "Continue" to print to USB for both ears.

         (b) Save as an XML file (refer to file naming nomenclature list in booth) and print to USB following directions in as-worn simulated speechmap section.

   f. Fill out Exit Form #2 (3-11).

   g. Skip to step N below.

5. If participant elects to return hearing aids, take participant to CTC to get refund and IU Hearing Clinic card.

   a. CTC will complete process for returned hearing aids.

   b. CTC or A4 will fill out Exit Form #2 (3-11).

6. These decisions will be recorded on Hearing Aid Status Form #1 (3-10).

M. PROGRAMMING AND/OR ADJUSTMENT OF HEARING AIDS (3-7)

1. If DFS must be run in addition to programming changes, it should be completed in the following order:
   a. Programming changes
   b. Run and save post-adjustment simulated speechmaps in testbox.
   c. Run DFS.
   d. Run and save post-DFS simulated speechmaps in testbox.
      (1) If this order cannot be maintained, note this in screenshot and XML file names so that data will not be used.
2. AB group (not including Placebo)
   a. Complete any fine-tuning that the participant requests based on participant feedback. A program swap is not considered fine-tuning/adjustment.
      (1) Ensure RECD and audio are entered in Verifit.
   b. Run real-ear speechmap for "carrot passage" for both ears at 65, 55, and 75 dB SPL and MPO at 85 dB SPL.
   c. Record SIIs for 65, 55, and 75 dB SPL stimuli on Session 3 Checklist (3-7).
   d. Discard sticky tack.
   e. Change format settings to "Table".
   f. Select "Print" and enter file name and press "Continue" to print to USB.
      (1) Save as an XML file.
         (a) Press "Session" on Verifit, then select "Store session file".
         (b) Select the "Client ID" box and enter file name and press "Continue" (refer to file naming nomenclature sheet in booth). This will save the file in "Sessions" folder on USB stick along with a style sheet (audioscan.xsl).
3. Placebo and CD groups

a. Reprogram hearing aids in accordance with NAL-NL2 prescription procedure.

   (1) Ensure participant's RECD and audio are entered in to the Verifit.

b. Use Verifit in the sound booth to run real ear speechmap at 65 dB SPL for "carrot passage" to verify target gain (NAL-NL2).

   (1) Adjust gain in Aventa as needed until targets are matched appropriately.

   (2) Target-Matching Rule: For 250 through 4000 Hz, all measured values within 4 dB of target at all frequencies in this range is the goal, but will accept fit after initial fine-tuning attempts if only within 7 dB of target at all frequencies in this range.

c. Run speechmap at 55 dB SPL and 75 dB SPL.

d. Record SIIs for 65, 55, and 75 dB SPL stimuli on Session 3 Checklist (3-7).

e. Run 85 dB SPL pure tone sweep (MPO) stimulus to verify maximum output isn't uncomfortably loud/painful.

   (1) Re-familiarize participant with loudness categories and instruct:

   (2) "You will hear several loud beeps that get higher in pitch. The beeps will be loud, but if it gets uncomfortably loud [refer to Categories of Loudness (1-8)], raise your hand. It is very quick so try to raise your hand as quickly as possible then put it down and continue to listen. We can repeat if necessary."

   (3) Run the MPO 85 dB SPL stimulus with the hearing aid on in right ear only. Left ear is muted.

   (4) If Participant raises hand, decrease maximum output at corresponding frequency by 4 dB, record on Session 3 Checklist Form (3-7), and re-initiate 85 dB SPL stimuli.

(5) Continue until “uncomfortably loud” responses are no longer obtained.

(6) Repeat with left hearing aid on and right hearing aid muted.

(7) Finally, turn both hearing aids on and run the MPO stimulus. If participant reports none of the sounds are “uncomfortably loud” move on to programming the volume control (see f. below). If participant reports one of the sounds uncomfortably loud, use the procedure above to determine which frequency to lower by 2 dB in both hearing aids until “uncomfortably loud” response is no longer obtained. Record any adjustments on Session 3 Checklist Form.

f. Program VC based on HFPTA-use chart below for amounts

(1) Click “Copy” P1.

(2) Click on “P2”, and click “paste”.

(a) Click “all” on P2 frequency gain response and decrease overall gain by appropriate amount for their HFPTA.

(3) Click on “P3” and click “paste”.

(a) Click “all” on P3 frequency gain response and decrease overall gain by appropriate amount for their HFPTA.

(4) Click on “P4” and click “paste”

(a) Click “all” on P4 frequency gain response and increase overall gain by appropriate amount for their HFPTA.

(5) Click “Save” to save settings to hearing aids and to Aventa software (click “No” if a box pops up that prompts you to “use auto relate to apply gain adjustments from one program to the other programs”).

HFPTA (1,2, and 4kHz)	VC P1	VC P2	VC P3	VC P4
≤21	0	-2	-4	2
22-29	0	-3	-6	3
30-38	0	-4	-8	4
39-47	0	-5	-10	5
48-55	0	-6	-12	6
56-64	0	-7	-14	7
≥ 65	0	-8	-16	8

g. Final coupler measurements and orientation if needed (2-3)

(1) Run test box simulated speechmap following programming.

(2) Select “Print” and enter file name and press “Continue” to print to USB.

(3) Save as an XML file:

(a) Press “Session” on Verifit then select “Store session file”.

(b) Select the “Client ID” box, enter file name, and press “Continue” (refer to file naming nomenclature sheet in booth). This will save file in “Sessions” folder on USB stick along with a style sheet (audioscan.xsl).

(4) Inform participant that the 4-week money-back trial period will start anew at the end of this session.

(5) CD group

(a) Complete hearing aid orientation and counseling using Orientation Checklist (2-3) as a guide.

N. HEARING AID ADJUSTMENT LOG (3-8)

1. Record any adjustments made to the hearing aids.

O. HEARING AID QUESTIONS FORM (3-9)

1. Record all questions the participant asked during the course of Session 3.

P. HEARING AID STATUS FORM #1 (3-10)

1. Document if the participant returned, kept as-is, or requested reprogramming of the hearing aids.

Q. SCHEDULE FOLLOW-UP

1. Escort participant to CTC's office.
2. Provide 8.35% refund.
3. CTC schedules follow-up appointment.
    a. Those who did not get re-programming or adjustments
        (1) Schedules a 1-year follow-up appointment and gives participant card to call for questions or adjustment appointments as needed.
        (2) Gives the participant a dri-aid kit and provides instruction on its use.
        (3) Fills out Exit Form #2 (3-11)
    b. Those who received adjustments or re-programming:
        (1) Follow-up appointment will be scheduled for four weeks after Session 3.

IV. SESSION 3B – ARL: 60-90 MINUTES REQUIRED

Study personnel

A1: Audiologist 1

A2: Audiologist 2

A3: Audiologist 3

A4: Audiologist 4

CTC: Clinical Trials Coordinator

Components

A. CTC- PARTICIPANT ARRIVAL
B. A1/A2/A3/A4- AS-WORN AIDED CST FORM (3B-1)
C. A1/A2/A3/A4- PHYSICAL INSPECTION OF HEARING AIDS (3B-2)
D. A1/A2/A3/A4- AS-WORN SIMULATED SPEECHMAP (3B-2)
E. A1/A2/A3/A4- HEARING AID MAINTENANCE (3B-2)
F. A1/A2/A3/A4- AIDED PHAP (T-3) AIDED HHIE (T-4) AND HASS (T-5)
G. A1/A2/A3/A4- POST-MAINTENANCE AIDED CST FORM (3B-3)
H. A1/A2/A3/A4- PHAST-R (T-6)
I. A1/A2/A3/A4- MUSIC/TRAINING QUESTIONNAIRE (3B-4)
J. A1/A2/A3/A4- HEARING AID DECISION FORM #2 (3B-5)
K. A1/A2/A3/A4- DATA LOGGING FORM (3B-6)
L. A1/A2/A3/A4- ADJUSTMENT OF HEARING AIDS (3B-7)
M. A1/A2/A3/A4- HEARING AID ADJUSTMENT LOG (3B-8)
N. A1/A2/A3/A4- HEARING AID QUESTIONS FORM (3B-9)
O. A1/A2/A3/A4- HEARING AID STATUS FORM #2 (3B-10)

P. CTC- SCHEDULING

Forms/Materials Needed: As-Worn Aided CST Form (3B-1), Hearing Aid Inspection Checklist (3B-2), Post-Maintenance Aided CST Form (3B-3), Tablet for Aided PHAP (T-3), Aided HHIE (T-4), HASS (T-5), and PHAST-R (T-6), Music /Training Questionnaire (3B-4), Hearing Aid Decision Form #2 (3B-5), Data Logging Form (3B-6), Session 3B Checklist (3B-7), Hearing Aid Adjustment Log (3B-8), Hearing Aid Questions Form (3B-9), Hearing Aid Status Form #2 (3B-10), Exit Form #2 (3-11), Audiogram (1-6), RECD, and envelope with probe tubes from Session 3

A. PARTICIPANT ARRIVAL

1. Participant arrives at CTC's office.
2. CTC takes him or her to the waiting room and pages A1/A2/A3/A4.
3. CTC provides A1/A2/A3/A4 with participant's Session 3B folder containing the RECD, Audiogram (1-6), envelope containing the participant's probe tubes, and other needed forms.

B. AS-WORN AIDED CST FORM (3B-1)

1. A1/A2/A3/A4 escort participant to ARL.
2. Administer the as-worn aided CST (3B-1).
    a. If participant is not wearing hearing aids, instruct him or her to put on the hearing aids.
    b. Scoring sheet (3B-1) will be in tester side of booth.
    c. Instruct participant to sit in designated chair (make sure chair is located in correct position on floor).
    d. Audiometer settings:

(1) Track 1: Calibrate—complete prior to every test

(2) Channel 1: 50 dB HL, External A, Left (front speaker), Speaker

(3) Channel 2: 46 dB HL, External B, Right (back speaker), speaker

e. Instructions: “You will hear a woman talking from in front of you. There will be noise behind you. Ignore the background noise, and try to repeat back what the woman says. She will say one sentence at a time. Each group of sentences has a topic. The first topic is “donkey”. Do you have any questions?”

f. Present CST (track 40) at 50 dB HL from loudspeaker located at 0˚ azimuth and elevation with the multitalker babble from the CST presented at 46 dB HL (+3 dB signal-to-babble ratio) from an identical loudspeaker located at 180˚ azimuth and 0˚ elevation.

g. Have participant complete two passage pairs (“donkey” and “guitar”; 50 total key words). Provide participant with second passage topic prior to starting the second passage.

h. Present sentences one at a time, pausing the CD after each sentence to score.

i. Key words are capitalized and bolded on the scoring sheet. To score, cross out key words that are repeated incorrectly or are not repeated at all.

j. Participant must repeat the word exactly as it is to get it correct. Any other variation of the word, incorrect word, or absence of the word should be marked incorrect.

k. Score = total number of correct words

3. Seat participant at tympanometry table and remove hearing aids to complete otoscopy [record on Hearing Aid Inspection Checklist (3B-2)].

   a. Bring otoscope to participant side of audio booth to assist with hearing aid

inspection.

4. If another audiologist is administering surveys, page that audiologist using the one button pager to notify the other audiologist that participant is ready to complete surveys on tablet (step F) while steps C-E are completed.

C. PHYSICAL INSPECTION OF HEARING AIDS (3B-2)

1. Complete Hearing Aid Inspection Checklist (3B-2).
   a. Do not open battery door until after electroacoustic exam is complete.
2. Otoscopy (status of EAC and pinna should be recorded on checklist).
3. Physical examination of “as worn” hearing aids:
   a. Visual inspection of domes and tubing to check for breaks or cerumen occlusion.
   b. Record on the checklist.

D. AS-WORN SIMULATED SPEECHMAP (RIGHT, THEN LEFT) (3B-2)

1. Couple hearing aid to ITE coupler using new sticky tack.
2. On the Verifit, “Test Box Measures” → “Speechmap” (“carrot passage”)
3. Enter participant’s RECD and audiogram.
4. Run simulated speechmap at 55, 65 and 75 dB SPL.
5. Run MPO test.
6. Record SIIs for 65, 55, and 75 dB SPL stimuli on Hearing Aid Inspection Checklist (3B-2).
7. Change format settings to “Table”.
8. Insert USB into Verifit.
9. Select “Print”, enter file name, and press “Continue” to print to USB.
10. Repeat with left hearing aid.

11. Save as an XML file:
    a. Press “Session” on Verifit then select “Store session file”.
    b. Select the “Client ID” box and enter file name (refer to file naming nomenclature sheet in booth) and press “Continue”. This will save file in “Sessions” folder on USB stick along with a style sheet (audioscan.xsl).
12. Listening check
    a. Record the program hearing aids are in on checklist.
    b. Record any abnormalities in hearing aid sound quality on checklist.
    c. Record any hearing device deficiencies on checklist.
13. Open battery door and record battery condition on checklist (record if battery was changed).

E. HEARING AID MAINTENANCE (3B-2)

1. Complete remaining portions of Hearing Aid Inspection Checklist (3B-2) and record any maintenance performed.
2. Run simulated speechmap and MPO in the test box following maintenance for both aids (same as was done on as-worn hearing aids).
    a. Always re-run hearing aids in test box even if no maintenance was needed.
3. Record SIIs for 65, 55, and 75 dB SPL stimuli on Hearing Aid Inspection Checklist.
4. Select “Print”, enter file name, and press “Continue” to print to USB.
5. Save as an XML file:
    a. Press “Session” on Verifit then select “Store session file”.

b. Select the “Client ID” box, enter file name, and press “Continue” (refer to naming nomenclature sheet in booth). This will save the file in “Sessions” folder on USB stick along with a style sheet (audioscan.xsl).

6. Print the post-maintenance simulated speechmap for both ears and put in participant’s folder along with Hearing Aid Inspection Checklist and participant’s As-Worn Aided CST Form (3B-1).
7. Leave sticky tack on coupler to be used for final coupler measurements.

F. AIDED PHAP (T-3), AIDED HHIE (T-4) AND HASS (T-5)

1. When entering participant number, put a “B” on the end to represent Session 3B.
2. Audiologist will have participant follow instructions and complete the surveys on the tablet. Participant will page Audiologist with one-button pager when finished with each survey and Audiologist will start the next survey until all three surveys are complete.

G. POST-MAINTENANCE AIDED CST FORM (3B-3)

1. Seat participant in designated chair (make sure chair is located in correct position on floor).
2. Audiologist will put in participant’s hearing aids.
3. Audiometer settings:
    a. Track 1: Calibrate (already completed during first CST)
    b. Channel 1: 50 dB HL, External A, Left (front speaker), Speaker
    c. Channel 2: 46 dB HL, External B, Right (back speaker), speaker
4. Instructions: “You will hear a woman talking from in front of you. There will be noise behind you. Ignore the background noise, and try to repeat back what the woman says. She will say one sentence at a time. Each group of sentences has a topic. The first topic

is “ear”. Do you have any questions?”

5. Present CST (track 51) at 50 dB HL from loudspeaker located at 0˚ azimuth and elevation with the multi-talker babble from the CST presented at 46 dB HL (+3 dB signal-to-babble ratio) from an identical loudspeaker located at 180˚ azimuth and 0˚ elevation.
6. Have participant complete two passage pairs (“ear” and “liver”; 50 total key words). Provide participant with second passage topic prior to starting second passage.
7. Present sentences one at a time, pausing the CD after each sentence to score.
8. Key words are capitalized and bolded on the scoring sheet. To score, cross out key words that are repeated incorrectly or are not repeated at all.
9. Participant must repeat the word exactly as it is to get it correct. Any other variation of the word, incorrect word, or absence of the word should be marked incorrect.
10. Score = total number of correct words

H. PHAST-R (T-6)

1. Escort participant to the tester side of the booth.
2. When entering participant number, put a “B” on the end to represent Session 3B.
3. Instructions: Place the following items in front of the participant: A telephone, different size batteries (10, 312, 13, 675), magnetic tool for battery removal, cleaning tools: brush, cloth, wax loop, plastic “wire” tube cleaner.
4. Use PHAST-R score sheet on tablet (T-6) to rate participant’s performance.

I. MUSIC/TRAINING QUESTIONNAIRE (3B-4)

1. Administer Music/Training Questionnaire.

J. HEARING AID DECISION FORM #2 (3B-5)

1. Audiologist will now ask: “Now that you’ve worn these hearing aids for 4 more weeks, are you interested in keeping them?”
    a. If the participant responds “no” ask “why?” and record response on Hearing Aid Decision Form #2.
    b. If the participant responds “yes,” ask about complaints or problems with hearing aids and record response on Hearing Aid Decision Form #2.

K. DATA LOGGING FORM (3B-6)

1. Regardless of participant response in “J”, connect hearing aids to software and view data logging.
    a. Record data on Data Logging Form (3B-6).
    b. Print data logging screen.
    c. Reset data logging.
2. If participant elects to keep hearing aids and wants them adjusted, see “L”.
    a. A program swap is NOT considered an adjustment.
3. If participant elects to keep hearing aids and doesn’t want them re-programmed:
    a. Run DFS on both ears.
    b. Complete simulated speechmap test box measures (65, 55, 75, MPO) for both aids.
    c. If programs have been swapped, simulated speechmap should be run with hearing aids in former program 1.
    d. Record SIIs for 65, 55, and 75 dB SPL stimuli on Session 3B Checklist (3B-7).
    e. Select “Print” and enter file name and press “Continue” to print to USB for each ear.
    f. Discard sticky tack.

g. Save as an XML file.

(1) Press “Session” on Verifit, then select “Store session file”.

(2) Select the “Client ID” box and enter file name (refer to file naming nomenclature sheet in booth).

h. Continue to “M”

4. If participant still does not want to keep hearing aids, take participant to CTC to get refund and IU Hearing Clinic card.

a. CTC will complete returned hearing aids process.

L. ADJUSTMENT OF HEARING AIDS (3B-7)

1. If DFS must be run in addition to programming changes, it should be completed in the following order:

a. Programming changes

b. Run and save post-adjustment simulated speechmaps in testbox.

c. Run DFS.

d. Run and save post-DFS simulated speechmaps.

e. If this order cannot be maintained, note this is screenshot and XML file names so that data will not be used.

2. Complete any fine-tuning that the participant requests.

3. Run post-adjustment simulated speechmaps for both hearing aids following fine tuning.

4. Record SIIs for 65, 55, 75 dB SPL stimuli on Session 3B Checklist (3B-7).

a. Change format settings to “Table”.

b. Select “Print” and enter file name and press “Continue” to print to USB.

c. Save as an XML file:

(1) Press “Session” on Verifit then select “Store session file”.

(2) Select the “Client ID” box, enter file name and press “Continue”. This will save file in “Sessions” folder on USB stick along with a style sheet (audioscan.xsl).

5. Run DFS calibration in manufacturer’s software.
6. Run post-DFS simulated speechmaps for both hearing aids, print screen shots and save XML file as described in “b.” and “c.” above.
7. Discard sticky tack.
8. Transfer all Verifit files from USB onto server in appropriate folders.
9. Transfer all files on USB into “USB dump file” on server.

M. HEARING AID ADJUSTMENT LOG (3B-8)

1. Record any adjustments made to the hearing aids.

N. HEARING AID QUESTIONS FORM (3B-9)

1. Record all questions the participant asked during the course of session 3B.

O. HEARING AID STATUS FORM #2 (3B-10)

1. Record whether participant kept aids as-is, requested adjustments or returned aids on Hearing Aid Status Form #2 (3B-10).

P. SCHEDULE FOLLOW-UP

1. Audiologist escorts participant to CTC’s office.
2. CTC schedules a 1-year follow-up appointment and gives participant card to call for questions or adjustment appointments as needed.
3. CTC gives the participant a dri-aid kit and provides instructions on its use.
4. CTC escorts the participant out of the building.
5. CTC fills out Exit Form #2 (3-11).

6. CTC will transfer tablet questionnaire results to appropriate folder on server. CTC will also print the output files from the tablet and place into participant's Session 3B folder.
7. Once results are printed and files have been copied to the server, she will delete the files from the USB and the tablet.
