# Supplementary material for: The Effects of Service-Delivery Model and Purchase Price on Hearing-Aid Outcomes in Older Adults: A Randomized Double-Blind Placebo-Controlled Clinical Trial
Source: Am J Audiol. 2017 Mar;26(1):53–79. doi: 10.1044/2017_AJA-16-0111 (PMC5597084; doi:10.1044/2017_AJA-16-0111)
Supplement: Supplemental Material S2. [file AJA-26-53-s002.pdf]

Supplemental Material S2. Data-collection forms.

Appendix

Session 1

1-1 Phone Script

1-2 Screening Consent Form

1-3 HIPAA Form

1-4 Permission to Contact Form

1-5 Demographic Form

1-6 Audiogram

1-7 Instructions for Loudness Test

1-8 Categories of Loudness

1-9 Unaided CST Form

1-10 Exit Form #1

1-11 MMSE

1-12 Case History

1-13 Eligibility Part 1

1-14 Session 1 Checklist

1-15a Study Consent Form (Typical Purchase Price)

1-15b Study Consent Form (Reduced Purchase Price)

1-16 Medical Waiver

1-17 Eligibility Part 2

Session 2

2-1 Session 2 Audiologist Checklist

2-2 Hearing Aid Decision Form for Audiologist

2-3 Orientation Checklist

2-4 User Guide

2-5 Hearing Aid Case Checklist

2-6 CD Rx Form

2-7 Black binder

2-8 Red binder

2-9 Blue binder

2-10 Hearing Aid Decision Form for CTC

Session 3

3-1 As-Worn Aided CST Form

3-2 Hearing Aid Inspection Checklist

3-3 Post-Maintenance Aided CST Form

3-4 Music/Training Questionnaire

3-5 Final Hearing Aid Decision Form #1

3-6 Data Logging Form

3-7 Session 3 Checklist

3-8 Hearing Aid Adjustment Log

3-9 Hearing Aid Questions Form

3-10 Hearing Aid Status #1

3-11 Exit Form #2

Session 3B

3B-1 As-Worn Aided CST Form

3B-2 Hearing Aid Inspection Checklist

3B-3 Post-Maintenance Aided CST Form

3B-4 Music/Training Questionnaire

3B-5 Hearing Aid Decision Form #2

3B-6 Data Logging Form

3B-7 Session 3B Checklist

3B-8 Hearing Aid Adjustment Log

3B-9 Hearing Aid Questions Form

3B-10 Hearing Aid Status Form #2

Tablet Forms

T-1 Unaided PHAP

T-2 Unaided HHIE

T-3 Aided PHAP

T-4 Aided HHIE

T-5 HASS

T-6 PHAST-R MOD

Miscellaneous

M-1 Unscheduled Visit Checklist

M-2 New Hearing Aid Check-in Protocol

Phone Script

Name:________________________________ Age:______ Date:___________

How did you find out about our study?_______________________________

Mailing Address:____

Phone: _______________________ Email: ____________________________

Hello. This is (name) from the Audiology Research Lab in the Department of Speech and Hearing Sciences at Indiana University Bloomington.

(If follow-up from voicemail: *You had previously responded to an ad about this study.*)

Do you have a few minutes to hear about a new study in our lab?

- **If yes**, go on to next section.
- **If no**, *"is there a better time to reach you or do you have an email address we could send the information to?"*- take down information and either call back or email.

We are currently enrolling participants for a study to compare the benefits resulting from two different ways of providing hearing aids to adults with hearing loss.

Does that sound like something that might interest you?

- **If yes**, go on to next section.
- **If no**, *"thank you for your time, have a nice day."*

In order to participate in this study, you must:

Be a 55-79 year old native English speaker, the ability to read 18 point font,with no history of ear disease or surgery, and have a mild to moderate, stable, sensorineural hearing loss in both ears. A free hearing test will be given to verify the degree and type of your hearing loss. Additionally, you must be a new hearing aid user which means that you have never worn hearing aids or have only worn them for a short period of time (for less than 6 weeks, more than 1 year ago).

Have never been diagnosed with Mutiple Sclerosis or Meniere's Disease.

Have not taken Platinum drugs for cancer (Cisplatin, Carboplatin, Oxaliplatin), or life-saving mycin antibiotics (Gentamicin, Tobramycin, Vancomycin, Kanamycin, Neomycin, or IV-Erythromycin) within the past six months.

Finally, you must have no history of dementia or Alzheimer's disease and a brief cognitive screen will be given to verify this if you decide to participate.

Would you like to hear more about the study?

- **If yes**, go on to next section.
- **If no**, *"thank you for your time, have a nice day."*

Each participant will attend three sessions. The first session is a screening session to see if you are eligible for the remaining two sessions. If you are eligible and agree to participate at that time, then hearing aids will be selected in the second session and you will return to have your performance with the hearing aids evaluated during the third and final session.

The first and second sessions last between 1 and 2 hours and take place on two consecutive days. The third session lasts approximately 1 hour and will be scheduled approximately 6 weeks after the first two sessions.

The study involves three groups of participants who will all purchase the same high-quality hearing aids. Consistent with standard clinical practice, participants will have a 6-week trial period after which the hearing aids may be returned for a full refund, less any credits or payments received by you previously for your participation. The groups include one that will be fit with hearing aids with more assistance from an audiologist and a second group that will independently choose their own hearing aids following instructions provided to them. The third group will receive "placebo" hearing aids that do not provide any amplification. However, at the end of the study, participants in this group will be offered the opportunity to have their hearing aids programmed to provide appropriate amplification and can retry the hearing aids for another six weeks without penalty. Participants will be randomly placed into one of these three groups. If you are eligible to participate in Sessions 2 and 3 and decide to do so, you will be paid an amount for completion of those last two sessions that is equal to a 16.7% discount on high-quality hearing aids as compensation for your time.

"The pair of hearing aids are state-of-the-art high-end devices made by a major manufacturer and, if you qualify for the study AND decide, at the end for the first session, to purchase hearing aids, they can be purchased at a price of $3600 for the pair. This includes a 3-year warranty and all hearing aids are sold with a 6-week money-back guarantee if you are unsatisfied with the hearing aids for any reason. If you return to complete the measurements in Sessions 2 and Session 3, you will receive a credit or payment of $300 for each session. This effectively reduces the price of the hearing aids from $3600 to $3000 for the pair."

Does this still sound like something you might be interested in?

- **If yes**, go on to next section.
- **If no**, "thank you for your time, have a nice day."

During the sessions you will be completing audiologic testing, which involves responding to tones and speech, completing written questionnaires, and, if eligible and willing to participate, purchasing two hearing aids.

Would you like to schedule a screening to determine if you are eligible to participate? *(Agreeing to participate in the screening does not commit you to participating in the full 3-session study, but only to completing the first screening session. If you are eligible to participate, we will ask you again, at that time, whether you are interested in purchasing hearing aids and in completing the remaining two sessions.)*

- **If yes**, schedule all three sessions, with understanding that sessions 2 and 3 will be cancelled if subject does not meet eligibility requirements.
- **If no**, "thank you for your time, have a nice day."

Screening Session 1 Date: __________________________ Time: __________

Session 2 Date: __________________________ Time: __________

Session 3 Date: __________________________ Time: __________

Contact log ____
Scheduled on calendar ____
Audiologist Assigned ____
Letter sent ____
Confirmation call 1 ____ reminder to bring a list of medications with you ____
Confirmation call 2 ____
Confirmation call 3 ____

INDIANA UNIVERSITY INFORMED CONSENT STATEMENT

Screening for "Evaluating Hearing Aid Service-Delivery Models"

We are conducting a research study examining different ways of providing hearing aids to adults with hearing loss. You responded to an initial advertisement for this study. To determine your eligibility for this study, you need to complete some initial tests and provide us with some information. This is referred to as the "screening" of potential study participants. We ask that you read this form and ask any questions you may have before agreeing to be screened for this study. If you are found to be eligible for the larger study after this screening, you will be presented with a separate consent form for that study. Agreeing to the screening does not commit you to participation in the larger study.

The study, and the screening for it, is being conducted by Larry E. Humes, Distinguished Professor, Department of Speech and Hearing Sciences, Indiana University. It is funded by the National Institutes of Health (NIH).

STUDY PURPOSE

The purpose of this screening is to determine your eligibility for the larger study which compares the benefits resulting from two different ways of providing hearing aids to adults with hearing loss.

NUMBER OF PEOPLE TAKING PART IN THE STUDY:

We are estimating that as many as 400 individuals responding to the recruitment ads may need to be screened. We are targeting 210 subjects for the larger research study.

ELIGIBILITY REQUIREMENTS:

In order to participate in this screening, subjects must:

- be age 55-79 years
- native English speakers
- have no prior experience with hearing aids
- **the ability to read 18 point font**

PROCEDURES FOR THE STUDY:

If you agree to participate in the screening, you will be completing several tasks. This will include a detailed case history, which confirms the above eligibility requirements and will also ask questions regarding history of ear disease or surgery, medications, etc. In addition, you will be given a free hearing test to verify the degree and type of hearing loss. The degree and type of hearing loss are important for participation in the larger study as you must be a suitable candidate for the hearing aids to be used in that study. Finally, you will complete the Mini Mental State Examination, a brief cognitive screen to verify the absence of dementia or Alzheimer's disease.

The entire screening should take approximately 60-75 minutes to complete. If you are found to be eligible for the larger study, you will be given the opportunity to participate in that study and must sign an additional consent form to do so.

RISKS OF TAKING PART IN THE STUDY:

Protocol 1111007504 IRB Approved

While in the study, the risks are minimal. The procedures are similar to those employed routinely at clinical audiological facilities. There is the potential that some of the stimuli in certain conditions may be perceived as being "loud." However, the presentation of such stimuli will not be at sound levels potentially damaging to your auditory system, even if perceived to be "loud."

BENEFITS OF TAKING PART IN THE STUDY:

By responding to the recruitment ad for this study, a comprehensive hearing evaluation by a clinically certified audiologist has been provided at no charge.

Regarding the larger study, outcomes of this investigation may aid hearing professionals in making decisions about how best to deliver hearing aids to adults with hearing loss.

ALTERNATIVES TO TAKING PART IN THE STUDY:

An alternative to participating in this screening is to choose not to participate. If you do not participate, but are still interested in having your hearing tested, you are welcome to do so at the IU Hearing Clinic, following normal clinical procedures and at normal clinical fees, or elsewhere at your discretion.

CONFIDENTIALITY

Efforts will be made to keep your personal information confidential. Your screening results may be used in the analysis and publication of study results, but only in aggregate (group) form. We cannot guarantee absolute confidentiality. Your personal information may be disclosed if required by law. Your identity will be held in confidence in reports in which the study may be published and databases in which results may be stored.

Organizations that may inspect and/or copy your research records for quality assurance and data analysis include groups, such as the study investigator and his/her research associates, the IU Institutional Review Board or its designees, the study sponsor, the National Institutes of Health (NIH), and (as allowed by law) state or federal agencies, specifically the Office for Human Research Protections (OHRP) and the Food and Drug Administration (FDA), who may need to access your medical and/or research records.

PAYMENT

You will receive no payment for taking part in this screening.

CONTACTS FOR QUESTIONS OR PROBLEMS

For questions about this screening or a research-related injury, contact the researcher, Larry Humes at [redacted] or by email at [redacted].

For questions about your rights as a research participant or to discuss problems, complaints or concerns about a research study, or to obtain information, or offer input, contact the IU Human Subjects Office at [redacted] or [redacted].

VOLUNTARY NATURE OF STUDY

Protocol 1111007504 IRB Approved

Taking part in this screening is voluntary. You may choose not to take part or may discontinue the screening at any time. Discontinuing the screening will not result in any penalty or loss of benefits to which you are entitled. Your decision whether or not to participate in this screening will not affect your current or future relations with the investigator(s) or with the IU Hearing Clinic.

SUBJECT'S CONSENT

In consideration of all of the above, I give my consent to participate in this screening for the larger research study.

I will be given a copy of this informed consent document to keep for my records. I agree to take part in this screening.

Subject's Printed ______________________________ **Name:**

Subject's Signature:______________________________ ____________________
Date:
(must be dated by the subject)

Printed Name of Person Obtaining Consent:______________________________

Signature of Person Obtaining Consent: ______________________________**Date**:____________________

Consent form date: 10/15/12

Protocol 1111007504 IRB Approved

INDIANA UNIVERSITY
AUTHORIZATION FOR THE RELEASE OF HEALTH INFORMATION FOR RESEARCH
HEALTHY SUBJECT RESEARCH PARTICIPANTS

Introduction: You have the right to decide who may review or use your Protected Health Information ("PHI"). The type of information that may be used is described below. When you consider taking part in a research study, you must give permission for your PHI to be used and disclosed by the Research Team for the specific purpose of this research study.

<u>What does this authorization relate to?</u> This authorization relates to the following study:

**Larry E. Humes**	**1111007504**
PRINCIPAL INVESTIGATOR (in charge of Research Team)	***IRB PROTOCOL #***
	SPONSOR # **R01-DC011771-01**
NAME OF RESEARCH PARTICIPANT	***BIRTHDATE***
STREET ADDRESS	***CITY, STATE & ZIP CODE***

<u>What information will be used for research purposes?</u> The PHI used for this research study will include information that you provide to the Research Team and any data and reports created by the Research Team that may include this information. Your medical records will not be requested or otherwise accessed.

<u>Who can access your PHI for the study?</u> The principal investigator and research team may share my PHI (or the PHI of the individual(s) whom I have the authority to represent), with the following persons or groups for the research study: IU Institutional Review Board and its designees, Research Sponsor and its representatives, Research Organizations, the Department of Health & Human Services or other US or foreign government agencies as required by law, and to the Food and Drug Administration (FDA) or a person subject to the jurisdiction of the FDA in order to audit or monitor the quality, safety or effectiveness of the product or activity.

The **Research Team** includes the Principal Investigator, his/her staff, research coordinators, research technicians and other staff members who provide assistance to the Research Team. If there is a **Research Sponsor(s),** this shall include: National Institutes of Health and any **Research Organizations** who provided assistance to the **Research Sponsor(s)** including, but not limited to: ____.

Expiration date of this Authorization: This authorization is valid until the following date or event:

- ☐ Specify Date ___/___/____
- ☒ End of the Study
- ☐ Other: _______________________
- ☐ None; authorization is valid indefinitely

Efforts will be made to ensure that your PHI will not be shared with other people outside of the research study. However, your PHI may be disclosed to others as required by law and/or to individuals or organizations that oversee the conduct of research studies, and these individuals or organizations may not be held to the same legal privacy standards as are doctors and hospitals. Thus, the Research Team cannot guarantee absolute confidentiality and privacy.

INDIANA UNIVERSITY
AUTHORIZATION FOR THE RELEASE OF HEALTH INFORMATION FOR RESEARCH
HEALTHY SUBJECT RESEARCH PARTICIPANTS

I have the right:

1. To refuse to sign this form. Not signing the form will not affect my regular health care including treatment, payment, or enrollment in a health plan or eligibility for health care benefits. However, not signing the form will prevent me from participating in the research study above.

2. To review and obtain a copy of my personal health information collected during the study. However, it may be important to the success and integrity of the study that persons who participate in the study not be given access until the study is complete. The Principal Investigator has discretion to refuse to grant access to this information if it will affect the integrity of the study data during the course of the study. Therefore, my request for information may be delayed until the study is complete.

3. To cancel this release of information/authorization at any time. If I choose to cancel this release of information/authorization, I must notify the Principal Investigator for this study **in writing** at: *Department of Speech and Hearing Sciences*, *Attn: Dr. Larry Humes*, [redacted]. However, even if I cancel this release of information/authorization, the Research Team, Research Sponsor(s) and/or the Research Organizations may still use information about me that was collected as part of the research project between the date I signed the current form and the date I cancel the authorization. This is to protect the quality of the research results. I understand that canceling this authorization may end my participation in this study.

4. To receive a copy of this form.

I have had the opportunity to review and ask questions regarding this release of information/authorization form. By signing this release of information/authorization, I am confirming that it reflects my wishes.

____
Printed name of Individual/Legal Representative

____
Signature of Individual/Legal Representative *Date*

**If signed by a legal representative; state the relationship and identify below the authority to act on behalf of the individual's behalf.*

***Individual is:** ☐ a Minor ☐ Incompetent ☐ Disabled ☐ Deceased

***Legal Authority:**

☐ Custodial Parent ☐ Legal Guardian ☐ Executor of Estate of the Deceased
☐ Power of Attorney Healthcare ☐ Authorized Legal Representative
☐ Other: ___________

For IU Human Subjects Office Use ONLY

IRB REVIEWED

Study #1211010066

PERMISSION TO CONTACT FOR OTHER RESEARCH PROJECTS

The Audiology Research Laboratory (ARL) is part of the Department of Speech and Hearing Sciences at Indiana University and often conducts research in conjunction with the I.U. Hearing Clinic. Researchers in the lab are continually trying to uncover new information about human hearing, better understanding of problems caused by hearing loss and new ways to help people with hearing problems. Research participants are scheduled for testing at their convenience and are provided with free parking. In addition, they often receive other benefits. For example, in several recent hearing aid research projects, participants have received discounts from 40 – 100% on the latest hearing aid technology.

If research studies arise in the future in the ARL, would you be willing to be contacted by a researcher to see if you are interested in participating in the project? If so, please check "Yes" below. If you check "Yes" below, you are just indicating a willingness to be contacted in the future and are in no way obligated to participate in a project until it has been fully explained to you and you agree to participate. If you change your mind and decide that you do not wish to be contacted, you may contact the director of the ARL, Larry Humes, to have your name removed from the list. A copy of this form is provided for your records. If you have any questions about this information, please contact Larry Humes ([redacted]).

_____ Yes, I am willing to be contacted in the future as a possible subject for research in this laboratory.

_____ No, I am not interested in being contacted in the future.

__________________________ __________
Participant's Signature Date

__________________________ __________
Examiner's Signature Date

____
Phone number/e-mail address

Demographic Form
(Screening)

1) <u>Gender</u> (check one)

___ Male

___ Female

2) <u>Ethnic Category</u> (check one)

___ Hispanic or Latino

___ Not Hispanic or Latino

3) <u>Racial Categories</u> (check all that apply)

___ American Indian/Alaska Native

___ Asian

___ Native Hawaiian or Other Pacific Islander

___ Black or African American

___ White

******* For Lab Use Only *******

4) Date: ____________

5) Grant Type: ABCD

6) Date of Data Entry Completion:____________

[redacted]

AUDIOLOGIC RECORD

Indiana University Clinical Trial Group
Bloomington, IN 47405 [redacted]

[redacted]

Subject Number:______________________ Date:__________ Age:________ DOB:______________

Tested by:______________________ Referral Source:______________________

Audiometer: GSI-61 Earphone: 3A TDH

Research Project: ABCD Test Reliability: good fair poor

Session: 1 2 3 4 P/T follow-up Procedure: VRA CPA Standard

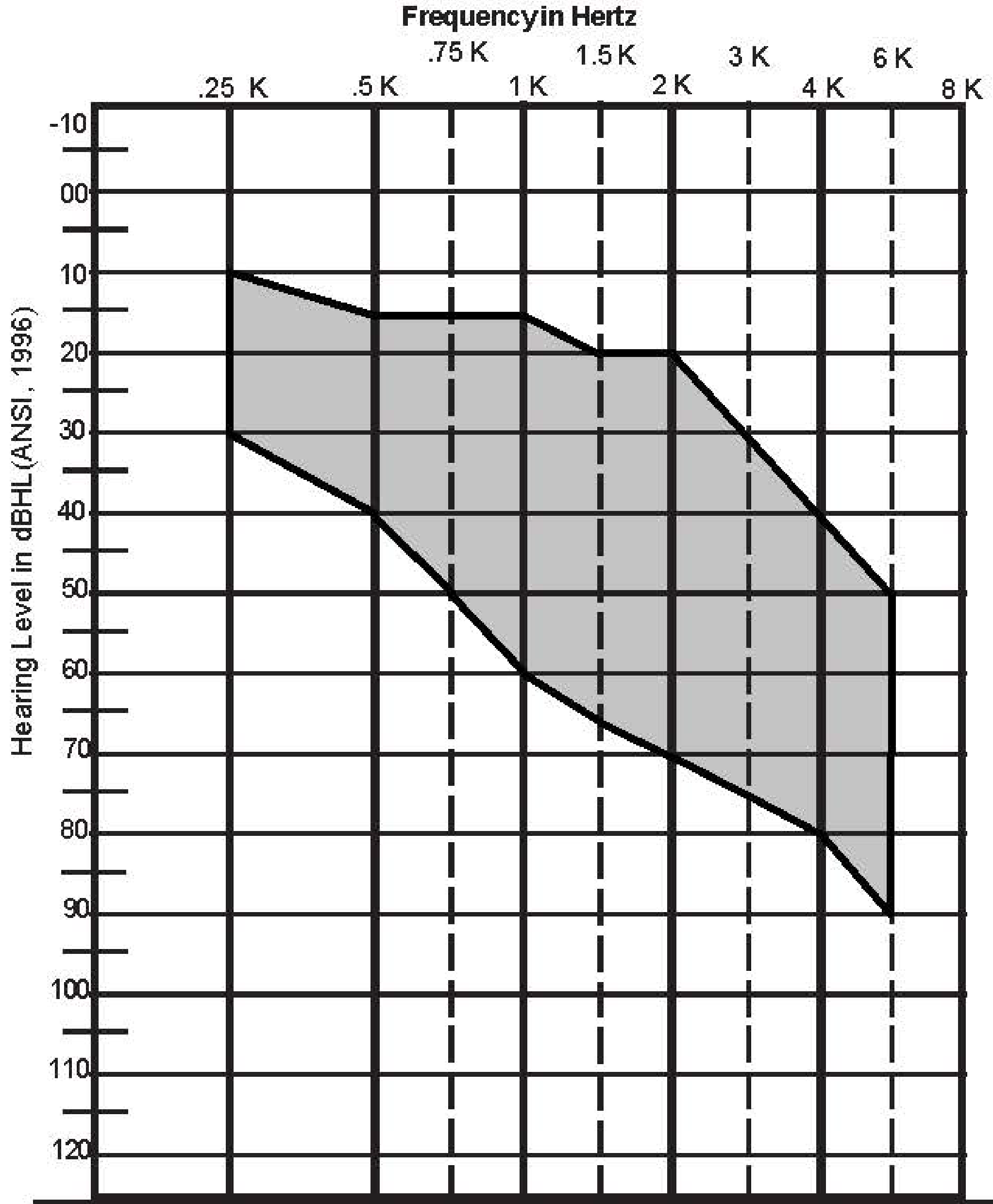

	AC Unmasked	AC Masked	BC Unmasked	BC Masked	Sound Field	Aided Sound Field	Ex. of no response
R	O	Δ	<	[	Ø		
L	X	□	>	]	X		
U			^		S	A	

Speech Audiometry

SRT Material:______________ MLV

WRS Material:______________ CD

CST Material:______________ CD

	R	L	Bone	Sound Field	Sound Field Aided
PTA__,__,& __ kHz					
SRT or SAT					
EM for SRT or SAT					
WRS % Quiet / dB HL	/	/	/	/	/
CST # Correct / dB HL	/	/	/	/ 50	/ 50
S/N Ratio				+3	+3
EM for WRS					

EM in dB

											R
											L
											R
											L

Loudness Discomfort Level (LDL)

		.5 K	1 K	2 K	3 K	4 K
R	SPL					
	HL					
L	SPL					
	HL					

Acoustic-Reflex Testing

	Stim in	Meas in	.5 K	1 K	2 K	4 K	Reflex Decay .5 K	Reflex Decay 1 K
Contra (HL)	R	L						
	L	R						
Ipsi (HL)	R	R						
	L	L						

Comments

____
____
____
____
____
____

Tympanometry

	R	L
Pass/Fail		
Equivalent Volume child norm .4-1.0 adult norm .6-1.5	ml	ml
Static Compliance child norm .2-.9 adult norm .3-1.4	cc	cc
Peak Compliance child norm -150-+60 adult norm -110-+50	daPa	daPa

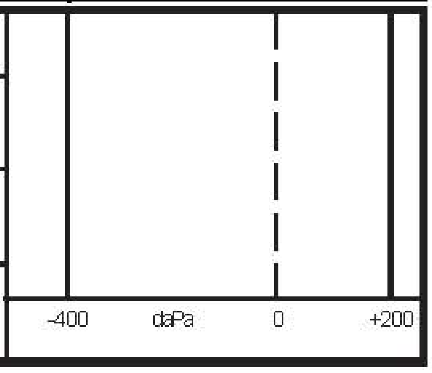

Instructions for Loudness Test:

The purpose of this test is to find your judgments of the loudness of different sounds.

You will hear sounds that increase and decrease in volume. You must make a judgment about how loud the sounds are. Pretend you are listening to the radio at that volume. How loud would it be?

After each sound, tell me which of these categories best describes the loudness.

Keep in mind that an uncomfortably loud sound is louder than you would ever choose on your radio no matter what mood you are in.

Categories of Loudness

7. Uncomfortably loud
6. Loud, but okay
5. Comfortable, but slightly loud
4. Comfortable
3. Comfortable, but slightly soft
2. Soft
1. Very soft

SUBJECT #: ____________ TEST DATE: ________

EXAMINER'S INITIALS: ________

Unaided CST Form

TRACK 32

(CST 50 dBHL; +3 dB SNR; SP: 0 degrees N: 180 degrees; Binaural; Unaided)

Text for the CONNECTED SPEECH TEST

*(scoring words are capitalized, mark through each incorrect response in ink with an **X**, and record the total number of words correct below)*

Passage: 1- WINDOW

Windows **PROVIDE LIGHT** and air to **ROOMS.**
Windows were **ONCE COVERED** with **CRUDE SHUTTERS.**
Later, oiled **PAPER** was **USED** for windowpanes.
GLASS windows **FIRST** appeared in ancient Rome.
COLORED glass was used in European **WINDOWS**.
SOME CHURCHES were **FAMOUS** for their **BEAUTIFUL** windows.
These windows **DISPLAYED PICTURES** from the **BIBLE**.
PIECES of glass were **HELD** together by lead.
SUCH windows **MAY** be seen in French cathedrals.
English churches also contain **STAINED** glass windows.

Passage 2- GLOVE

Gloves are **CLOTHING WORN ON** the **HANDS**.
The **WORD "GLOVE" MEANS** "palm of the hand."
CRUDE GLOVES were **WORN** by **PRIMITIVE MAN**.
Greeks wore **WORKING** gloves to **PROTECT** their hands.
The **ROMANS USED** gloves as a sign of **RANK**.
Knights used to fasten gloves to their helmets.
The gloves **SHOWED** their **DEVOTION** to their **LADIES**.
A glove thrown on the **GROUND SIGNALED** a challenge.
Knights threw them at their enemy's feet.
FIGHTING STARTED WHEN the enemy picked up the glove.

*** Total Number of CORRECTLY identified words: ____/50**

SUBJECT #: ____________ TEST DATE: ________

EXAMINER'S INITIALS: ________

Exit Form #1

Reason for Subject Exit:

Does not meet eligibility criteria:

❒ Does not fall within age limit of study

❒ Is not a native English speaker

❒ Has used hearing aids within past year

❒ Has used hearing aids for 6 or more weeks

❒ Hearing loss does not fall within range specified for study

❒ MMSE score was 25 or lower

❒ Currently has indication of medically or surgically treatable ear-related condition

❒ Has bilateral flat, type B, tympanograms

❒ Has known fluctuating or rapidly progressive hearing loss

❒ Has a cognitive, medical, or language-based condition which may limit his/her ability to complete all test procedures

❒ Is currently taking or has recently taken a Platinum drug for cancer or live-saving Mycin antibiotics

❒ Has Multiple Sclerosis

❒ Has Meniere's Disease

❒ Is not interested in purchasing hearing aids from IU Clinical Trial Group

❒ Is 'private-pay patient' with 3rd party reimbursement for hearing aid costs

❒ Will not waive medical clearance

❒ Will not sign consent form 1

❒ Will not sign consent form 2

Miscellaneous:

❒ Inability/Unwillingness to participate

❒ Did not return for session 2

____

Investigator's Signature Date Signed

Image of MMSE-2 not shown here due to potential copyright issues. Please see Folstein, M. F., Folstein, S. E., White, T., & Messer, M. A. (2010) Mini-Mental State Examination, 2nd edition.

SUBJECT #: ____________ TEST DATE: ________

EXAMINER'S INITIALS: ________

Case History

AGE ____ years Date of Birth _______________ Gender: M or F
(please circle)

I. Etiology (Cause) of hearing loss (please choose one answer)

❒ Presbycusis (aging) ❒ Noise exposure ❒ Unknown ❒ Not Applicable
(I do not have hearing loss)

II. Duration ❒ Not Applicable

A. Duration of loss _____ year(s)

B. How much time passed from the time when you or others (family, friends, co-workers) first suggested you <u>might</u> have trouble hearing, and the time that you made an appointment with a doctor or an audiologist to have your hearing tested?
_____ year (s)

III. Noise exposure history

Have you worked previously in a noisy job (factory, carpentry, etc.)? __ Yes __ No
If "Yes", how many years? ____

Were you ever in the military? ___ Yes ___No
If "Yes", how many years? ____

Do you have any noisy hobbies (woodworking, snowmobiles, band, etc.)? __Yes __No
If "Yes", how many years? ____

IV. Educational level

Highest Level of Education Completed: **(please choose one answer)**

- ❒ Less than high school
- ❒ High school
- ❒ Some college
- ❒ Vocational or technical degree
- ❒ College degree
- ❒ Some graduate school
- ❒ Master's degree
- ❒ Doctorate or medical degree

V. Occupation

Previous or current occupation: **(please choose one answer)**

- ❒ Laborer
- ❒ Trade union
- ❒ Clerical
- ❒ Professional
- ❒ Other

How long in that position? ______ years

If retired, how long? ______ years

VI. Socioeconomic status **(please choose one answer)**

Indicate total household annual income for the last tax year (please consider all sources i.e., salary and wages, retirement benefits, investments, rent, etc...)

- ❒ $5,000 – 15,000
- ❒ $15,000-25,000
- ❒ $25,000-35,000
- ❒ $35,000-45,000
- ❒ > $45,000

VII. Hearing aid experience

Are you currently using hearing aid(s), even for a brief period of time?

❒ Yes ❒ No

Have you ever used hearing aid(s)? ❒ Yes ❒ No

If you answered "yes" to either of the questions above, please provide the following information:

A. Approximate date hearing aids were last worn: ____________________

B. Total amount of time hearing aid(s) have been used: **(please choose one answer)**

- ❒ Less than or equal to 6 weeks
- ❒ 7 weeks-11 months
- ❒ 1-3 years
- ❒ 4-10 years
- ❒ >10 years

C. Monaural or Binaural Use: **(please choose one answer)**

- ❒ Consistently wore just the RIGHT aid
- ❒ Consistently wore just the LEFT aid
- ❒ Wore both aids consistently

D. During the time of use, did you typically wear your hearing aid(s) for: **(please choose one answer)**

- ❒ < 1 hour per day
- ❒ 1- 3 hours per day
- ❒ 4-6 hours per day
- ❒ 7-10 hours per day
- ❒ >10 hours per day

VIII. Medications

Please list any current medications and indicate both your **dosage** and **how long you've been taking each one.**

____
____
____

IX. Living Arrangements **(please choose one answer)**

❒ Live alone independently

❒ Live with spouse or significant other

❒ Live with other family members

❒ Live with spouse and other family members

X. Social Activities (such as visiting with a friend or in a group, entertaining in your home, eating in a restaurant, playing cards, etc.) **(please choose one answer)**

❒ Regularly participate in social activities with family/friends (almost daily)

❒ Occasionally participate in social activities with family/friends (weekly)

❒ Rarely participate in social activities with family/friends (monthly or less)

XI. Outside Interests (such as religious services, movies, theatre, sporting events, lectures, etc.) **(please choose one answer)**

❒ Regularly attend outside activities (once or more a week)

❒ Occasionally attend outside activities as above (monthly)

❒ Rarely attend outside activities (once or twice each year)

XII. Ethnic Category (check one)

❐ Hispanic or Latino ❐ Not Hispanic or Latino

XIII. Racial Category (check all that apply)

❐ American Indian/ Alaska Native

❐ Asian

❐ Native Hawaiian or Other Pacific Islander

❐ Black or African American

❐ White

XIV. Medical Conditions/Specific Medications

Please answer "yes" or "no" to the following questions:

	Yes	No
1. Do you have diabetes?	❐	❐
2. If yes to #1, is your diabetes well-controlled by medication or diet?	❐	❐
3. Do you currently take a diuretic (Lasix, etc.)?	❐	❐
4. Do you currently take 8 or more aspirins per day?	❐	❐

SUBJECT #: ____________ TEST DATE: ________

EXAMINER'S INITIALS: ________

Eligibility Part 1

Inclusion Criteria (all answers must be *yes* for entry into the study)

yes❒ no❒ 1. Subject is greater than 55 years old and less than 80 years old.
yes❒ no❒ 2. Subject is native English speaker.
yes❒ no❒ 3. Subject indicated he/she has never used hearing aids previously.
yes❒ no❒ 4. Subject's air conduction thresholds are bilaterally symmetrical (within 20 dB from 250-2000Hz) and fall between the following limits:

250 Hz:	10-30 dB HL	2000 Hz:	20-70 dB HL
500 Hz:	15-40	3000Hz:	30-75
1000 Hz:	15-60	4000 Hz:	40-80
1500Hz:	20-65	6000 Hz:	50-90

yes❒ no❒ 5. Subject has passed the Mini-Mental State Exam (score > 25).

Exclusion Criteria (all answers must be *no* for entry into the study)

yes❒ no❒ 1. Subject **currently** has any indication of a medically or surgically treatable ear-related condition.
yes❒ no❒ 2. Subject has bilateral flat, type B, tympanograms.
yes❒ no❒ 3. Subject has known fluctuating or rapidly progressive hearing loss.
yes❒ no❒ 4. Subject has any cognitive, medical, or language-based condition which may limit his/her ability to complete all test procedures.
yes❒ no❒ 5. Subject is currently taking or has recently taken any of the following medication(s):
_____ Platinum drug for cancer (Cisplatin, Carboplatin, Oxaliplatin)
_____ Life-saving Mycin antibiotics (Gentamicin, Tobramycin, Vancomycin, Kanamycin, Neomycin, or IV- Erythromycin)
yes❒ no❒ 6. Subject has **multiple sclerosis**.
yes❒ no❒ 7. Subject has **Meniere's Disease**.

____

Investigator's Signature Date Signed

Session 1 Audiologist Checklist

Audiologic Evaluation

___ **Otoscopy**

___ **Tymps & Ipsi Reflexes** (.5, 1, 2 kHz)

___ **Air Conduction** (.25, .5, 1, 1.5, 2, 3, 4, 6, & 8 kHz)

*If ineligible at this point complete rest of audio eval (except LDL & CST), counsel, and escort out of building. If patient is ineligible, family member may come from the waiting room at this time for counseling.

___ **SRT** (MLV)

___ **WRS** (VA CD, W-22)

___ **LDL @ 500 and 3000 Hz**

___ **Bone Conduction** (.25, .5, 1, 1.5, 2, 3, & 4 kHz)

___ **Unaided CST** (Two passages; Track 32)

To be eligible, subject's Θs need to be bilaterally symmetrical mild to moderate SNHL, within fitting range of hearing aid (see shaded region on audiogram).

Asymmetry: > 20 dB at any frequency 250-2000 Hz. Subject may have one Θ at a single frequency in each ear that is outside the shaded area by ≤ 10 dB and still be eligible for study. If subject's Θs are close to, but outside our accepted range, "FLAG" the file and notify PI and CTC.

Forms, Counseling, & RECD

___ **Counseling** (At this time, patient's family member can come from the waiting room)

___ **MMSE** (≥ 26)

___ **Case History Form** (Ensure all items are completed)

___ **Eligibility Part 1**

___ **RECD**

**Incorrect WRS Tally**	
Right	Left

**LDL Measurements**									
**500 Hz**					**3,000 Hz**				
Right					Right				
Left					Left				

INDIANA UNIVERSITY INFORMED CONSENT STATEMENT

Evaluating Hearing Aid Service-Delivery Models

You are invited to participate in a research study examining different ways of providing hearing aids to adults with hearing loss. You were selected as a possible subject because you responded to an initial advertisement for this study and were subsequently found to be eligible for the study. We ask that you read this form and ask any questions you may have before agreeing to be in the study.

The study is being conducted by Larry E. Humes, Distinguished Professor, Department of Speech and Hearing Sciences, Indiana University. It is funded by the National Institutes of Health (NIH).

STUDY PURPOSE

The purpose of this study is to compare the benefits resulting from two different ways of providing hearing aids to adults with hearing loss.

NUMBER OF PEOPLE TAKING PART IN THE STUDY:

If you agree to participate, you will be one of 210 subjects who will be participating in this research.

PROCEDURES FOR THE STUDY:

If you agree to participate in the study, you will receive an auditory test that requires you to listen and respond to words or sentences presented via loudspeakers under different listening conditions. Your responses will be recorded using a written answer sheet. You will also complete two surveys about difficulties you experience in everyday listening situations.

You will then be assigned randomly (by chance) to one of three groups. All three groups receive identical hearing aids. For one group, the Placebo Group, however, the hearing aids are programmed to be minimally functional, only providing enough boost or amplification to make up for any loss of sound resulting from having something in your ears. That is, for the members of the Placebo Group, the boost provided by the hearing aids is insufficient to provide much help for your hearing loss.

For the other two groups, Group AB and Group CD, the hearing aids will provide boost to help compensate for your hearing loss, as is normally the case. For Group AB, the audiologist will be much more involved in selecting and fitting the hearing aids for you. For Group CD, you will be much more involved in selecting the hearing aids for yourself. After the hearing aids have been selected, you will be asked to participate in one follow-up session approximately 6 weeks later. At this session, you will repeat the listening test and surveys completed in the initial session prior to wearing hearing aids. You will also complete a third survey in writing at this time. Just like any person purchasing hearing aids in the U.S., you will then be given the option to return the hearing aids, if desired, and receive a full refund of the purchase price of the hearing aids, less any credits or payments made to you for completion of test sessions.

Those assigned randomly to the Placebo Group will be informed of their assignment at that time and will be given the opportunity to receive another 6-week trial of the hearing aids after they have been adjusted to provide enough boost to compensate for their hearing loss. If members of the Placebo Group elect to pursue another 6-week trial period and are dissatisfied with the hearing aids at the end of that additional 6-week trial period, they will then be given the option to return the hearing aids at that time, if desired, and receive a full refund of the purchase price of the hearing aids, less any credits or payments made to you for completion of test sessions.

Protocol 1111007504 IRB Approved

In total, two sessions are required for this study. These two sessions are designated “Session 2” and “Session 3” in this study. Each session should require no more than two hours for completion.

RISKS OF TAKING PART IN THE STUDY:

While in the study, the risks are minimal. If you have been randomly assigned to the Placebo Group, you may be frustrated at the lack of benefit provided by your hearing aids during the initial 6-week trial period. As noted, members of this group will have the opportunity for another 6-week trial period after the hearing aids have been adjusted appropriately to compensate for their hearing loss. For the Placebo Group, there is also potential risk of the hearing aid decreasing auditory awareness. The presence of the device in the subject’s ear canal may serve as a sound attenuator and make the participant less aware of sound. Steps have been taken to minimize this risk. First, the devices to be fitted are “open-fit” devices which minimize plugging up the ear canal. Second, the placebo devices will be fitted using real-ear measurements to verify that the hearing aids are acoustically transparent, neither amplifying nor attenuating incoming sounds.

For those in the CD and AB groups, it is possible that outcomes obtained will differ, being inferior for one of the groups. Regardless of outcome, the participants in either of these branches will be given the opportunity to receive services identical to those received by subjects in the other branch after the completion of Session 3 and without additional charges.

In addition, because hearing aids and eartips will be inserted into the subjects’ ears, there is some minimal risk for transmission of infection. This risk will be minimized by the thorough cleaning of all hearing aids and eartips tried by the patient after each subject’s use.

BENEFITS OF TAKING PART IN THE STUDY:

Outcomes of this investigation may aid hearing professionals in making decisions about how best to deliver hearing aids to adults with hearing loss.

ALTERNATIVES TO TAKING PART IN THE STUDY:

An alternative to participating in the study is to choose not to participate. If you do not participate, but are still interested in receiving hearing aids, you are welcome to do so at the IU Hearing Clinic, following normal clinical procedures, or elsewhere at your discretion. If requested, we will provide you with a copy of the results of your initial hearing test.

CONFIDENTIALITY

Efforts will be made to keep your personal information confidential. We cannot guarantee absolute confidentiality. Your personal information may be disclosed if required by law. Your identity will be held in confidence in reports in which the study may be published and databases in which results may be stored.

Organizations that may inspect and/or copy your research records for quality assurance and data analysis include groups such as the study investigator and his/her research associates, the IU Institutional Review Board or its designees, the study sponsor, the National Institutes of Health (NIH), and (as allowed by law) state or federal agencies, specifically the Office for Human Research Protections (OHRP) and the Food and Drug Administration (FDA), who may need to access your medical and/or research records.

Protocol 1111007504 IRB Approved

PAYMENT

You will be paid $300 after the completion of Sessions 2 and 3 of this study for a total payment of $600. This amounts to 16.7% of the $3600 purchase price for your hearing aids. The first payment of $300 will be applied as a credit toward the $3600 purchase price with the balance of $3300 payable at the end of Session 2. The second payment, at the end of Session 3, will be issued to you as a check for $300, or a $300 credit will be applied to your credit card account..

CONTACTS FOR QUESTIONS OR PROBLEMS

For questions about the study or a research-related injury, contact the researcher, Larry Humes at [redacted] or by email at [redacted].

For questions about your rights as a research participant or to discuss problems, complaints or concerns about a research study, or to obtain information, or offer input, contact the IU Human Subjects Office at [redacted] or [redacted].

VOLUNTARY NATURE OF STUDY

Taking part in this study is voluntary. You may choose not to take part or may leave the study at any time. Leaving the study will not result in any penalty or loss of benefits to which you are entitled. Your decision whether or not to participate in this study will not affect your current or future relations with the investigator(s).

Your participation may be terminated by the investigator without regard to your consent in the following circumstances: If it is determined that you have significant earwax buildup that interferes with test procedures and you choose to not have it removed.

If you withdraw or are terminated from the study before completion, your partial data may be used for data analysis unless you request that it not be used.

COSTS

Taking part in this study may lead to added costs to you or your insurance company. You or your insurance company will be responsible for the following costs: Having your ears cleared of wax at your physician's office or location of your choosing. You will not be responsible for these study-specific costs: Having your ears cleared of wax by the audiologists working on the study.

SUBJECT'S CONSENT

In consideration of all of the above, I give my consent to participate in this research study.

I will be given a copy of this informed consent document to keep for my records. I agree to take part in this study.

Subject's Printed ______________________________ **Name:**

Subject's Signature:______________________________ ____________________

Date:

Protocol 1111007504 IRB Approved

Printed Name of Person Obtaining Consent:________________________________

Signature of Person Obtaining Consent: ______________________________________ **Date**:____________________

TPP Consent form date: 10/15/12

Protocol 1111007504 IRB Approved

INDIANA UNIVERSITY INFORMED CONSENT STATEMENT

Evaluating Hearing Aid Service-Delivery Models

You are invited to participate in a research study examining different ways of providing hearing aids to adults with hearing loss. You were selected as a possible subject because you responded to an initial advertisement for this study and were subsequently found to be eligible for the study. We ask that you read this form and ask any questions you may have before agreeing to be in the study.

The study is being conducted by Larry E. Humes, Distinguished Professor, Department of Speech and Hearing Sciences, Indiana University. It is funded by the National Institutes of Health (NIH).

STUDY PURPOSE

The purpose of this study is to compare the benefits resulting from two different ways of providing hearing aids to adults with hearing loss.

NUMBER OF PEOPLE TAKING PART IN THE STUDY:

If you agree to participate, you will be one of 210 subjects who will be participating in this research.

PROCEDURES FOR THE STUDY:

If you agree to participate in the study, you will receive an auditory test that requires you to listen and respond to words or sentences presented via loudspeakers under different listening conditions. Your responses will be recorded using a written answer sheet. You will also complete two surveys about difficulties you experience in everyday listening situations.

You will then be assigned randomly (by chance) to one of three groups. All three groups receive identical hearing aids. For one group, the Placebo Group, however, the hearing aids are programmed to be minimally functional, only providing enough boost or amplification to make up for any loss of sound resulting from having something in your ears. That is, for the members of the Placebo Group, the boost provided by the hearing aids is insufficient to provide much help for your hearing loss.

For the other two groups, Group AB and Group CD, the hearing aids will provide boost to help compensate for your hearing loss, as is normally the case. For Group AB, the audiologist will be much more involved in selecting and fitting the hearing aids for you. For Group CD, you will be much more involved in selecting the hearing aids for yourself. After the hearing aids have been selected, you will be asked to participate in one follow-up session approximately 6 weeks later. At this session, you will repeat the listening test and surveys completed in the initial session prior to wearing hearing aids. You will also complete a third survey in writing at this time. Just like any person purchasing hearing aids in the U.S., you will then be given the option to return the hearing aids, if desired, and receive a full refund of the purchase price of the hearing aids, less any credits or payments made to you for completion of test sessions.

Those assigned randomly to the Placebo Group will be informed of their assignment at that time and will be given the opportunity to receive another 6-week trial of the hearing aids after they have been adjusted to provide enough boost to compensate for their hearing loss. If members of the Placebo Group elect to pursue another 6-week trial period and are dissatisfied with the hearing aids at the end of that additional 6-week trial period, they will then be given the option to return the hearing aids at that time, if desired, and receive a full refund of the purchase price of the hearing aids, less any credits or payments made to you for completion of test sessions.

In total, two sessions are required for this study. These sessions are designated as “Session 2” and “Session 3” in this study. Each session should require no more than two hours for completion.

Protocol 1111007504 IRB Approved

RISKS OF TAKING PART IN THE STUDY:

While in the study, the risks are minimal. If you have been randomly assigned to the Placebo Group, you may be frustrated at the lack of benefit provided by your hearing aids during the initial 6-week trial period. As noted, members of this group will have the opportunity for another 6-week trial period after the hearing aids have been adjusted appropriately to compensate for their hearing loss. For the Placebo Group, there is also potential risk of the hearing aid decreasing auditory awareness. The presence of the device in the subject’s ear canal may serve as a sound attenuator and make the participant less aware of sound. Steps have been taken to minimize this risk. First, the devices to be fitted are “open-fit” devices which minimize plugging up the ear canal. Second, the placebo devices will be fitted using real-ear measurements to verify that the hearing aids are acoustically transparent, neither amplifying nor attenuating incoming sounds.

For those in the CD and AB groups, it is possible that outcomes obtained will differ, being inferior for one of the groups. Regardless of outcome, the participants in either of these branches will be given the opportunity to receive services identical to those received by subjects in the other branch after the completion of Session 3 and without additional charges.

In addition, because hearing aids and eartips will be inserted into the subjects’ ears, there is some minimal risk for transmission of infection. This risk will be minimized by the thorough cleaning of all hearing aids and eartips tried by the patient after each subject’s use.

BENEFITS OF TAKING PART IN THE STUDY:

Outcomes of this investigation may aid hearing professionals in making decisions about how best to deliver hearing aids to adults with hearing loss.

ALTERNATIVES TO TAKING PART IN THE STUDY:

An alternative to participating in the study is to choose not to participate. If you do not participate, but are still interested in receiving hearing aids, you are welcome to do so at the IU Hearing Clinic, following normal clinical procedures, or elsewhere at your discretion. If requested, we will provide you with a copy of the results of your initial hearing test.

CONFIDENTIALITY

Efforts will be made to keep your personal information confidential. We cannot guarantee absolute confidentiality. Your personal information may be disclosed if required by law. Your identity will be held in confidence in reports in which the study may be published and databases in which results may be stored.

Organizations that may inspect and/or copy your research records for quality assurance and data analysis include groups such as the study investigator and his/her research associates, the IU Institutional Review Board or its designees, the study sponsor, the National Institutes of Health (NIH), and (as allowed by law) state or federal agencies, specifically the Office for Human Research Protections (OHRP) and the Food and Drug Administration (FDA), who may need to access your medical and/or research records.

PAYMENT

You will be paid $50 after the completion of Sessions 2 and 3 of this study for a total payment of $100. This amounts to

Protocol 1111007504 IRB Approved

16.7% of the $600 purchase price for your hearing aids. The first payment of $50 will be applied as a credit toward the $600 purchase price with the balance of $550 payable at the end of Session 2. The second payment, at the end of Session 3, will be issued to you as a check for $50, or a $50 credit will be applied to your credit card account..

CONTACTS FOR QUESTIONS OR PROBLEMS

For questions about the study or a research-related injury, contact the researcher, Larry Humes at [redacted] or by email at [redacted].

For questions about your rights as a research participant or to discuss problems, complaints or concerns about a research study, or to obtain information, or offer input, contact the IU Human Subjects Office at [redacted] or [redacted].

VOLUNTARY NATURE OF STUDY

Taking part in this study is voluntary. You may choose not to take part or may leave the study at any time. Leaving the study will not result in any penalty or loss of benefits to which you are entitled. Your decision whether or not to participate in this study will not affect your current or future relations with the investigator(s).

Your participation may be terminated by the investigator without regard to your consent in the following circumstances: If it is determined that you have significant earwax buildup that interferes with test procedures and you choose to not have it removed.

If you withdraw or are terminated from the study before completion, your partial data may be used for data analysis unless you request that it not be used.

COSTS

Taking part in this study may lead to added costs to you or your insurance company. You or your insurance company will be responsible for the following costs: Having your ears cleared of wax at your physician's office or location of your choosing. You will not be responsible for these study-specific costs: Having your ears cleared of wax by the audiologists working on the study.

SUBJECT'S CONSENT

In consideration of all of the above, I give my consent to participate in this research study.

I will be given a copy of this informed consent document to keep for my records. I agree to take part in this study.

Subject's Printed ______________________________ **Name:**

Subject's Signature:______________________________ ____________________
Date:

Protocol 1111007504 IRB Approved

(must be dated by the subject)

Printed Name of Person Obtaining Consent:___________________________________

Signature of Person Obtaining Consent: ______________________________________ **Date**:____________________

Protocol 1111007504 IRB Approved

Indiana University Clinical Trial Group
[redacted]

Medical Waiver

Waiver Statement or Physician Approval for Hearing Instrument Purchase

WAIVER STATEMENT:

I have been advised by the Indiana University Clinical Trial Group audiologists that the Food and Drug Administration has determined that my best health interest would be served if I had a medical evaluation by a licensed physician (preferably a physician who specializes in diseases of the ear) before purchasing a hearing aid. I do not wish a medical evaluation before purchasing a hearing aid/s.

Note: Purchaser must be 18 years of age or older to sign this waiver.

Exercise of this waiver is not in your best health interest and its use is strongly discouraged.

Name of Purchaser:____

Date: ________________________

PHYSICIAN APPROVAL STATEMENT

A medical evaluation was completed (must be within in the last six months) on

_______________________________, by ______________________________ and there are
Patient name Physician name

no medical contraindications for hearing aid use.

Physician signature:____

Date:________________________

SUBJECT #: _____________ TEST DATE: ________

CTC INITIALS: ________

Eligibility Part 2

Inclusion Criteria (all answers must be *yes* for entry into the study)

yes❐ no❐ 1. Subject is willing to purchase 2 hearing aids from IU Clinical Trial Group.
yes❐ no❐ 2. Subject is 'private-pay patient' without 3rd party reimbursement for hearing aid costs.
yes❐ no❐ 3. Subject has read, understood and signed the Medical Waiver form.
yes❐ no❐ 4. Subject has read, understood and signed the Informed Consent form.

Clinical Trial Coordinator's Signature Date signed

Session 2 Audiologist Checklist

AB GROUP SELECTION AND FITTING OF HEARING AIDS

___**Subject chooses color while audiologist chooses tube and dome size**

___**Generate first fitting and verify P1 is set appropriately:**

a. Fitting formula: NAL-NL2.
b. Fitting should default to "Experienced Non-Linear"
c. Select "Fitting" tab (top toolbar) and turn "Binaural Correction" OFF
d. Change Physical Properties to the size of dome and tube chosen.
e. Change P1 to **"Basic+Softswitching"** if it is not already on that setting.
   i. Directionality should be **FIXED**
   ii. Directional Mix: **Very Low**
   iii. DFS Ultra: **Moderate**
   iv. Expansion: **Off**
   v. Noise Tracker II: **Per Environment**
   vi. Wind Guard: **Off**
f. Click on "Environmental Optimizer" and click "Reset to…" then select "0 dB" so that all levels are at zero.
g. If applicable, click on "Tinnitus Sound Generator" and ensure it is OFF

___**Real Ear Measurement at 65dB SPL (using subject's RECD measured in Session 1) and fine tuning**

-Target-Matching Rule: for 250-4000Hz, all measured values within 4 dB of target at ALL frequencies. Will accept fit after initial fine tuning attempts if only within 7 dB of target at all frequencies. **(**If unable to meet ±7 dB criterion, subject will NOT be exited from study but will likely need to replace that subject in the study. FLAG file and let PI and CTC know.)

___**Real Ear Measurements following fine tuning: 55 dB SPL and 75 dB SPL (do not fine tune)**

**SII (S2MAP)**	**65**	**55**	**75**
**Right**			
**Left**			

___**MPO measurement at 85 dB SPL [**Instructions: "You will hear several loud beeps that get higher in pitch. The beeps will be loud, but if it gets uncomfortably loud, (refer to LDL chart), raise your hand. It is very quick, so try to raise your hand as quickly as possible. We can repeat if necessary."]

# times MPO adjusted (-4 dB increments)

	250	500	750	1000	1500	2000	3000	4000	6000
Right									
Left									

# times MPO adjusted (-2 dB increments)

	250	500	750	1000	1500	2000	3000	4000	6000
Both									

___Program VC based on HFPTA

HFPTA (1,2, &4kHz)	VC P1	VC P2	VC P3	VC P4
**≤21**	0	-2	-4	2
**22-29**	0	-3	-6	3
**30-38**	0	-4	-8	4
**39-47**	0	-5	-10	5
**48-55**	0	-6	-12	6
**56-64**	0	-7	-14	7
**≥ 65**	0	-8	-16	8

___Review beeps

___Real Ear Insertion Gain at 65 dB SPL AU (no fine tuning)

___Final Coupler Measurements (no fine tuning)

SII (S2SIMMAP)	65	55	75
**Right**			
**Left**			

___Print measurements to USB and record HA serial number, color, dome and tube size on light blue HA Decision Form for Audiologist

___Print R&L SIMMAP and give to CTC

0 GROUP SELECTION AND FITTING OF HEARING AIDS

___**Subject chooses color while audiologist chooses tube and dome size**

___**Generate first fitting and verify P1 is set appropriately:**

h. Fitting formula: NAL-NL2.
i. Fitting should default to “Experienced Non-Linear”
j. Select “Fitting” tab (top toolbar) and turn “Binaural Correction” OFF
k. Change Physical Properties to the size of dome and tube chosen.
l. Change P1 to **“Basic+Softswitching”** if it is not already on that setting.
   i. Directionality should be **OMNI**
   ii. DFS Ultra: **Moderate**
   iii. Expansion: **Off**
   iv. Noise Tracker II: **Per Environment**
   v. Wind Guard: **Off**
m. Click on “Environmental Optimizer” and click “Reset to…” then select “0 dB” so that all levels are at zero.

___**Real Ear Insertion Gain at 65 dB SPL AU (fine tune to get as close to zero as possible)**

___**Program VC**

- P1: +2dB
- P2:+1dB
- P3: 0
- P4: +3 dB

___**Final Coupler Measurements (no fine tuning)**

___**Review beeps**

**SII (S2SIMMAP)**	**65**	**55**	**75**
**Right**			
**Left**			

___**Print Insertion gain and final coupler measurements and bring folder to CTC office**

___**Sara administers HA orientation**

___**Print R&L SIMMAP and give to CTC**

Subject #:________________E Date:_____________

Number of additional people in room: ___________

Relationship(s) to subject:____

Hearing Aid Decision Form for Audiologist

RIGHT Hearing Aid:

Serial Number: __________________

Color: Grey Beige Brown

Tubing Length: 0 1 2 3

Dome Size: S M L Tulip

LEFT Hearing Aid:

Serial Number: __________________

Color: Grey Beige Brown

Tubing Length: 0 1 2 3

Dome Size: S M L Tulip

SUBJECT #: ___________E TEST DATE: ________

NUMBER OF ADDITIONAL PEOPLE IN ROOM: _______ EXAMINER'S INITIALS: ________

Orientation Checklist

FEATURES OF HEARING AID:
_____(1) User manual
_____(2) Warranty Information (3 years)
_____(3) Loss and damage (3 years/ $100 deductible)
_____(4) 6 week trial period

COMPONENTS AND FEATURES:
_____(1) Microphone
_____(2) Receiver
_____(3) Sound bore
_____(4) Program button for VC
_____(5) Battery door
_____(6) Thin tube
_____(7) Dome
_____(8) Anchor
_____(9) Left/Right indicator
_____(10) Difference between beeps

BATTERIES:
_____(1) Size (312/ brown)
_____(2) Type (Zinc-air)
_____(3) Cost (~$1/battery)
_____(4) Places to purchase
_____(5) Removal of tab for zinc air
_____(6) Battery life (5-7 days of typical use)
_____(7) Toxicity, keep away from children, pets and medication
_____(8) Open battery door when not in use
_____(9) Insertion and removal of battery (+vs-)
_____(10) PRACTICE

INSERTION AND REMOVAL OF AID:
_____(1) Right vs Left
_____(2) Turning aid on and off
_____(3) Do over a soft surface
_____(4) Orientation of aid in hand
_____(5) Comfort in ear
_____(6) PRACTICE

USE AND CARE OF AIDS:
_____(1) Brush and wire to clean thin tube and dome
_____(2) Wipe with soft cloth
_____(3) Avoid moisture and extreme temperature
_____(4) Do not use hair spray, cologne, etc with aid in ear
_____(5) Open battery door when not in use (helps prevent moisture damage) and store in case with lid open
_____(6) Keep hearing aids away from small children and pets
_____(7) Telephone use
_____(8) PRACTICE
_____(9) PRACTICE switching volume levels
_____(10) Initial use of aid
-Situations: quiet before noise; 1 on 1 before group situation
-Hours worn: Wear both aid s at least a **MINIMUM** of 4 hours/ day for the first week, increase by **AT LEAST** 2 hours/day every week until wearing them full time by follow-up appointment

HEARING AID ADJUSTMENT:
_____(1) Benefits/Limitations
_____(2) Discuss communication strategies
_____(3) Call CTC with questions during 6 week trial period

USER GUIDE

BEHIND-THE-EAR (BTE) MODELS: THIN TUBE

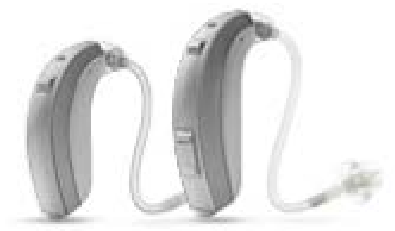

IU CT

A larger-print version of the *ReSound Alera 9* guide accompanying the trial hearing aids, with only minor changes, was used. Please see that guide for further details.

SUBJECT #: _______________ TEST DATE: _________

CTC'S INITIALS: ________

Hearing Aid Case Checklist for CTC

Big leather case ☐

- Hard case ☐
- Small Velcro pouch ☐
- Small zipper pouch ☐
  - Soft cloth ☐
  - Brush with wire loop and magnet ☐
  - Wire rod ☐
  - 2 extra domes ☐
- User guide ☐
- 2 packs of batteries ☐
- CTC's card ☐

Rx for ________________________________

	Right Hearing Aid	Left Hearing aid
Color	Beige ☐	Beige ☐
	Brown ☐	Brown ☐
	Grey ☐	Grey ☐
Tube	Size 0 ☐	Size 0 ☐
	Size 1 ☐	Size 1 ☐
	Size 2 ☐	Size 2 ☐
	Size 3 ☐	Size 3 ☐
Dome	Small ☐	Small ☐
	Medium ☐	Medium ☐
	Large ☐	Large ☐
	Tulip ☐	Tulip ☐
Model	X ☐	X ☐
	Y ☐	Y ☐
	Z ☐	Z ☐

Rx for ________________________________

	Right Hearing Aid	Left Hearing aid
Color	Beige ☐	Beige ☐
	Brown ☐	Brown ☐
	Grey ☐	Grey ☐
Tube	Size 0 ☐	Size 0 ☐
	Size 1 ☐	Size 1 ☐
	Size 2 ☐	Size 2 ☐
	Size 3 ☐	Size 3 ☐
Dome	Small ☐	Small ☐
	Medium ☐	Medium ☐
	Large ☐	Large ☐
	Tulip ☐	Tulip ☐
Model	X ☐	X ☐
	Y ☐	Y ☐
	Z ☐	Z ☐

Rx for ________________________________

	Right Hearing Aid	Left Hearing aid
Color	Beige ☐	Beige ☐
	Brown ☐	Brown ☐
	Grey ☐	Grey ☐
Tube	Size 0 ☐	Size 0 ☐
	Size 1 ☐	Size 1 ☐
	Size 2 ☐	Size 2 ☐
	Size 3 ☐	Size 3 ☐
Dome	Small ☐	Small ☐
	Medium ☐	Medium ☐
	Large ☐	Large ☐
	Tulip ☐	Tulip ☐
Model	X ☐	X ☐
	Y ☐	Y ☐
	Z ☐	Z ☐

Rx for ________________________________

	Right Hearing Aid	Left Hearing aid
Color	Beige ☐	Beige ☐
	Brown ☐	Brown ☐
	Grey ☐	Grey ☐
Tube	Size 0 ☐	Size 0 ☐
	Size 1 ☐	Size 1 ☐
	Size 2 ☐	Size 2 ☐
	Size 3 ☐	Size 3 ☐
Dome	Small ☐	Small ☐
	Medium ☐	Medium ☐
	Large ☐	Large ☐
	Tulip ☐	Tulip ☐
Model	X ☐	X ☐
	Y ☐	Y ☐
	Z ☐	Z ☐

Subject #:_________________E Date:_______________

Number of additional people in room: ____________

Relationship(s) to patient:____

Hearing Aid Decision Form for CTC

On a scale of 1-10, with 10 being the best, how helpful was the instructional video? ________

RIGHT Hearing Aid:

Hearing aid: X Y Z

Serial Number: _______________________

Color: Grey Beige Brown

Tubing Length: 0 1 2 3

Dome Size: S M L Tulip

LEFT Hearing Aid:

Hearing aid: X Y Z

Serial Number: _______________________

Color: Grey Beige Brown

Tubing Length: 0 1 2 3

Dome Size: S M L Tulip

NOT INTERESTED BIN

Hearing Aids

Color	Letter

Tubes and Tips

____

____

____

____

____

____

SUBJECT #: ___________E TEST DATE: ________

EXAMINER'S INITIALS: ________

As Worn Aided CST Form

TRACK 33

(CST 50 dB HL; +3 dB SNR; SP: 0 degrees N: 180 degrees; Binaural; Aided)

Text for the CONNECTED SPEECH TEST

*(scoring words are capitalized, mark through each incorrect response in ink with an **X**, and record the total number of words correct below)*

Passage: 1- UMBRELLA

The **NAME** "umbrella" means a small shadow.
Umbrellas **WERE** first used in **ANCIENT** Egypt.
THEY GAVE protection **FROM** the fierce **SUNSHINE**.
SLAVES held **UMBRELLAS** over their **MASTERS**.
In Egypt today, many people **CARRY** umbrellas.
In **EARLY** Rome, **ONLY WOMEN** used umbrellas.
IF a **MAN** did, he **WAS CONSIDERED** a sissy.
Umbrellas were **USED** by both **SEXES** in **ENGLAND**.
TODAY, people use umbrellas to keep **OUT** the **RAIN**.
Umbrellas **USED** as sunshades are called parasols.

Passage 2- GIRAFFE

The giraffe is the tallest wild **ANIMAL**.
It is three times taller than a man.
A full grown giraffe is eighteen **FEET** high.
The giraffe has an extremely **LONG NECK**.
The neck **HAS ONLY** seven **NECKBONES**.
The **GIRAFFE'S BODY** is about the **SIZE** of a **HORSE'S**.
The **BODY** is **SHAPED LIKE** a triangle.
Africa is the only country **WHERE** giraffes **LIVE WILD**.
LARGE GROUPS of them are **FOUND ON** the **PLAINS**.
They live there with **LIONS** and **ELEPHANTS**.

*** Total Number of CORRECTLY identified words: ____/49**

SUBJECT #: ____________ E TEST DATE: _________

EXAMINER INITIALS: ____________ *CHECK PAINT*

Hearing Aid Inspection Checklist

****Do not open battery door until after as worn speechmapping is complete****

	RIGHT		LEFT	
**Otoscopy/Ear exam**				
Hearing aid inserted correctly?	yes❒	no❒	yes❒	no❒
If no, describe: ____________				
Unremarkable?	yes❒	no❒	yes❒	no❒
EAC red?	yes❒	no❒	yes❒	no❒
Excessive cerumen?	yes❒	no❒	yes❒	no❒
Other: ____________	yes❒	no❒	yes❒	no❒

Notes: ____________

Hearing Aid

Tubing intact?	yes❒	no❒	yes❒	no❒
Tubing kinked?	yes❒	no❒	yes❒	no❒
Tubing blocked with cerumen?	yes❒	no❒	yes❒	no❒
Dome intact?	yes❒	no❒	yes❒	no❒
Dome blocked with cerumen?	yes❒	no❒	yes❒	no❒
Dome discolored?	yes❒	no❒	yes❒	no❒

RUN AS WORN SIMULATED SPEECH MAPPING

**SII (S3AWSIMMAP)**	**65**	**55**	**75**
**Right**			
**Left**			

Listening Check:

Current program **1 2 3 4** **1 2 3 4**

****Return hearing aid to program 1 after recording current program****

Acceptable sound quality? yes❒ no❒ yes❒ no❒

If no, describe:____________

Battery:

Sufficient charge? (≥ ¾ charge) yes❒ no❒ yes❒ no❒

SEE REVERSE SIDE TO COMPLETE

RIGHT LEFT

Was battery changed?	yes❐	no❐	yes❐	no❐
Was tubing changed?	yes❐	no❐	yes❐	no❐
Were domes changed?	yes❐	no❐	yes❐	no❐

RUN POST-MAINTENANCE SIMULATED SPEECH MAPPING

SII (S3PMSIMMAP)	65	55	75
Right			
Left			

Print R&L PM SIMMAP and give to CTC

SUBJECT #: ___________E TEST DATE: ________

EXAMINER'S INITIALS: ________

Post Maintenance Aided CST Form

TRACK 36

(CST 50 dB HL; +3 dB SNR; SP: 0 degrees N: 180 degrees; Binaural; Aided)

Text for the CONNECTED SPEECH TEST

(scoring words are capitalized, mark through each incorrect response in ink with an ***X****, and record the total number of words correct below)*

Passage: 1- NAIL

Nails are used to **FASTEN WOOD TOGETHER**.
Pioneers **USED WOODEN** pegs **INSTEAD** of nails.
One **END** of a nail is quite **POINTED**.
The **POINT** creates an **OPENING** for the **NAIL**.
It also helps **KEEP** the **WOOD** from **SPLITTING**.
At the nail's **OTHER** end is the **HEAD**.
It provides a **STRIKING SURFACE** for the hammer.
It also **COVERS** the nail **HOLE** in the wood.
There is a **SPECIAL NAIL** for every **PURPOSE**.
For **MOST** purposes a **ROUND** nail will do.

Passage 2- WOODPECKER

The woodpecker is a bird with a **STRONG BEAK**.
It bores **HOLES** in **TREES** looking for **INSECTS**.
Woodpeckers **LIVE** in all parts of the world.
The **TOES** of woodpeckers **ARE VERY UNUSUAL**.
Two **POINT FORWARD** and two face **BACKWARD**.
This allows the **BIRD** to cling to **TREES**.
The **TAIL FEATHERS** of a woodpecker are **STIFF**.
THEY can **USE** their tails as a **SUPPORT**.
They also use their tails to grasp **TREES**.
Woodpeckers **HAVE** long **TONGUES** with pointed **TIPS**.

*** Total Number of CORRECTLY identified words: ____/50**

Music/Training Questionnaire

SUBJECT #: ____________ E TEST DATE: _________

EXAMINER: _________

1. Have you been participating in any type of training (speech, auditory, cognitive, “brain-game”, etc.) outside of our studies?

_____ YES _____ NO

If yes, check any that subject mentions or write in answers as “other”.

_____ LACE

_____ Read My Quips

_____ SPATS

_____ Brain Training 101

_____ Brain Metrix

_____ Lumosity

_____ Other: ____

2. Have you ever received formal musical instruction (voice or instrument)?

_____ YES _____ NO

If yes, for how long ?________________

and how often? -----------------------------

3. Are you currently singing or playing an instrument at least 20 minutes 2 times per week?

_____ YES _____ NO

If yes, for how long have you been doing this? ___________________

SUBJECT #: ____________ E TEST DATE: _________

EXAMINER'S INITIALS: ____________

Final Hearing Aid Decision Form #1

1. Based on your experience over the past 6 weeks, are you going to keep the aids?

RIGHT: YES NO

LEFT: YES NO

If NO, ask why? (record choices for data entry, don't give choices to subject)

	**RIGHT**	**LEFT**
Can't hear in noise	______	_______
Can't hear on phone	______	_______
Can't get used to sound	______	_______
Feedback	______	_______
Uncomfortable	______	_______
Costs too much	______	_______
No benefit	______	_______

Other: ____

2. Did you read the user guide that was sent home with the hearing aids? YES NO

    a. On a scale of 1 (least) to 10 (most), how helpful was the guide? _______

3. Regardless of answer, get folder from CTC.

4. Do data logging and record on data logging form.

Regardless of YES/NO answer on keeping hearing aids:

CD: You were in the CD group which is the group that chose your own hearing aids. These hearing aids were programmed for common hearing losses but were not programmed specifically for your hearing loss. You also did not receive any instruction from an audiologist about the use, care and maintenance of your hearing aids. Would you be interested in having the hearing aids re-programmed for your loss, receiving instruction on the use and care of them and trying them for another 4 weeks at no additional cost to you? You would still have the opportunity to return them for a full refund after the 4 weeks is over.
IF YES to another trial period, provide orientation to subject.
See CTC to schedule Session 3B.
IF NO, see CTC for refund.

PLACEBO: You were in the placebo group which means your hearing aids were programmed so that they did not function like real hearing aids and did not provide any benefit for your hearing loss. Would you be interested in having your hearing aids re-programmed for your hearing loss and trying them for another 4 weeks at no additional cost to you? You would still have the opportunity to return them for a full refund after the 4 weeks is over.
IF YES to another trial period, see CTC to schedule Session 3B.
IF NO, see CTC for refund.

AB/yes keeping aids: You were in the group who received audiology best practices and were fitted with your hearing aids by an audiologist. The only thing we did not do was offer fine-tuning of your hearing aid programming during the trial period. If you’re not having any problems then we’ll see you in one year for a hearing aid check-up.
See CTC to schedule 1 year check-up.

AB/not keeping aids: You were in the group who received audiology best practices and were fitted with your hearing aids by an audiologist. The only thing we did not do was offer fine-tuning of your hearing aids during the trial period. Would you be interested in having your hearing aids adjusted and trying them for another 4 weeks at no additional cost to you? You would still have the opportunity to return them for a full refund after the 4 weeks is over.
IF YES to another trial period, see CTC to schedule Session 3B.
IF NO, see CTC for a refund.

SUBJECT #: ____________ E **TEST DATE: _________**

Data Logging Form

EXAMINER: ________

	**RIGHT**	**LEFT**
**Total # hours used:**	__________	__________
**Average hours used per day:**	__________	__________

Session 3 Checklist

For all groups: if DFS must be run in addition to programming changes, follow this order:

1) Programming changes; **2)** Run and save PASIMMAP xml; **3)** Run DFS; **4)** Run and save POSTDFS SIMMAP xml.

If cannot follow order, save 'POSTDFS+PASIMMAP' screenshots and normal post DFS xml. PASIMMAP will be 999, POSTDFS xml will be pulled.

_______ 1. aided phap
_______ 2. aided hhie
_______ 3. HASS
_______ 4. put aids in
_______ 5. aided cst
_______ 6. Phast-r (phone, batteries, cleaning tools)
_______ 7. Music questionnaire
_______ 8. HA Decision Form #1
_______ 9. Take tablet to CTC and get subj folder
_______ 10. Discuss/reveal group
_______ 11. Datalogging, print to CTC office and reset (do CD & placebo datalogging without audiogram or without new one; for CD, create file with subj #, read HA, ?Target-No, Keep gain…)
_______ 12. AB-keeping with fine-tuning: open subject's file in Aventa , connect aids, fine-tune, run coupler msmts, print table to USB, save session and to aids. Save XML file to USB. Take to CTC to schedule 3B.

SII (S3PASIMMAP)	65	55	75
Right			
Left			

_______ 13. AB-keeping with no fine-tuning: Run DFS, run coupler msmts-print table to USB, save XML file to USB, take to CTC to discuss 1 year follow-up

SII (S3POSTDFSSIMMAP)	65	55	75
Right			
Left			

_______ 14. AB-not keeping and no adj wanted: take to CTC to process refund, Datalogging
_______ 15. Placebo: Open subject's file in Aventa, change audiogram on screen before clicking Aventa button, SAVE, click Aventa button, will ask if I want to use new audiogram when connecting, click yes and new settings will be calculated. Check that all settings and programs correspond to appropriate protocol settings.

_______ 16. Verify P1 is set appropriately:

i. Fitting formula: **NAL-NL2**
ii. Fitting should default to "**Experienced Non-Linear**"
iii. Select "Fitting" tab (top toolbar) and assure "**Binaural Correction**" is **OFF**
iv. Assure **Physical Properties** is set to the actual size of dome and tube
v. Assure **P1** is "**Basic+Softswitching**":
   1. Directionality should be **FIXED**
   2. Directional Mix: **Very Low**
   3. DFS Ultra: **Moderate**
   4. Expansion: **Off**
   5. Noise Tracker II: **Per Environment**
   6. Wind Guard: **Off**
vi. Click on "**Environmental Optimizer**" and "**Reset to…**" then select "**0 dB**" so that all levels are at zero.
vii. If applicable, click on "**Tinnitus Sound Generator**" and ensure it is **OFF**

- Get patient and position for real-rear testing.
- RECD will already be entered
- Go to Tests, On-Ear measures, speechmapping
- Insert probe tubes in ears
- MUTE hearing aids prior to insertion
- Start with RIGHT
- Choose EQUALIZE while aids muted, after white noise stops, UNMUTE RIGHT aid
- Run speechmapping on RIGHT at 65, adjust as needed. Once targets matched, run 55 & 75.

**SII (S3PAMAP)**	**65**	**55**	**75**
**Right**			
**Left**			

- Print results in table format to USB.
- Repeat for LEFT starting with EQUALIZE.

_______ 17. CD: same as placebo except will need to create file in aventa and enter audiogram for first time.

_______ 18. After speechmapping is complete, run Loudness measures.

_______ 19. Familiarize with loudness categories.

_______ 20. Change to graph view on Verifit to watch where subject raises hand.

_______ 21. Run MPO at 85 with RIGHT first (left muted). (decrease at appr freq by 4 dB).

_______ 22. Do same for LEFT (right muted).

# times MPO adjusted (-4 dB increments)

	250	500	750	1000	1500	2000	3000	4000	6000
Right									
Left									

_______ 23. Do loudness measures for both ears (decrease at appr freq by 2 dB in both aids).

# times MPO adjusted (-2 dB increments)

	250	500	750	1000	1500	2000	3000	4000	6000
Both									

_______ 24. Program VC based on HFPTA (copy p1 to other programs and adjust gain).

HFPTA (1,2, &4kHz)	VC P1	VC P2	VC P3	VC P4
**≤21**	0	-2	-4	2
**22-29**	0	-3	-6	3
**30-38**	0	-4	-8	4
**39-47**	0	-5	-10	5
**48-55**	0	-6	-12	6
**56-64**	0	-7	-14	7
**≥ 65**	0	-8	-16	8

_______ 25. Save to session and aid.

_______ 26. Remove from ears, run each aid with simulated speechmapping to get use curves.

SII (S3PASIMMAP)	65	55	75
**Right**			
**Left**			

_______ 27. Print results in table format to USB, save XML file to USB.

_______ 28. Complete HA Status Form #1.

_______ 29. Placebo or CD with re-programming, take to CTC to schedule 3B.

SUBJECT #: ____________ E TEST DATE: _________

Hearing Aid Adjustment Log

EXAMINER: ________

RIGHT:

LEFT:

SUBJECT #: ____________ E TEST DATE: _________

SESSION 3 EXAMINER: _________

Hearing Aid Questions Form

1. ____
2. ____
3. ____
4. ____
5. ____
6. ____
7. ____
8. ____
9. ____
10. ____

SUBJECT #: ____________ E TEST DATE: _________

EXAMINER'S INITIALS: ____________

Hearing Aid Status Form #1

**Status of HAs:**	**RIGHT**	**LEFT**
Kept Study Aid(s) as-is	________	________
Kept study aids and requested adjustments/reprogramming	________	________
Returned study aids	________	________

SUBJECT #: ____________E TEST DATE: ________

EXAMINER'S INITIALS: ________

Final Exit Form #2

Status of Subject:

❒ Discontinued (Complete Section A)

❒ Completed Study and elected to keep hearing aids

❒ Completed Study and is returning hearing aids

Section A: Reason for Subject Exit

Development of Any Exclusion Criteria:

❒ Cognitive/Language-Based Condition

❒ Medical/Surgical Ear Condition

❒ Shape of External Ear Canal

❒ Fluctuating Hearing Loss

❒ Participant in other hearing aid related Clinical Trials

❒ Disease/Condition Affecting Hearing/Cognition

❒ Medication Affecting Hearing/Cognition

❒ VIII Nerve Tumor

Development of Any Study-Related Medical Conditions:

❒ Allergic Reaction

❒ Accumulation of Ear Wax

❒ Infection

❒ Adverse Effect

Miscellaneous:

❒ Inability/Unwillingness to Maintain Wearing Schedule

❒ Relocation of Subject

❒ Over 3 Weeks Cumulative Off The Ear Time

❒ Threshold Change by More Than 10 dB

❒ Lost to Follow-Up

❒ Death or Disability

❒ Failure to Follow Instructions

❒ Lack of Motivation

❒ Inability/Unwillingness to Participate

❒ Other ______________________

____

Investigator's Signature Date Signed

SUBJECT #: ___________E TEST DATE: ________

EXAMINER'S INITIALS: ________

As Worn Aided CST Form

TRACK 40

(CST 50 dB HL; +3 dB SNR; SP: 0 degrees N: 180 degrees; Binaural; Aided)

Text for the CONNECTED SPEECH TEST

*(scoring words are capitalized, mark through each incorrect response in ink with an **X**, and record the total number of words correct below)*

Passage: 1- DONKEY

Donkeys are **SMALLER**, sturdier relatives of **HORSES**.
The **WILD** donkey is **SHAPED** like a **ZEBRA**.
It is four **FEET** high at the **SHOULDERS**.
The donkey's **COAT** is **GRAY** and black.
It **HAS** a **DARK LINE** along its **BACK**.
This **ANIMAL** is **EXTREMELY INTELLIGENT**.
SURPRISINGLY, it is also a **SWIFT RUNNER**.
Man has **TAMED** donkeys for his personal use.
Donkeys are **OFTEN** used as **BEASTS** of burden.
All donkeys are **NOTED** for their **HUGE EARS**.

Passage 2- GUITAR

The guitar is a stringed **MUSICAL INSTRUMENT**.
Guitars are used to **ACCOMPANY SINGING**.
They are played in **GROUPS** with other **INSTRUMENTS**.
A **POPULAR** style of guitar **HAS** a flat top.
It is made of wood and **HAS** six **STRINGS**.
You **TUNE** a guitar **BY** comparing **OCTAVE NOTES**.
The **FINGERBOARD** is **HELD** with the **LEFT** hand.
The **MUSICIAN'S** right hand **PULLS** the strings.
He plays **BASS NOTES** with his **RIGHT** thumb.
OTHER notes are **PLAYED** with the first **THREE** fingers.

*** Total Number of CORRECTLY identified words: ____/50**

SUBJECT #: ____________ E TEST DATE: _________

EXAMINER INITIALS: ____________ *CHECK PAINT*

Hearing Aid Inspection Checklist

****Do not open battery door until after as worn speechmapping is complete****

	RIGHT		LEFT	
**Otoscopy/Ear exam**				
Hearing aid inserted correctly?	yes❒	no❒	yes❒	no❒
If no, describe: ________________				
Unremarkable?	yes❒	no❒	yes❒	no❒
EAC red?	yes❒	no❒	yes❒	no❒
Excessive cerumen?	yes❒	no❒	yes❒	no❒
Other: ___________________	yes❒	no❒	yes❒	no❒

Notes: ____

Hearing Aid

	RIGHT		LEFT	
Tubing intact?	yes❒	no❒	yes❒	no❒
Tubing kinked?	yes❒	no❒	yes❒	no❒
Tubing blocked with cerumen?	yes❒	no❒	yes❒	no❒
Dome intact?	yes❒	no❒	yes❒	no❒
Dome blocked with cerumen?	yes❒	no❒	yes❒	no❒
Dome discolored?	yes❒	no❒	yes❒	no❒

RUN AS WORN SIMULATED SPEECH MAPPING

SII (S3BAWSIMMAP)	65	55	75
Right			
Left			

Listening Check:

	RIGHT				LEFT			
Current program	**1**	**2**	**3**	**4**	**1**	**2**	**3**	**4**

****Return hearing aid to program 1 after recording current program****

	RIGHT		LEFT	
Acceptable sound quality?	yes❒	no❒	yes❒	no❒

If no, describe:____

Battery:

	RIGHT		LEFT	
Sufficient charge? (≥ ¾ charge)	yes❒	no❒	yes❒	no❒

SEE REVERSE SIDETO COMPLETE

RIGHT LEFT

Was battery changed? yes❐ no❐ yes❐ no❐

Was tubing changed? yes❐ no❐ yes❐ no❐

Were domes changed? yes❐ no❐ yes❐ no❐

RUN POST MAINTENANCE SIMULATED SPEECH MAPPING

SII (S3BPMSIMMAP)	65	55	75
Right			
Left			

****Print R&L PM SIMMAP and give to CTC****

SUBJECT #: ____________ TEST DATE: ________

EXAMINER'S INITIALS: ________

Post Maintenance Aided CST Form

TRACK 51

(CST 50 dBHL; +3 dB SNR; SP: 0 degrees N: 180 degrees; Binaural; Aided)

Text for the CONNECTED SPEECH TEST

*(scoring words are capitalized, mark through each incorrect response in ink with an **X**, and record the total number of words correct below)*

Passage: 1- EAR

The ear is an important **SENSE ORGAN**.
The ear **HAS** two main **PURPOSES**.
It lets **MAN HEAR** and **MAINTAIN** his balance.
GOOD hearing permits **PEOPLE** to understand **SPEECH**.
Through speech, we **EXCHANGE** ideas and **OPINIONS**.
HEARING ALSO makes man **AWARE** of **DANGER**.
The ear's **BALANCE** mechanism helps us walk **UPRIGHT**.
DAMAGE to this section causes **STAGGERING**.
The **PERSON** also **GETS** disoriented and **DIZZY**.
This kind of dizziness is **CALLED VERTIGO**.

Passage 2- LIVER

The liver is a very important **INTERNAL** organ.
Its **MAIN FUNCTION** is to filter the **BLOOD**.
The liver is the **LARGEST** organ in **MAN**.
It can **WEIGH** three to four **POUNDS**.
The liver is **DARK RED** or **CHOCOLATE** colored.
It is **LOCATED** in the **MIDDLE SECTION** of the **BODY**.
It **FITS** closely to the intestines and kidneys.
It is **POSSIBLE** to **TRANSPLANT** a liver.
This **ADVANCED** operation is **VERY** expensive.
HOWEVER, it is **RESPONSIBLE** for **SAVING MANY LIVES**.

*** Total Number of CORRECTLY identified words: ____/50**

Music/Training Questionnaire

SUBJECT #: ____________ E TEST DATE: _________

EXAMINER: ___________

1. Have you been participating in any type of training (speech, auditory, cognitive, "brain-game", etc.) outside of our studies?

_____ YES _____ NO

If yes, check any that subject mentions or write in answers as "other".

_____ LACE

_____ Read My Quips

_____ SPATS

_____ Brain Training 101

_____ Brain Metrix

_____ Lumosity

_____ Other: ____

2. Have you received formal musical instruction (voice or instrument) since your last visit to the lab?

_____ YES _____ NO

If yes, for how long ?________________

and how often? ------------------------------

3. Are you currently singing or playing an instrument at least 20 minutes 2 times per week?

_____ YES _____ NO

If yes, for how long have you been doing this? ___________________

SUBJECT #: ____________ E TEST DATE: _________

Hearing Aid Decision Form #2

EXAMINER: __________

1. Based on your experience over the past 4 weeks, are you going to keep the aids?

RIGHT: YES NO

LEFT: YES NO

If NO, ask why? (record choices for data entry, don't give choices to subject)

	**RIGHT**	**LEFT**
Can't hear in noise	______	_______
Can't hear on phone	______	_______
Can't get used to sound	______	_______
Feedback	______	_______
Uncomfortable	______	_______
Costs too much	______	_______
No benefit	______	_______

Other: ____

3. Do datalogging

Fill out HA Status #2, return to CTC for either refund or 1 yr follow up scheduling.

SUBJECT #: ____________ E **TEST DATE: _________**

Data Logging Form

EXAMINER: __________

	**RIGHT**	**LEFT**
**Total # hours used:**	__________	__________
**Average hours used per day:**	__________	__________

For all groups: if DFS must be run in addition to programming changes, follow this order:

1) Programming changes; **2)** Run and save PASIMMAP xml; **3)** Run DFS; **4)** Run and save POSTDFS SIMMAP xml.

If cannot follow order, save 'POSTDFS+PASIMMAP' screenshots and normal post DFS xml. PASIMMAP will be 999, POSTDFS xml will be pulled.

Session 3B checklist

_______ 1. aided phap
_______ 2. aided hhie
_______ 3. HASS
_______ 4. put aids in
_______ 5. aided cst
_______ 6. Phast-r (phone, batteries, cleaning tools)
_______ 7. Music questionnaire
_______ 8. HA Decision Form #2
_______ 9. Keeping with fine-tuning:
   a. Do Datalogging, print to CTC office and reset
   b. Fine-tune, run & save PASIMMAP coupler msmts (screenshots and XML to usb). Then run DFS, run & save POSTDFS coupler msmts (screenshots and XML to usb).

**SII (S3BPASIMMAP)**	**65**	**55**	**75**
**Right**			
**Left**			

   c. take to CTC to discuss 1 year follow-up

_______ 10. Keeping with NO fine-tuning:
   a. Do Datalogging, print to CTC office and reset
   b. RUN DFS AND RUN FINAL COUPLER MSMTS. Print each simmap to USB. When all done, save XML file to USB.

**SII (S3BPOSTDFSSIMMAP)**	**65**	**55**	**75**
**Right**			
**Left**			

   c. take to CTC to discuss 1 year follow-up

_______ 11. If not keeping, take to CTC for refund. Remember to then do Datalogging.
_______ 12. Complete HA Status Form #2.
_______ 13. Print R&L PM SIMMAP and give to CTC.

VC Programming based on HFPTA

HFPTA (1,2, &4kHz)	VC P1	VC P2	VC P3	VC P4
**≤21**	0	-2	-4	2
**22-29**	0	-3	-6	3
**30-38**	0	-4	-8	4
**39-47**	0	-5	-10	5
**48-55**	0	-6	-12	6
**56-64**	0	-7	-14	7
**≥ 65**	0	-8	-16	8

SUBJECT #: ____________ E TEST DATE: _________

Hearing Aid Adjustment Log

EXAMINER: ___________

RIGHT:

LEFT:

SUBJECT #: ____________ E TEST DATE: _________

SESSION 3B EXAMINER: _________

Hearing Aid Questions Form

1. ____
2. ____
3. ____
4. ____
5. ____
6. ____
7. ____
8. ____
9. ____
10. ____

SUBJECT #: ____________ E TEST DATE: _________

EXAMINER'S INITIALS: ____________

Hearing Aid Status Form #2 (do at end of session 3b)

**Status of HAs:**	**RIGHT**	**LEFT**
Kept Study Aid(s) as-is	________	________
Kept study aids and requested adjustments/reprogramming	________	________
Returned study aids	________	________

Please note that the next several pages, labeled T-x, are hard-copy text images of information for several surveys that were actually administered and scored via a Windows-7 tablet PC. The formatting of the text on the screen and the actual information presented differed from that shown in the pages to follow. Please see the manuscript for reference citations for these surveys and tests.

ABCD Study- PHAP UNAIDED- Session 2

SUBJECT #: _______________ TEST DATE: __________

EXAMINER'S INITIALS: _________

PHAP Instructions: Please answer each question based on your listening experiences recently.

Please circle the answer that comes closest to your everyday experience. Notice that each choice includes a percentage. You can use this to help you decide on your answer. For example if the statement is true about 75% of the time, circle C for that item. If you have not experienced a particular situation, imagine how you would respond in a similar situation.

A = Always (99%)	D = Half-the-time (50%)	G = Never (1%)
B = Almost always (87%)	E = Occasionally (25%)	
C = Generally (75%)	F = Seldom (12%)	

1. I can understand others in a small group situation if there is no noise.

   A B C D E F G

2. When I am listening to a speaker who is talking to a large group, and I am seated toward the rear of the room, I must make an effort to listen.

   A B C D E F G

PHAP Instructions: Please answer each question based on your listening experiences recently.

Please circle the answer that comes closest to your everyday experience. Notice that each choice includes a percentage. You can use this to help you decide on your answer. For example if the statement is true about 75% of the time, circle C for that item. If you have not experienced a particular situation, imagine how you would respond in a similar situation.

A = Always (99%)	D = Half-the-Time (50%)	G = Never (1%)
B = Almost Always (87%)	E = Occasionally (25%)	
C = Generally (75%)	F = Seldom (12%)	

3. Women's voices sound "shrill."

A B C D E F G

4. I find that most people speak too softly.

A B C D E F G

5. I have trouble comprehending speech when I am in a busy department store talking with the clerk.

A B C D E F G

6. I can understand my family when they speak softly to me.

A B C D E F G

PHAP Instructions: Please answer each question based on your listening experiences recently.

Please circle the answer that comes closest to your everyday experience. Notice that each choice includes a percentage. You can use this to help you decide on your answer. For example if the statement is true about 75% of the time, circle C for that item. If you have not experienced a particular situation, imagine how you would respond in a similar situation.

A = Always (99%)	D = Half-the-Time (50%)	G = Never (1%)
B = Almost Always (87%)	E = Occasionally (25%)	
C = Generally (75%)	F = Seldom (12%)	

7. I can understand a speaker in a small group, even when those around us are speaking softly to each other.

A B C D E F G

8. I can understand conversations even when several people are talking.

A B C D E F G

9. When the telephone rings, the sound startles me.

A B C D E F G

10. I have to ask people to repeat themselves when there is background noise.

A B C D E F G

PHAP Instructions: Please answer each question based on your listening experiences recently.

Please circle the answer that comes closest to your everyday experience. Notice that each choice includes a percentage. You can use this to help you decide on your answer. For example if the statement is true about 75% of the time, circle C for that item. If you have not experienced a particular situation, imagine how you would respond in a similar situation.

A = Always (99%)	D = Half-the-Time (50%)	G = Never (1%)
B = Almost Always (87%)	E = Occasionally (25%)	
C = Generally (75%)	F = Seldom (12%)	

11. When I am in a crowded grocery store, talking with the cashier, I can follow the conversation.

A B C D E F G

12. When I am having a conversation, and people are talking quietly nearby, I have to strain to understand the speaker.

A B C D E F G

13. If a car horn sounds, it makes me jump.

A B C D E F G

14. When I am talking to a group and someone from the back of the room asks a question, I have to ask someone up front to repeat the question.

A B C D E F G

PHAP Instructions: Please answer each question based on your listening experiences recently.

Please circle the answer that comes closest to your everyday experience. Notice that each choice includes a percentage. You can use this to help you decide on your answer. For example if the statement is true about 75% of the time, circle C for that item. If you have not experienced a particular situation, imagine how you would respond in a similar situation.

A = Always (99%)	D = Half-the-Time (50%)	G = Never (1%)
B = Almost Always (87%)	E = Occasionally (25%)	
C = Generally (75%)	F = Seldom (12%)	

15. The sounds of construction work are uncomfortably loud.

A B C D E F G

16. When I am in a crowded reception room waiting to be called, I miss hearing my name.

A B C D E F G

17. When I am having a quiet conversation with a companion, I have difficulty understanding.

A B C D E F G

18. When I am listening to the news on the car radio, and family members are talking, I have trouble hearing the news.

A B C D E F G

PHAP Instructions: Please answer each question based on your listening experiences recently.

Please circle the answer that comes closest to your everyday experience. Notice that each choice includes a percentage. You can use this to help you decide on your answer. For example if the statement is true about 75% of the time, circle C for that item. If you have not experienced a particular situation, imagine how you would respond in a similar situation.

A = Always (99%)	D = Half-the-Time (50%)	G = Never (1%)
B = Almost Always (87%)	E = Occasionally (25%)	
C = Generally (75%)	F = Seldom (12%)	

19. The sound of screeching tires is uncomfortably loud.

A B C D E F G

20. I can understand conversation when I am walking with a friend through a quiet park.

A B C D E F G

21. The sound of a fire engine siren close by is so loud that I need to cover my ears.

A B C D E F G

22. When I am in conversation with someone across a large empty room (such as an auditorium), I understand the words.

A B C D E F G

PHAP Instructions: Please answer each question based on your listening experiences recently.

Please circle the answer that comes closest to your everyday experience. Notice that each choice includes a percentage. You can use this to help you decide on your answer. For example if the statement is true about 75% of the time, circle C for that item. If you have not experienced a particular situation, imagine how you would respond in a similar situation.

A = Always (99%)	D = Half-the-Time (50%)	G = Never (1%)
B = Almost Always (87%)	E = Occasionally (25%)	
C = Generally (75%)	F = Seldom (12%)	

23. When I am on a busy street, asking someone for directions, I have to ask him to repeat them before I really understand.

A B C D E F G

24. The sound of running water, such as a toilet or shower, is uncomfortably loud.

A B C D E F G

25. When a speaker is addressing a small group, and everyone is listening quietly, I have to strain to understand.

A B C D E F G

26. I have trouble understanding dialogue in a movie or at the theater.

A B C D E F G

PHAP Instructions: Please answer each question based on your listening experiences recently.

Please circle the answer that comes closest to your everyday experience. Notice that each choice includes a percentage. You can use this to help you decide on your answer. For example if the statement is true about 75% of the time, circle C for that item. If you have not experienced a particular situation, imagine how you would respond in a similar situation.

A = Always (99%)	D = Half-the-Time (50%)	G = Never (1%)
B = Almost Always (87%)	E = Occasionally (25%)	
C = Generally (75%)	F = Seldom (12%)	

27. When I am in a crowd with a friend who doesn't want others to overhear our conversation, I have trouble hearing as well.

A B C D E F G

28. When I am at the dinner table with several people, and am trying to have a conversation with one person, understanding speech is difficult.

A B C D E F G

29. When I'm in a quiet conversation with my doctor in an examination room, it is hard to follow the conversation.

A B C D E F G

30. I have trouble understanding others when an air conditioner or fan is on.

A B C D E F G

PHAP Instructions: Please answer each question based on your listening experiences recently.

Please circle the answer that comes closest to your everyday experience. Notice that each choice includes a percentage. You can use this to help you decide on your answer. For example if the statement is true about 75% of the time, circle C for that item. If you have not experienced a particular situation, imagine how you would respond in a similar situation.

A = Always (99%)	D = Half-the-Time (50%)	G = Never (1%)
B = Almost Always (87%)	E = Occasionally (25%)	
C = Generally (75%)	F = Seldom (12%)	

31. I miss a lot of information when I'm listening to a lecture.

A B C D E F G

32. I can understand my family when they talk to me a normal voice.

A B C D E F G

33. I have to ask people to repeat themselves in one-on-one conversation in a quiet room.

A B C D E F G

34. I have difficulty hearing conversation when I'm with one other person at home.

A B C D E F G

PHAP Instructions: Please answer each question based on your listening experiences recently.

Please circle the answer that comes closest to your everyday experience. Notice that each choice includes a percentage. You can use this to help you decide on your answer. For example if the statement is true about 75% of the time, circle C for that item. If you have not experienced a particular situation, imagine how you would respond in a similar situation.

A = Always (99%)	D = Half-the-Time (50%)	G = Never (1%)
B = Almost Always (87%)	E = Occasionally (25%)	
C = Generally (75%)	F = Seldom (12%)	

35. When I am riding in the back seat of a car, and the driver talks to me from the front, I have to strain to understand.

A B C D E F G

36. Music sounds distorted to me.

A B C D E F G

37. When I'm talking with the teller at the drive-in window of my bank, I understand the speech coming from the loudspeaker.

A B C D E F G

38. When I am in a small office, interviewing or answering questions, I have difficulty following the conversation.

A B C D E F G

PHAP Instructions: Please answer each question based on your listening experiences recently.

Please circle the answer that comes closest to your everyday experience. Notice that each choice includes a percentage. You can use this to help you decide on your answer. For example if the statement is true about 75% of the time, circle C for that item. If you have not experienced a particular situation, imagine how you would respond in a similar situation.

A = Always (99%)	D = Half-the-Time (50%)	G = Never (1%)
B = Almost Always (87%)	E = Occasionally (25%)	
C = Generally (75%)	F = Seldom (12%)	

39. When a lecturer is giving instructions, I can easily follow along.

A B C D E F G

40. Everyday sounds are too soft for me to hear clearly.

A B C D E F G

41. I avoid using certain appliances (blender, vacuum cleaner, etc.) because their loudness is uncomfortable.

A B C D E F G

42. When I am in a busy restaurant and the waitress is taking my order, I can comprehend her questions.

A B C D E F G

PHAP Instructions: Please answer each question based on your listening experiences recently.

Please circle the answer that comes closest to your everyday experience. Notice that each choice includes a percentage. You can use this to help you decide on your answer. For example if the statement is true about 75% of the time, circle C for that item. If you have not experienced a particular situation, imagine how you would respond in a similar situation.

A = Always (99%) D = Half-the-Time (50%) G = Never (1%)

B = Almost Always (87%) E = Occasionally (25%)

C = Generally (75%) F = Seldom (12%)

43. I avoid crowds because the noise is uncomfortably loud.

A B C D E F G

44. When I am at a large, noisy party, conversation is very confusing.

A B C D E F G

45. When I am in a theater watching a movie or play, and the people around me are whispering and rustling paper wrappers, I can still make out the dialogue.

A B C D E F G

46. When I am in a quiet restaurant, I can understand soft conversation.

A B C D E F G

PHAP Instructions: Please answer each question based on your listening experiences recently.

Please circle the answer that comes closest to your everyday experience. Notice that each choice includes a percentage. You can use this to help you decide on your answer. For example if the statement is true about 75% of the time, circle C for that item. If you have not experienced a particular situation, imagine how you would respond in a similar situation.

A = Always (99%)	D = Half-the-Time (50%)	G = Never (1%)
B = Almost Always (87%)	E = Occasionally (25%)	
C = Generally (75%)	F = Seldom (12%)	

47. I can’t understand the TV news when the volume is set by a normal-hearing person.

A B C D E F G

48. I can understand conversation during a quiet dinner with my family.

A B C D E F G

49. When I am listening to the news on my car radio, and the car windows are closed, I understand the words.

A B C D E F G

50. The sound quality of music isn't very good.

A B C D E F G

PHAP Instructions: Please answer each question based on your listening experiences recently.

Please circle the answer that comes closest to your everyday experience. Notice that each choice includes a percentage. You can use this to help you decide on your answer. For example if the statement is true about 75% of the time, circle C for that item. If you have not experienced a particular situation, imagine how you would respond in a similar situation.

A = Always (99%)	D = Half-the-Time (50%)	G = Never (1%)
B = Almost Always (87%)	E = Occasionally (25%)	
C = Generally (75%)	F = Seldom (12%)	

51. The ring of a telephone sounds "tinny."

A B C D E F G

52. I understand the newscaster when I am watching TV news at home alone.

A B C D E F G

53. I can follow the words of a sermon when listening to a religious service.

A B C D E F G

54. When I am at home, talking with someone who is in another room, following the conversation is difficult.

A B C D E F G

PHAP Instructions: Please answer each question based on your listening experiences recently.

Please circle the answer that comes closest to your everyday experience. Notice that each choice includes a percentage. You can use this to help you decide on your answer. For example if the statement is true about 75% of the time, circle C for that item. If you have not experienced a particular situation, imagine how you would respond in a similar situation.

A = Always (99%)	D = Half-the-Time (50%)	G = Never (1%)
B = Almost Always (87%)	E = Occasionally (25%)	
C = Generally (75%)	F = Seldom (12%)	

55. Unexpected sounds like a smoke detector or alarm bell are uncomfortable.

A B C D E F G

56. When I'm talking with a friend outdoors on a windy day, I miss a lot of the conversation.

A B C D E F G

57. Everyday sounds that don't bother others are too loud for me.

A B C D E F G

58. It's hard for me to understand what is being said at lectures or church services.

A B C D E F G

PHAP Instructions: Please answer each question based on your listening experiences recently.

Please circle the answer that comes closest to your everyday experience. Notice that each choice includes a percentage. You can use this to help you decide on your answer. For example if the statement is true about 75% of the time, circle C for that item. If you have not experienced a particular situation, imagine how you would respond in a similar situation.

A = Always (99%)	D = Half-the-Time (50%)	G = Never (1%)
B = Almost Always (87%)	E = Occasionally (25%)	
C = Generally (75%)	F = Seldom (12%)	

59. When I am in a room with the door closed and I want to over-hear a conversation going on outside the door, I have to strain to listen.

A B C D E F G

60. People's voices sound unnatural.

A B C D E F G

61. When I am in face-to-face conversation with one member of my family, I can easily follow along.

A B C D E F G

62. When I am in a meeting with several other people, I can comprehend speech.

A B C D E F G

PHAP Instructions: Please answer each question based on your listening experiences recently.

Please circle the answer that comes closest to your everyday experience. Notice that each choice includes a percentage. You can use this to help you decide on your answer. For example if the statement is true about 75% of the time, circle C for that item. If you have not experienced a particular situation, imagine how you would respond in a similar situation.

A = Always (99%)	D = Half-the-Time (50%)	G = Never (1%)
B = Almost Always (87%)	E = Occasionally (25%)	
C = Generally (75%)	F = Seldom (12%)	

63. Traffic noises are too loud.

A B C D E F G

64. The sound of glass breaking is uncomfortably loud.

A B C D E F G

65. I can communicate with others when we are in a crowd.

A B C D E F G

66. I can understand speech when I am talking to a bank teller, and I am one of a few customers at the bank.

A B C D E F G

PHAB Score Sheet

Familiar Talkers (FT) 1*, 20*, 32*, 48*, 52*, 61*, 66*

Ease of Communication (EC) 12, 17, 25, 29, 33, 34, 38

Reverberation (RV) 2, 14, 22*, 26, 31, 39*, 45*, 53*, 58

Reduced Cues (RC) 4, 6*, 35, 37*, 46*, 47, 49*, 54, 59

Background Noise (BN) 5, 7*, 8*, 10, 11*, 16, 18, 23, 27, 28, 30, 42*, 44, 56, 62*, 65*

Aversiveness of Sounds (AV) 9, 13, 15, 19, 21, 24, 41, 43, 55,

57, 63

Distortion of Sounds (DS) 3, 36, 40, 50, 51, 60

SUBJECT #: ____________ TEST DATE: ________

EXAMINER'S INITIALS: ________

Hearing Handicap Inventory for the Elderly (HHIE)

(UNAIDED)

The purpose of this scale is to identify the problems your hearing loss may be causing you. Check 'Yes', 'Sometimes', or 'No' for each question. Do not skip any questions.

S-1. Does a hearing problem cause you to use the phone less often than you would like?	☐Yes ☐Sometimes ☐No
E-2. Does a hearing problem cause you to feel embarrassed when meeting new people?	☐Yes ☐Sometimes ☐No
S-3. Does a hearing problem cause you to avoid groups of people?	☐Yes ☐Sometimes ☐No
E-4. Does a hearing problem make you irritable?	☐Yes ☐Sometimes ☐No
E-5. Does a hearing problem cause you to feel frustrated when talking to members of your family?	☐Yes ☐Sometimes ☐No
S-6. Does a hearing problem cause you difficulty when attending a party?	☐Yes ☐Sometimes ☐No
E-7. Does a hearing problem cause you to feel "stupid" or "dumb"	☐Yes ☐Sometimes ☐No
S-8. Do you have difficulty hearing when someone speaks in a whisper?	☐Yes ☐Sometimes ☐No

E-9. Do you feel handicapped by a hearing problem?	☐Yes ☐Sometimes ☐No
S-10. Does a hearing problem cause you difficulty when visiting friends, relatives or neighbors?	☐Yes ☐Sometimes ☐No
S-11. Does a hearing problem cause you to attend religious services less often than you would like?	☐Yes ☐Sometimes ☐No
E-12. Does a hearing problem cause you to be nervous?	☐Yes ☐Sometimes ☐No
S-13. Does a hearing problem cause you to visit friends, relatives, or neighbors less often than you would like?	☐Yes ☐Sometimes ☐No
E-14. Does a hearing problem cause you to have arguments with family members?	☐Yes ☐Sometimes ☐No
S-15. Does a hearing problem cause you difficulty when listening to TV or radio?	☐Yes ☐Sometimes ☐No
S-16. Does a hearing problem cause you to go shopping less often than you would like?	☐Yes ☐Sometimes ☐No
E-17. Does any problem or difficulty with your hearing upset you at all?	☐Yes ☐Sometimes ☐No
E-18. Does a hearing problem cause you to want to be by yourself?	☐Yes ☐Sometimes ☐No
S-19. Does a hearing problem cause you to talk to family members less often than you would like?	☐Yes ☐Sometimes ☐No

E-20. Do you feel that any difficulty with your hearing limits or hampers your personal or social life?	☐Yes ☐Sometimes ☐No
S-21. Does a hearing problem cause you difficulty when in a restaurant with relatives or friends?	☐Yes ☐Sometimes ☐No
E-22. Does a hearing problem cause you to feel depressed?	☐Yes ☐Sometimes ☐No
S-23. Does a hearing problem cause you to listen to TV or radio less often than you would like?	☐Yes ☐Sometimes ☐No
E-24. Does a hearing problem cause you to feel uncomfortable when talking to friends?	☐Yes ☐Sometimes ☐No
E-25. Does a hearing problem cause you to feel left out when you are with a group of people?	☐Yes ☐Sometimes ☐No

ABCD Study- PHAP AIDED- Session 3

SUBJECT #: _______________ TEST DATE: __________
EXAMINER'S INITIALS: _________

PHAP Instructions: Please answer each question based on your listening experiences WITH YOUR HEARING AIDS OVER THE PAST SEVERAL WEEKS.

Please circle the answer that comes closest to your everyday experience. Notice that each choice includes a percentage. You can use this to help you decide on your answer. For example if the statement is true about 75% of the time, circle C for that item. If you have not experienced a particular situation, imagine how you would respond in a similar situation.

A = Always (99%)	D = Half-the-time (50%)	G = Never (1%)
B = Almost always (87%)	E = Occasionally (25%)	
C = Generally (75%)	F = Seldom (12%)	

1. I can understand others in a small group situation if there is no noise.

A B C D E F G

2. When I am listening to a speaker who is talking to a large group, and I am seated toward the rear of the room, I must make an effort to listen.

A B C D E F G

PHAP Instructions: Please answer each question based on your listening experiences recently WITH YOUR HEARING AIDS OVER THE PAST SEVERAL WEEKS.

Please circle the answer that comes closest to your everyday experience. Notice that each choice includes a percentage. You can use this to help you decide on your answer. For example if the statement is true about 75% of the time, circle C for that item. If you have not experienced a particular situation, imagine how you would respond in a similar situation.

A = Always (99%)	D = Half-the-Time (50%)	G = Never (1%)
B = Almost Always (87%)	E = Occasionally (25%)	
C = Generally (75%)	F = Seldom (12%)	

3. Women’s voices sound “shrill.”

A B C D E F G

4. I find that most people speak too softly.

A B C D E F G

5. I have trouble comprehending speech when I am in a busy department store talking with the clerk.

A B C D E F G

6. I can understand my family when they speak softly to me.

A B C D E F G

PHAP Instructions: Please answer each question based on your listening experiences recently WITH YOUR HEARING AIDS OVER THE PAST SEVERAL WEEKS.

Please circle the answer that comes closest to your everyday experience. Notice that each choice includes a percentage. You can use this to help you decide on your answer. For example if the statement is true about 75% of the time, circle C for that item. If you have not experienced a particular situation, imagine how you would respond in a similar situation.

A = Always (99%)	D = Half-the-Time (50%)	G = Never (1%)
B = Almost Always (87%)	E = Occasionally (25%)	
C = Generally (75%)	F = Seldom (12%)	

7. I can understand a speaker in a small group, even when those around us are speaking softly to each other.

A B C D E F G

8. I can understand conversations even when several people are talking.

A B C D E F G

9. When the telephone rings, the sound startles me.

A B C D E F G

10. I have to ask people to repeat themselves when there is background noise.

A B C D E F G

PHAP Instructions: Please answer each question based on your listening experiences recently WITH YOUR HEARING AIDS OVER THE PAST SEVERAL WEEKS.

Please circle the answer that comes closest to your everyday experience. Notice that each choice includes a percentage. You can use this to help you decide on your answer. For example if the statement is true about 75% of the time, circle C for that item. If you have not experienced a particular situation, imagine how you would respond in a similar situation.

A = Always (99%)	D = Half-the-Time (50%)	G = Never (1%)
B = Almost Always (87%)	E = Occasionally (25%)	
C = Generally (75%)	F = Seldom (12%)	

11. When I am in a crowded grocery store, talking with the cashier, I can follow the conversation.

A B C D E F G

12. When I am having a conversation, and people are talking quietly nearby, I have to strain to understand the speaker.

A B C D E F G

13. If a car horn sounds, it makes me jump.

A B C D E F G

14. When I am talking to a group and someone from the back of the room asks a question, I have to ask someone up front to repeat the question.

A B C D E F G

PHAP Instructions: Please answer each question based on your listening experiences recently WITH YOUR HEARING AIDS OVER THE PAST SEVERAL WEEKS.

Please circle the answer that comes closest to your everyday experience. Notice that each choice includes a percentage. You can use this to help you decide on your answer. For example if the statement is true about 75% of the time, circle C for that item. If you have not experienced a particular situation, imagine how you would respond in a similar situation.

A = Always (99%)	D = Half-the-Time (50%)	G = Never (1%)
B = Almost Always (87%)	E = Occasionally (25%)	
C = Generally (75%)	F = Seldom (12%)	

15. The sounds of construction work are uncomfortably loud.

A B C D E F G

16. When I am in a crowded reception room waiting to be called, I miss hearing my name.

A B C D E F G

17. When I am having a quiet conversation with a companion, I have difficulty understanding.

A B C D E F G

18. When I am listening to the news on the car radio, and family members are talking, I have trouble hearing the news.

A B C D E F G

PHAP Instructions: Please answer each question based on your listening experiences recently WITH YOUR HEARING AIDS OVER THE PAST SEVERAL WEEKS.

Please circle the answer that comes closest to your everyday experience. Notice that each choice includes a percentage. You can use this to help you decide on your answer. For example if the statement is true about 75% of the time, circle C for that item. If you have not experienced a particular situation, imagine how you would respond in a similar situation.

A = Always (99%)	D = Half-the-Time (50%)	G = Never (1%)
B = Almost Always (87%)	E = Occasionally (25%)	
C = Generally (75%)	F = Seldom (12%)	

19. The sound of screeching tires is uncomfortably loud.

A B C D E F G

20. I can understand conversation when I am walking with a friend through a quiet park.

A B C D E F G

21. The sound of a fire engine siren close by is so loud that I need to cover my ears.

A B C D E F G

22. When I am in conversation with someone across a large empty room (such as an auditorium), I understand the words.

A B C D E F G

PHAP Instructions: Please answer each question based on your listening experiences recently WITH YOUR HEARING AIDS OVER THE PAST SEVERAL WEEKS.

Please circle the answer that comes closest to your everyday experience. Notice that each choice includes a percentage. You can use this to help you decide on your answer. For example if the statement is true about 75% of the time, circle C for that item. If you have not experienced a particular situation, imagine how you would respond in a similar situation.

A = Always (99%)	D = Half-the-Time (50%)	G = Never (1%)
B = Almost Always (87%)	E = Occasionally (25%)	
C = Generally (75%)	F = Seldom (12%)	

23. When I am on a busy street, asking someone for directions, I have to ask him to repeat them before I really understand.

A B C D E F G

24. The sound of running water, such as a toilet or shower, is uncomfortably loud.

A B C D E F G

25. When a speaker is addressing a small group, and everyone is listening quietly, I have to strain to understand.

A B C D E F G

26. I have trouble understanding dialogue in a movie or at the theater.

A B C D E F G

PHAP Instructions: Please answer each question based on your listening experiences recently WITH YOUR HEARING AIDS OVER THE PAST SEVERAL WEEKS.

Please circle the answer that comes closest to your everyday experience. Notice that each choice includes a percentage. You can use this to help you decide on your answer. For example if the statement is true about 75% of the time, circle C for that item. If you have not experienced a particular situation, imagine how you would respond in a similar situation.

A = Always (99%)	D = Half-the-Time (50%)	G = Never (1%)
B = Almost Always (87%)	E = Occasionally (25%)	
C = Generally (75%)	F = Seldom (12%)	

27. When I am in a crowd with a friend who doesn’t want others to overhear our conversation, I have trouble hearing as well.

A B C D E F G

28. When I am at the dinner table with several people, and am trying to have a conversation with one person, understanding speech is difficult.

A B C D E F G

29. When I'm in a quiet conversation with my doctor in an examination room, it is hard to follow the conversation.

A B C D E F G

30. I have trouble understanding others when an air conditioner or fan is on.

A B C D E F G

PHAP Instructions: Please answer each question based on your listening experiences recently WITH YOUR HEARING AIDS OVER THE PAST SEVERAL WEEKS.

Please circle the answer that comes closest to your everyday experience. Notice that each choice includes a percentage. You can use this to help you decide on your answer. For example if the statement is true about 75% of the time, circle C for that item. If you have not experienced a particular situation, imagine how you would respond in a similar situation.

A = Always (99%) D = Half-the-Time (50%) G = Never (1%)

B = Almost Always (87%) E = Occasionally (25%)

C = Generally (75%) F = Seldom (12%)

31. I miss a lot of information when I'm listening to a lecture.

A B C D E F G

32. I can understand my family when they talk to me a normal voice.

A B C D E F G

33. I have to ask people to repeat themselves in one-on-one conversation in a quiet room.

A B C D E F G

34. I have difficulty hearing conversation when I'm with one other person at home.

A B C D E F G

PHAP Instructions: Please answer each question based on your listening experiences recently WITH YOUR HEARING AIDS OVER THE PAST SEVERAL WEEKS.

Please circle the answer that comes closest to your everyday experience. Notice that each choice includes a percentage. You can use this to help you decide on your answer. For example if the statement is true about 75% of the time, circle C for that item. If you have not experienced a particular situation, imagine how you would respond in a similar situation.

A = Always (99%)	D = Half-the-Time (50%)	G = Never (1%)
B = Almost Always (87%)	E = Occasionally (25%)	
C = Generally (75%)	F = Seldom (12%)	

35. When I am riding in the back seat of a car, and the driver talks to me from the front, I have to strain to understand.

A B C D E F G

36. Music sounds distorted to me.

A B C D E F G

37. When I'm talking with the teller at the drive-in window of my bank, I understand the speech coming from the loudspeaker.

A B C D E F G

38. When I am in a small office, interviewing or answering questions, I have difficulty following the conversation.

A B C D E F G

PHAP Instructions: Please answer each question based on your listening experiences recently WITH YOUR HEARING AIDS OVER THE PAST SEVERAL WEEKS.

Please circle the answer that comes closest to your everyday experience. Notice that each choice includes a percentage. You can use this to help you decide on your answer. For example if the statement is true about 75% of the time, circle C for that item. If you have not experienced a particular situation, imagine how you would respond in a similar situation.

A = Always (99%) D = Half-the-Time (50%) G = Never (1%)

B = Almost Always (87%) E = Occasionally (25%)

C = Generally (75%) F = Seldom (12%)

39. When a lecturer is giving instructions, I can easily follow along.

A B C D E F G

40. Everyday sounds are too soft for me to hear clearly.

A B C D E F G

41. I avoid using certain appliances (blender, vacuum cleaner, etc.) because their loudness is uncomfortable.

A B C D E F G

42. When I am in a busy restaurant and the waitress is taking my order, I can comprehend her questions.

A B C D E F G

PHAP Instructions: Please answer each question based on your listening experiences recently WITH YOUR HEARING AIDS OVER THE PAST SEVERAL WEEKS.

Please circle the answer that comes closest to your everyday experience. Notice that each choice includes a percentage. You can use this to help you decide on your answer. For example if the statement is true about 75% of the time, circle C for that item. If you have not experienced a particular situation, imagine how you would respond in a similar situation.

A = Always (99%) D = Half-the-Time (50%) G = Never (1%)

B = Almost Always (87%) E = Occasionally (25%)

C = Generally (75%) F = Seldom (12%)

43. I avoid crowds because the noise is uncomfortably loud.

A B C D E F G

44. When I am at a large, noisy party, conversation is very confusing.

A B C D E F G

45. When I am in a theater watching a movie or play, and the people around me are whispering and rustling paper wrappers, I can still make out the dialogue.

A B C D E F G

46. When I am in a quiet restaurant, I can understand soft conversation.

A B C D E F G

PHAP Instructions: Please answer each question based on your listening experiences recently WITH YOUR HEARING AIDS OVER THE PAST SEVERAL WEEKS.

Please circle the answer that comes closest to your everyday experience. Notice that each choice includes a percentage. You can use this to help you decide on your answer. For example if the statement is true about 75% of the time, circle C for that item. If you have not experienced a particular situation, imagine how you would respond in a similar situation.

A = Always (99%) D = Half-the-Time (50%) G = Never (1%)

B = Almost Always (87%) E = Occasionally (25%)

C = Generally (75%)   F = Seldom (12%)

47. I can’t understand the TV news when the volume is set by a normal-hearing person.

A B C D E F G

48. I can understand conversation during a quiet dinner with my family.

A B C D E F G

49. When I am listening to the news on my car radio, and the car windows are closed, I understand the words.

A B C D E F G

50. The sound quality of music isn’t very good.

A B C D E F G

PHAP Instructions: Please answer each question based on your listening experiences recently WITH YOUR HEARING AIDS OVER THE PAST SEVERAL WEEKS.

Please circle the answer that comes closest to your everyday experience. Notice that each choice includes a percentage. You can use this to help you decide on your answer. For example if the statement is true about 75% of the time, circle C for that item. If you have not experienced a particular situation, imagine how you would respond in a similar situation.

A = Always (99%)   D = Half-the-Time (50%)   G = Never (1%)

B = Almost Always (87%) E = Occasionally (25%)

C = Generally (75%) F = Seldom (12%)

51. The ring of a telephone sounds "tinny."

A B C D E F G

52. I understand the newscaster when I am watching TV news at home alone.

A B C D E F G

53. I can follow the words of a sermon when listening to a religious service.

A B C D E F G

54. When I am at home, talking with someone who is in another room, following the conversation is difficult.

A B C D E F G

PHAP Instructions: Please answer each question based on your listening experiences recently WITH YOUR HEARING AIDS OVER THE PAST SEVERAL WEEKS.

Please circle the answer that comes closest to your everyday experience. Notice that each choice includes a percentage. You can use this to help you decide on your answer. For example if the statement is true about 75% of the time, circle C for that item. If you have not experienced a particular situation, imagine how you would respond in a similar situation.

A = Always (99%) D = Half-the-Time (50%) G = Never (1%)

B = Almost Always (87%) E = Occasionally (25%)

C = Generally (75%) F = Seldom (12%)

55. Unexpected sounds like a smoke detector or alarm bell are uncomfortable.

A B C D E F G

56. When I'm talking with a friend outdoors on a windy day, I miss a lot of the conversation.

A B C D E F G

57. Everyday sounds that don't bother others are too loud for me.

A B C D E F G

58. It's hard for me to understand what is being said at lectures or church services.

A B C D E F G

PHAP Instructions: Please answer each question based on your listening experiences recently WITH YOUR HEARING AIDS OVER THE PAST SEVERAL WEEKS.

Please circle the answer that comes closest to your everyday experience. Notice that each choice includes a percentage. You can use this to help you decide on your answer. For example if the statement is true about 75% of the time, circle C for that item. If you have not

experienced a particular situation, imagine how you would respond in a similar situation.

A = Always (99%)	D = Half-the-Time (50%)	G = Never (1%)
B = Almost Always (87%)	E = Occasionally (25%)	
C = Generally (75%)	F = Seldom (12%)	

59. When I am in a room with the door closed and I want to over-hear a conversation going on outside the door, I have to strain to listen.

A B C D E F G

60. People's voices sound unnatural.

A B C D E F G

61. When I am in face-to-face conversation with one member of my family, I can easily follow along.

A B C D E F G

62. When I am in a meeting with several other people, I can comprehend speech.

A B C D E F G

PHAP Instructions: Please answer each question based on your listening experiences recently WITH YOUR HEARING AIDS OVER THE PAST SEVERAL WEEKS.

Please circle the answer that comes closest to your everyday experience. Notice that each choice includes a percentage. You can use this to help you decide on your answer. For example if the statement is

true about 75% of the time, circle C for that item. If you have not experienced a particular situation, imagine how you would respond in a similar situation.

A = Always (99%)	D = Half-the-Time (50%)	G = Never (1%)
B = Almost Always (87%)	E = Occasionally (25%)	
C = Generally (75%)	F = Seldom (12%)	

63. Traffic noises are too loud.

A B C D E F G

64. The sound of glass breaking is uncomfortably loud.

A B C D E F G

65. I can communicate with others when we are in a crowd.

A B C D E F G

66. I can understand speech when I am talking to a bank teller, and I am one of a few customers at the bank.

A B C D E F G

PHAB Score Sheet

Familiar Talkers (FT) 1*, 20*, 32*, 48*, 52*, 61*, 66*

Ease of Communication (EC) 12, 17, 25, 29, 33, 34, 38

Reverberation (RV) 2, 14, 22*, 26, 31, 39*, 45*, 53*, 58

Reduced Cues (RC) 4, 6*, 35, 37*, 46*, 47, 49*, 54, 59

Background Noise (BN) 5, 7*, 8*, 10, 11*, 16, 18, 23, 27, 28, 30, 42*, 44, 56, 62*, 65*

Aversiveness of Sounds (AV) 9, 13, 15, 19, 21, 24, 41, 43, 55,

57, 63

Distortion of Sounds (DS) 3, 36, 40, 50, 51, 60

SUBJECT #: ____________ TEST DATE: ________

EXAMINER'S INITIALS: ________

Hearing Handicap Inventory for the Elderly (HHIE)
(AIDED)

The purpose of this scale is to identify the problems your hearing loss may be causing you. Check 'Yes', 'Sometimes', or 'No' for each question. Do not skip any questions. Answer these questions based on your listening experiences WITH YOUR HEARING AIDS OVER THE PAST SEVERAL WEEKS.

S-1. Does a hearing problem cause you to use the phone less often than you would like?	☐Yes ☐Sometimes ☐No
E-2. Does a hearing problem cause you to feel embarrassed when meeting new people?	☐Yes ☐Sometimes ☐No
S-3. Does a hearing problem cause you to avoid groups of people?	☐Yes ☐Sometimes ☐No
E-4. Does a hearing problem make you irritable?	☐Yes ☐Sometimes ☐No
E-5. Does a hearing problem cause you to feel frustrated when talking to members of your family?	☐Yes ☐Sometimes ☐No
S-6. Does a hearing problem cause you difficulty when attending a party?	☐Yes ☐Sometimes ☐No
E-7. Does a hearing problem cause you to feel "stupid" or "dumb"	☐Yes ☐Sometimes ☐No
S-8. Do you have difficulty hearing when someone speaks in a whisper?	☐Yes ☐Sometimes ☐No

E-9. Do you feel handicapped by a hearing problem?	☐Yes ☐Sometimes ☐No
S-10. Does a hearing problem cause you difficulty when visiting friends, relatives or neighbors?	☐Yes ☐Sometimes ☐No
S-11. Does a hearing problem cause you to attend religious services less often than you would like?	☐Yes ☐Sometimes ☐No
E-12. Does a hearing problem cause you to be nervous?	☐Yes ☐Sometimes ☐No
S-13. Does a hearing problem cause you to visit friends, relatives, or neighbors less often than you would like?	☐Yes ☐Sometimes ☐No
E-14. Does a hearing problem cause you to have arguments with family members?	☐Yes ☐Sometimes ☐No
S-15. Does a hearing problem cause you difficulty when listening to TV or radio?	☐Yes ☐Sometimes ☐No
S-16. Does a hearing problem cause you to go shopping less often than you would like?	☐Yes ☐Sometimes ☐No
E-17. Does any problem or difficulty with your hearing upset you at all?	☐Yes ☐Sometimes ☐No
E-18. Does a hearing problem cause you to want to be by yourself?	☐Yes ☐Sometimes ☐No
S-19. Does a hearing problem cause you to talk to family members less often than you would like?	☐Yes ☐Sometimes ☐No

E-20. Do you feel that any difficulty with your hearing limits or hampers your personal or social life?	☐Yes ☐Sometimes ☐No
S-21. Does a hearing problem cause you difficulty when in a restaurant with relatives or friends?	☐Yes ☐Sometimes ☐No
E-22. Does a hearing problem cause you to feel depressed?	☐Yes ☐Sometimes ☐No
S-23. Does a hearing problem cause you to listen to TV or radio less often than you would like?	☐Yes ☐Sometimes ☐No
E-24. Does a hearing problem cause you to feel uncomfortable when talking to friends?	☐Yes ☐Sometimes ☐No
E-25. Does a hearing problem cause you to feel left out when you are with a group of people?	☐Yes ☐Sometimes ☐No

HEARING AID SATISFACTION SURVEY

1. INSTRUCTIONS: Listed below are some hearing aid features. For each feature, check a box to show **how satisfied** you are with that feature. By the term Neutral we mean neither satisfied nor dissatisfied. **PLEASE CHECK ONLY ONE BOX FOR EACH FEATURE.**

**Hearing Aid Feature**		**Very Satisfied**	**Satisfied**	**Neutral**	**Dissatisfied**	**Very Dissatisfied**
A.	Overall fit/comfort	❐	❐	❐	❐	❐
B.	Visibility to others	❐	❐	❐	❐	❐
C.	Hearing aid size	❐	❐	❐	❐	❐
D.	Ease of changing battery	❐	❐	❐	❐	❐
E.	Battery life	❐	❐	❐	❐	❐
F.	Clearness of tone & sound	❐	❐	❐	❐	❐
G.	Whistling/feedback/ buzzing	❐	❐	❐	❐	❐
H.	Ease of adjusting volume	❐	❐	❐	❐	❐
I.	Reliability	❐	❐	❐	❐	❐
J.	Appearance of hearing aid	❐	❐	❐	❐	❐
K.	Improves your hearing	❐	❐	❐	❐	❐
L.	Use in noisy situations	❐	❐	❐	❐	❐
M.	Ongoing expense of hearing aid	❐	❐	❐	❐	❐
N.	Value (performance vs. money spent)	❐	❐	❐	❐	❐

⇨

Hearing Aid Feature	Very Satisfied	Satisfied	Neutral	Dissatisfied	Very Dissatisfied
O. Natural sounding	❒	❒	❒	❒	❒
P. Ability to tell location of sounds	❒	❒	❒	❒	❒
Q. Frequency of cleaning Required	❒	❒	❒	❒	❒
R. Warranty on the hearing aids	❒	❒	❒	❒	❒
S. Packaging of the aids	❒	❒	❒	❒	❒

2. INSTRUCTIONS: Listed below are some features about the **service you received from the personnel in the IU Clinical Trials Group.** For each service factor check a box to show **how satisfied** you are with the **service factors. PLEASE CHECK ONLY ONE BOX FOR EACH FEATURE.**

Service Factor	Very Satisfied	Satisfied	Neutral	Dissatisfied	Very Dissatisfied
A. Professionalism of personnel	❒	❒	❒	❒	❒
B. Friendliness of personnel	❒	❒	❒	❒	❒
C. Personnel’s knowledge of hearing aids	❒	❒	❒	❒	❒
D. Patience of personnel	❒	❒	❒	❒	❒
E. Explanation of purpose of hearing test	❒	❒	❒	❒	❒
F. Explanation of results of hearing test	❒	❒	❒	❒	❒
G. Explanation of how to use hearing aids	❒	❒	❒	❒	❒

H.	Explanation of how to care for your hearing aids	❒	❒	❒	❒	❒
I.	Explanation of what to expect from your hearing aids	❒	❒	❒	❒	❒
J.	Amount of time personnel spent with you	❒	❒	❒	❒	❒
K.	Cleanliness/appearance of personnel's office	❒	❒	❒	❒	❒
L.	Quality of service after purchase	❒	❒	❒	❒	❒
M.	Promptness of service	❒	❒	❒	❒	❒

MODIFIED PRACTICAL HEARING SKILLS TEST-REVISED (PHAST-R-MOD)

SUBJECT # _____________ E TEST DATE _____________

Instructions: Place the following items in front of the patient: A telephone, Different size batteries (10, 312, 13, etc.), Magnetic tool for battery removal, Cleaning tools: brush, cloth, wax loop.

Complete the entire PHAST-R-MOD (Reinstruct on items AFTER the PHAST-R-MOD is completed). Reinstruct patients on the tasks that he/she received a score of 1 or 0.

Scoring:

2 Performs the task without any problems.

1 Performs the task using 'deviant' means (e.g., takes aid out to adjust VC), needs some re-instruction.

0 Cannot perform task.

1. Ask the patient, "Please take out your hearing aid."

a. Can he/she grasp the aid?	Right:	O 0	O 1	O 2	O N/A
	Left:	O 0	O 1	O 2	O N/A
b. Can he/she remove the aid properly?	Right:	O 0	O 1	O 2	O N/A
	Left:	O 0	O 1	O 2	O N/A

2. Ask the patient, "Open up the battery door."

a. Can he/she locate the door on the first try?	O 0	O 1	O 2	O N/A
b. Can he/she open the door without difficulty?	O 0	O 1	O 2	O N/A

3. Ask the patient, "Please show me how you change your hearing aid battery."

a. Can he/she remove old battery?	O 0	O 1	O 2	O N/A
b. Did he/she choose correct battery size?	O 0	O 1	O 2	O N/A
c. Can he/she remove battery tab?	O 0	O 1	O 2	O N/A
d. Can he/she correctly place new battery in battery compartment?	O 0	O 1	O 2	O N/A

4. Ask the patient, "Please show me how you clean your hearing aid."

a. Can he/she clean the sound bore?		O 0	O 1	O 2	O N/A
b. Can he/she brush the microphone port?		O 0	O 1	O 2	O N/A
c. Can he/she clean the open fit dome?		O 0	O 1	O 2	O N/A

5. Ask the patient, "Please show me how you might change the dome."

a. Can he/she properly change the dome?		O 0	O 1	O 2	O N/A

6. Ask the patient, "Please show me how you would clean the tube if necessary."

a. Can he/she remove the tube and clean it?		O 0	O 1	O 2	O N/A

7. Ask the patient, "Please put your hearing aid back in your ear."

a. Can he/she grasp the aid?	Right:	O 0	O 1	O 2	O N/A
	Left:	O 0	O 1	O 2	O N/A
b. Can he/she place the aid properly in the ear?					
	Right:	O 0	O 1	O 2	O N/A
	Left:	O 0	O 1	O 2	O N/A

8. Ask the patient, "Please adjust the volume of your hearing aid."

a. Can he/she correctly manipulate the program button to change the volume?

	Right:	O 0	O 1	O 2	O N/A
	Left:	O 0	O 1	O 2	O N/A

9. Ask the patient, "Show me how you use the telephone with your hearing aid." (hand phone to subject).

a. Can he/she correctly place the phone in relation to the aid?

O 0 O 1 O 2 O N/A

10. Ask the patient, "Did you consult with anyone outside of the study about these skills?

O Yes O No

If YES, Please tell me more about that: ____

Unscheduled Visit Checklist

Subject #: ___________ **Status:** ________________ **DATE:** _______________

Serial #: _______________ **Ear:** ____ **EXAMINER'S INITIALS:** _______

Primary Complaint: ____

**Cerumen (ear wax) or debris:**	**YES**	**NO**	**ACTION TAKEN**
Cerumen/debris on dome?			
Cerumen/debris in tubing?			
Cerumen/debris on microphone port(s)?			

**Battery:**	**YES**	**NO**	**ACTION TAKEN**
No battery?			
Battery inserted incorrectly?			
Battery dead?			

**Hearing aid parts:**	**YES**	**NO**	**ACTION TAKEN**
Tubing missing?			
Dome missing?			
Tubing incorrectly attached?			
Dome incorrectly attached?			
Tubing broken?			
Dome broken?			
HA casing broken?			

☐ **Problem resolved**: remind subject of their Session 3 appointment date and time and escort out.

☐ **Problem not resolved**: Tell subject, "I need to run some checks on the hearing aid, I will be right back," then consult Session 1 audiologist.

Additional Notes:

____

____

____

____

____

____

____

____

EVALUATING HEARING AID SERVICE-DELIVERY MODELS (ABCD)

NEW HEARING AID CHECK-IN PROTOCOL
11/2/12

1. If you are the first person logging onto the computer for the day, you will need to log onto the server.
    a. Log onto the computer as CTG and use the password from the FYI document in ABCD folder on server
    b. Click on the CTG server shortcut on the desktop.
    c. You will be required to log onto the server
        i. Username: ADS\your username
        ii. Password: your password
2. If you are turning the Verifit on for the first time today, you will be prompted to calibrate the test box prior to checking in a hearing aid. See Calibration Instructions.
3. Record date, serial number and color in check-in chart.
4. Remove hearing aid(s) from hard case and set aside (A-H may already be done).
    a. File the manufacturer's spec sheets under CD or AB in black cabinet for Chris
    b. Mark out "GN RESOUND", address, and GNR312ZM (upper right corner) on back of battery case and put in black cabinet.
    c. Put unmarked zippered case (cleaning cloth and brush inside) on black cabinet shelf labeled "CASES".
    d. Put red/blue indicators in corresponding plastic bag in black cabinet shelf.
    e. Put foam in red bin in black cabinet.
    f. Cardboard casing can be broken down and recycled.
    g. Put hard "Resound" labeled case and user guide (with paper user guide inside) into cardboard box behind soundbooth to be sent back.
    h. Dispose of any earhooks, instructions or other materials.
5. Put 2A slim tube on hearing aid for ANSI. Compare to ANSI S3.22-2003 specs for Resound Alera open BTE (located on HA check-in chart).
6. Connect hearing aids to Aventa software.
    a. For all hearing aids- select: **"ANSI, Check-in"**
    b. Click "Aventa" on upper right hand corner to load fitting software
    c. Hook up hearing aid to coupler and place in test box with microphone 1-2mm from reference microphone (see next page for mic and hearing aid placement example). Close battery door **with battery pill** inside.
    d. Click "Connect" with Airlink.
    e. When logging on to a patient's name that has already had a hearing aid programmed to it, it will give you an error box that gives you an option to "Replace Instruments". Click this option.
    f. Click "unassign" under previously connected instruments, then click "find" to connect to your new hearing aid (you may or may not have to open/close battery door again).
    g. Once the hearing aid has been detected you will have to assign it to the **left** ear before you can proceed then press "done".
    h. If prompted, select "Keep current session's fitting data".
    i. If prompted regarding which audiogram to choose, just "X" (close) out of the box.
    j. Before running ANSI, click on "Beeps and Volume Control" and verify that ALL boxes are checked (this is a default setting so it should be every time).

7. Run ANSI

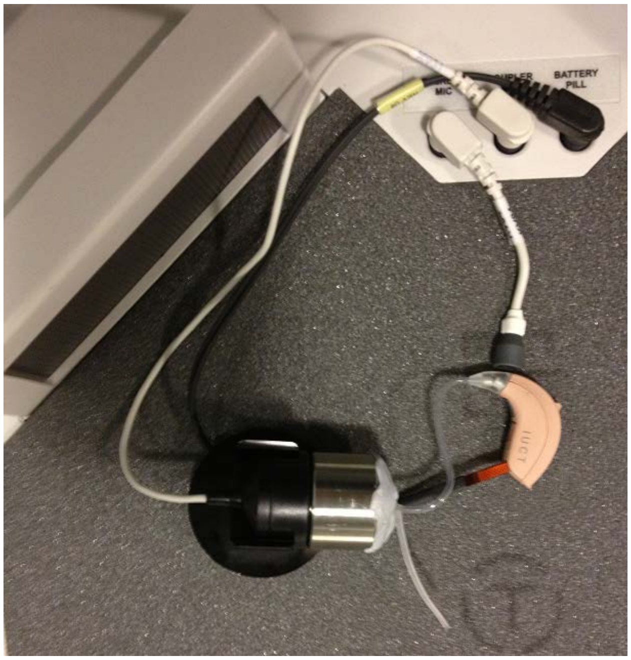

a. Hearing aid placed over target, reference mic next to hearing aid microphone, coupler screwed into coupler microphone (see picture above). This placement will be used for ANSI and Directional measurements
b. Close and latch test box
c. On the Verifit push "Tests"→"Test box measures"→"AGC"→ "Start Test"
d. A box will pop up prompting you to turn the hearing aid "full on"
e. Hearing aid should already be set to "Full On", but ensure all of the following are set correctly in Aventa:
    i. In Aventa software, click "view"→"coupler" → "insertion gain"
    ii. Click on "P4" and check that it is set to REM test (turns off all program features).
    iii. Under the sidebar "Tools" click on "Physical Properties"
        1. Setting should be set to "None" and press "OK"
        2. If it asks you if you would like to calibrate, don't, and press "close".
    iv. "Enable Safe Fitting" (under "Fitting") should not be checked
    v. Tools sidebar adjustments:
        1. Environmental Optimizer values should be re-set to zero for all environments
        2. Click on "Advanced Features"
            a. Directionality: omni
            b. All other settings (DFS, Expansion, Noisetracker II, and Windguard) should be **OFF**
    vi. Click on "Gain Adjustment".
        1. Values in frequency gain response should match FOG values:

**FOG**	**.25**	**.5**	**.75**	**1**	**1.5**	**2**	**3**	**4**	**6**
**G(50)**	48	48	48	48	46	43	40	38	38
**G(80)**	32	32	32	32	31	29	26	24	24

        2. Values in MPO settings should match these values:

**MPO**	**.25**	**.5**	**.75**	**1**	**1.5**	**2**	**3**	**4**	**6**
**Max**	114	116	121	120	119	120	123	118	107

vii. Click "Continue" on Verifit when FOG and MPO values match to begin ANSI measurements.

f. After the first curve is complete, a box will pop up instructing you to "Set VC to RTS"

i. Select program 3 in Aventa. The values in frequency gain response should match RTG values:

RTG	.25	.5	.75	1	1.5	2	3	4	6
G(50)	44	44	44	44	42	39	36	34	34
G(80)	28	28	28	28	27	25	22	20	20

ii. Press "Continue" on Verifit when values match.

g. After the second curve is complete, a box will pop up instructing you to "Set the AGC function of the hearing instrument to have maximum effect as specified by the manufacturer. Press "continue" on the Verifit.

h. Compare to manufacturer's specifications and to the ANSI tolerance requirements on HA Check-in Chart

i. ignore "Attack and Release" measurements

ii. ignore "EIN" measurements

i. Record if HA meets ANSI specs within tolerance requirements (Y/N) on check-in sheet.

i. If HA **does not** meet specs, run second time. If still doesn't meet specs send hearing aid back to manufacturer for repair/replacement.

ii. If HA **does** meet specs, switch display from "graph" to "table" and print measurements on USB (can rename file now as ANSIsn).

8. Directional microphone verification

a. Return to Program 1- Basic + Softswitching settings in Aventa

b. Check that the following settings are correct under "Advanced Features"

i. In this program, Directionality should be FIXED

ii. Directional Mix: Very Low

iii. DFS Ultra: Moderate

iv. Expansion: Off

v. Noise Tracker II: Per Environment

vi. Wind Guard: Off

c. Leave the hearing aid set up as it was for ANSI: top microphone 1-2 mm from reference microphone & bottom microphone facing right speaker.

d. On the Verifit click "Tests"→"test box measures"→"Directional"

i. Being in graph view initially will help you better visualize when directional microphone has kicked in.

e. Adjust level to 65 and SNR to 3

f. Click "continue" once lines have stabilized and verify that the noise coming from R speaker (line marked with "R") is lower than speech coming from L speaker (line marked with "L").

g. If correctly positioned directional microphone is displayed, check column labeled "Directional mic verified?" on check-in sheet.

h. Change format to "table" on Verifit and print to USB (can rename file now as Dsn)

i. Close out of Aventa without saving.

j. AB Hearing Aids: Transfer USB to Laptop to rename files from the Verifit (you could have also renamed the files when printing on the Verifit). Use the file naming sheet to name each file appropriately. Put manufacturer's specs in AB Group folder in cabinet. Then continue on to **Step 10**.

k. CD Hearing Aids: Continue on to **Step 9**.

9. CD GROUP: Simulated Speechmapping

a. Label hearing aids X, Y, or Z respectively.

b. Click on X, Y, or Z check-in patient on Aventa software.

c. Click "Aventa" on upper right hand corner to load fitting software.

d. Hook up hearing aid to coupler and place in test box with microphone 1-2mm from reference microphone (see picture below for how to orient the hearing aid). Close battery door **with battery** inside.

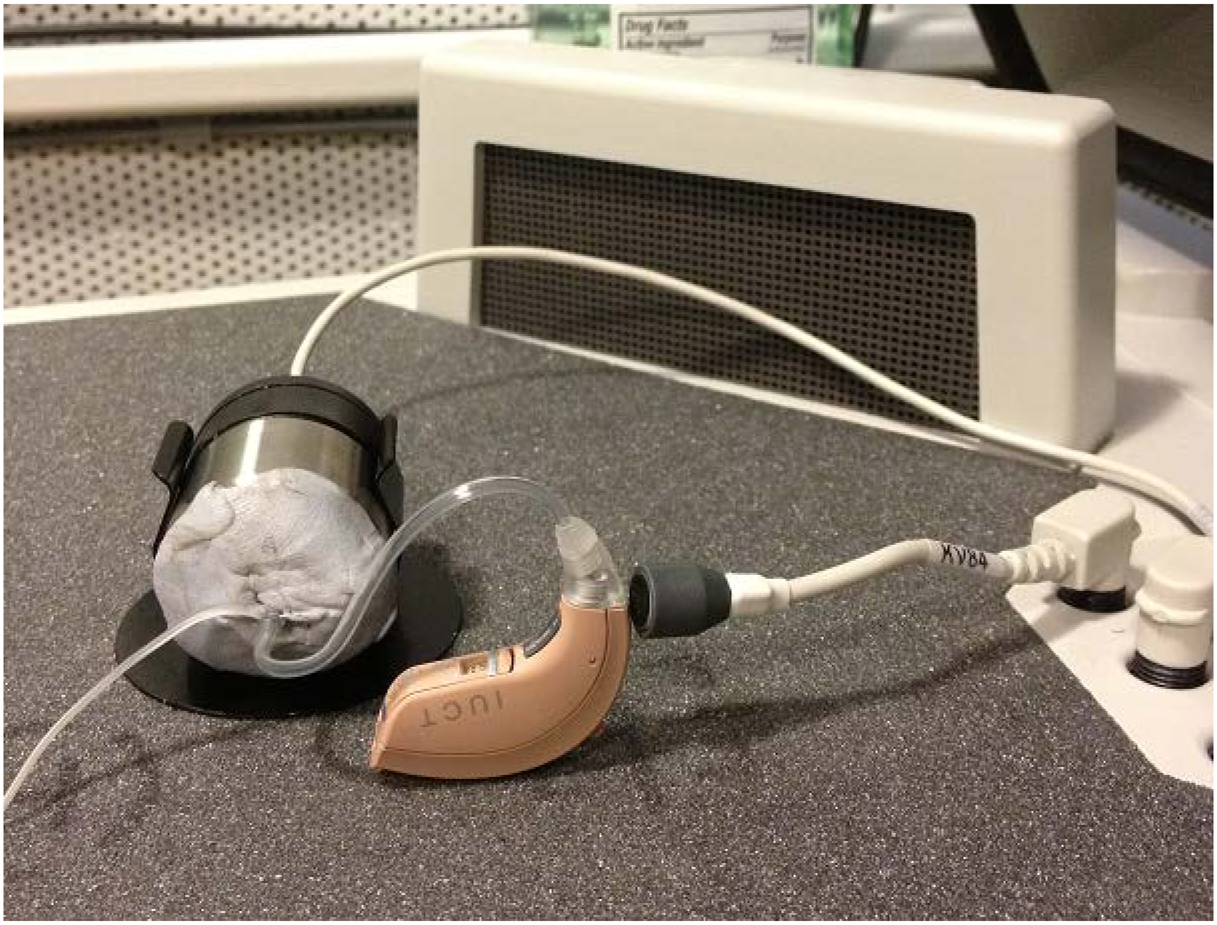

e. Click "Connect" with Airlink.
f. When logging on to a patient's name that has already had a hearing aid programmed to it, it will give you an error box that gives you an option to "Replace Instruments". Click this option.
g. Click "unassign" under previously connected instruments, then click "find" to connect to your new hearing aid (you may or may not have to open/close battery door again)
h. Once the hearing aid has been detected you will have to assign it to the **right** ear before you can proceed then press "done".
i. If prompted, select "Keep current session's fitting data".
j. Check set-up of P1 prior to running speechmapping:
    i. P1 should be set on "Basic+Softswitching" if it is not already on that setting.
        1. In "Advanced Features"
            a. Directionality should be FIXED
            b. Directional Mix: Very Low
            c. DFS Ultra: **Moderate** (this is often defaulted to Mild)
            d. Expansion: Off
            e. Noise Tracker II: Per Environment
            f. Wind Guard: Off
        2. In "Environmental Optimizer" all values should be at zero.
        3. (7/7/15) If applicable click on "Tinnitus Sound Generator" and ensure it is OFF
        4. Physical Properties
            a. X: 2A, 10mm aid dome
            b. Y and Z: 2A, Tulip dome

k. On Verifit, click on "tests" → "test box measures" →"Speechmapping"

i. Targets:

X	Targets								
**Freq.**	**250**	**500**	**750**	**1000**	**1500**	**2000**	**3000**	**4000**	**6000**
**Level**	52	61	62	58	54	55	57	59	52

Y	Targets								
**Freq.**	**250**	**500**	**750**	**1000**	**1500**	**2000**	**3000**	**4000**	**6000**
**Level**	50	62	65	65	62	60	64	68	60

Z	Targets								
**Freq.**	**250**	**500**	**750**	**1000**	**1500**	**2000**	**3000**	**4000**	**6000**
**Level**	44	62	64	63	64	65	72	74	64

ii. Run speechmapping at 65 dB SPL. Make fine tuning changes as necessary to meet appropriate targets (above).

1. Change view from "graph" to "table" for fine-tuning.
2. When fine tuning, select the frequency(ies) that need/s to be changed (G50 and G80 should both be selected)
3. **Target-Matching Rule:** For 500 THROUGH 4000 Hz, all frequencies within 2 dB of target at ALL frequencies in this range.
4. Once you are finished fine tuning, run speechmapping at 55 and 75 dB SPL
5. Run MPO (defaults to 90 dB SPL)
6. Print measurements to USB (can rename file now as X(YorZ)2CCsn)

l. Set up programs 2-4 based on hearing aid programming (after copying and pasting P1 to all programs, decrease or increase overall gain based on chart below):

**CD Group**	**P1**	**P2**	**P3**	**P4**
**X (HFPTA: 19 dB)**	0	-2	-4	+2
**Y (HFPTA: 34 dB)**	0	-4	-8	+4
**Z (HFPTA: 44 dB)**	0	-5	-10	+5

m. Click on "Summary" (top next to "Fit") and select save to instrument only in the bottom right corner.

i. When a box pops up that prompts you to "use auto relate to apply gain adjustments from one program to the other programs" click **NO**

n. Exit out of Aventa without saving to the database.

o. Transfer USB to Laptop to rename files from the Verifit. Use the file naming sheet to name each file appropriately. Put manufacturer's specs in CD group folder in cabinet.

10. **Shell modifications**:

a. Put silver paint pen stripe over "Resound" and "GN Hearing"

b. Apply black paint pen over AL967-DIW leaving the serial number visible

c. Immediately cover it with the silver paint pen, and let dry for a couple of minutes.

d. Finally, finish by covering with a silver sharpie marker and a layer of clear nail polish to seal.

e. CD GROUP: Put post-it on case labeling X, Y, or Z. Place hearing aids on corresponding shelf by color in unmarked case with triangular adaptors inside.

f. AB GROUP: Place hearing aids on ANSI side of shelf under corresponding color in unmarked case with triangular adaptors inside.
